# *SPOP* mutations increase PARP inhibitor sensitivity via CK2/PIAS1/SPOP axis in prostate cancer

**DOI:** 10.1172/jci.insight.186871

**Published:** 2025-04-22

**Authors:** Hui Zhang, Lili Kong, Jinhui Li, Zhihan Liu, Yiting Zhao, Xiuyi Lv, Liangpei Wu, Lin Chai, Hongjie You, Jiabei Jin, Xinyi Cao, Zhong Zheng, Yadong Liu, Zejun Yan, Xiaofeng Jin

**Affiliations:** 1 Department of Urology, The First Affiliated Hospital of Ningbo University, Ningbo, Zhejiang, China.; 2 Department of Biochemistry and Molecular Biology, Health Science Center, Ningbo University, Ningbo, Zhejiang, China.; 3 Department of Ultrasound Medicine, The Affiliated People’s Hospital of Ningbo University, Ningbo, Zhejiang, China.

**Keywords:** Cell biology, Genetics, Oncology, DNA repair, Drug therapy, Prostate cancer

## Abstract

It is well documented that impaired DNA damage repair (DDR) induces genomic instability that can efficiently increase the sensitivity of prostate cancer (PCa) cells to PARP inhibitors; however, the underlying mechanism remains elusive. Here, we found profound genomic instability in PCa cells with *SPOP* gene mutations and confirmed the sensitivity of *SPOP*-mutated PCa cells to olaparib-induced apoptosis. Mechanistically, we identified olaparib-induced CK2-mediated phosphorylation of PIAS1-S468, which in turn mediated SUMOylation of SPOP, thus promoting its E3 ligase activity in the DDR. Moreover, an abnormal CK2/PIAS1/SPOP axis due to *SPOP* mutations or defects in CK2-mediated phosphorylation of PIAS1, as well as SPOP inhibitor treatment, led to impaired DDR, thus increasing olaparib-induced apoptosis of PCa cells and enhancing olaparib sensitivity in animal models and patient-derived organoids. This suggested that disruption of the CK2/PIAS1/SPOP signaling axis could serve as an indicator for targeted therapy of PCa using a PARP inhibitor.

## Abstract 2

Disrupting the CK2-PIAS1-SPOP signaling axis, including *SPOP* mutations, as well as CK2/SPOP inhibitor increases the efficacy of PARP inhibitor in PCa.

## Graphical abstract

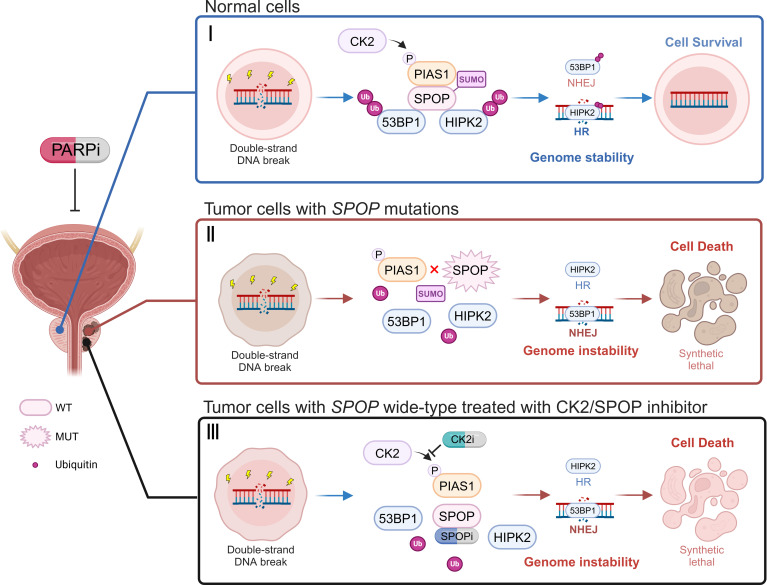

## Introduction

Prostate cancer (PCa) has become a significant cause of male disability and mortality, with recent studies predicting a continued increase in both incidence and mortality rates over the next 20 years (1). The treatment options for early-stage PCa are diverse, including radiation therapy and castration therapy, such as hormone therapy and surgery (2). However, approximately 30%–40% of PCa cases progress to castration-resistant prostate cancer (CRPC), rendering these therapies ineffective. Therefore, new treatment protocols are required to complement existing treatments (2).

High-frequency mutations in homologous recombination repair–related (HRR-related) genes occur in CRPC, such as breast cancer gene 1/2 (*BRCA1/2*) and ataxia telangiectasia mutated (*ATM*) genes (2). PCa cells with mutations in HRR-related genes confer high genomic instability and exhibit elevated sensitivity to DNA damage therapies such as poly(ADP-ribose) polymerase (PARP) inhibitors (3, 4). A multicenter retrospective study involving 131 patients with PCa found that speckle-type POZ protein (*SPOP*) gene mutations are critical predictors of a favorable treatment response to PARP inhibitors; however, the underlying mechanism is unclear (5). Upon DNA damage, we and other groups have revealed that SPOP promotes the nondegradative ubiquitination of DNA damage repair–related (DDR-related) substrates, such as homeodomain-interacting protein kinase 2 (HIPK2) and p53-binding protein 1 (53BP1), accelerating DDR to maintain genomic stability (6–9). PCa with *SPOP* mutations may exhibit defects in DDR mediation, potentially rendering it sensitive to PARP inhibitors (5, 10, 11). However, the underlying regulations of SPOP to initiate DDR and the potential target to inhibit the DDR process contributing to cancer cell apoptosis remain largely unknown.

Current evidence has not yet provided a clear understanding of how SPOP responds promptly and accurately to DNA damage. The levels of posttranslational modifications of SPOP during DDR may offer an explanation; however, only a limited number of studies have indicated that ATM-mediated phosphorylation enhances the response of SPOP to DNA damage (6, 7, 12, 13). Notably, a substantial body of evidence indicates that cellular SUMOylation levels significantly increase in response to DNA damage, suggesting a crucial role for SUMOylation in DDR (14–16). Additionally, our review and other studies have suggested that SPOP is highly likely to be SUMOylated under DNA-damaging conditions, which may explain its timely response to DDR (8, 17–19). However, the specific SUMO E3 ligase that mediates the SUMOylation of SPOP upon DDR as well as the role of SPOP SUMOylation in DDR remains unknown.

In this study, we first elucidated the high-frequency mutations of the *SPOP* gene in PCa and the associated genomic instability, as well as sensitivity of *SPOP*-mutated PCa cells to olaparib-induced apoptosis. Second, we discovered that upon exposure to olaparib-induced DNA damage, casein kinase 2 (CK2) mediated the phosphorylation of protein inhibitor of activated STAT 1 (PIAS1), and phosphorylated PIAS1 binds to SPOP and promotes SUMOylation of SPOP at lysine residues 53, 110, and 129, thereby facilitating SPOP-associated DDR. Intriguingly, PCa-associated SPOP mutants and SUMOylation site–mutated SPOP mutants evade PIAS1-mediated SUMOylation, resulting in enhanced nonhomologous end joining (NHEJ) and deficient HR, thereby leading to cell apoptosis. Moreover, in cell-derived xenograft (CDX) models, olaparib exhibited more pronounced inhibition of tumor growth in CDX models carrying *SPOP* mutations. Correspondingly, PCa patient–derived organoid models harboring *SPOP* mutations also demonstrated sensitivity to olaparib, revealing the significant role of the PIAS1/SPOP axis in DDR. Finally, utilization of a CK2 inhibitor and SPOP inhibitor enhanced the therapeutic efficacy of olaparib in CDX and organoid models with wild-type SPOP (SPOP-WT). Our study indicates a promising treatment strategy for PARP inhibitors in PCa patients with *SPOP* mutations or a combination of CK2 inhibitor and SPOP inhibitor for PCa patients with SPOP-WT.

## Results

### The high-frequency mutations of the SPOP gene in PCa lead to genomic instability, resulting in the sensitivity of PCa cells to olaparib-induced apoptosis.

**Figure 1:**
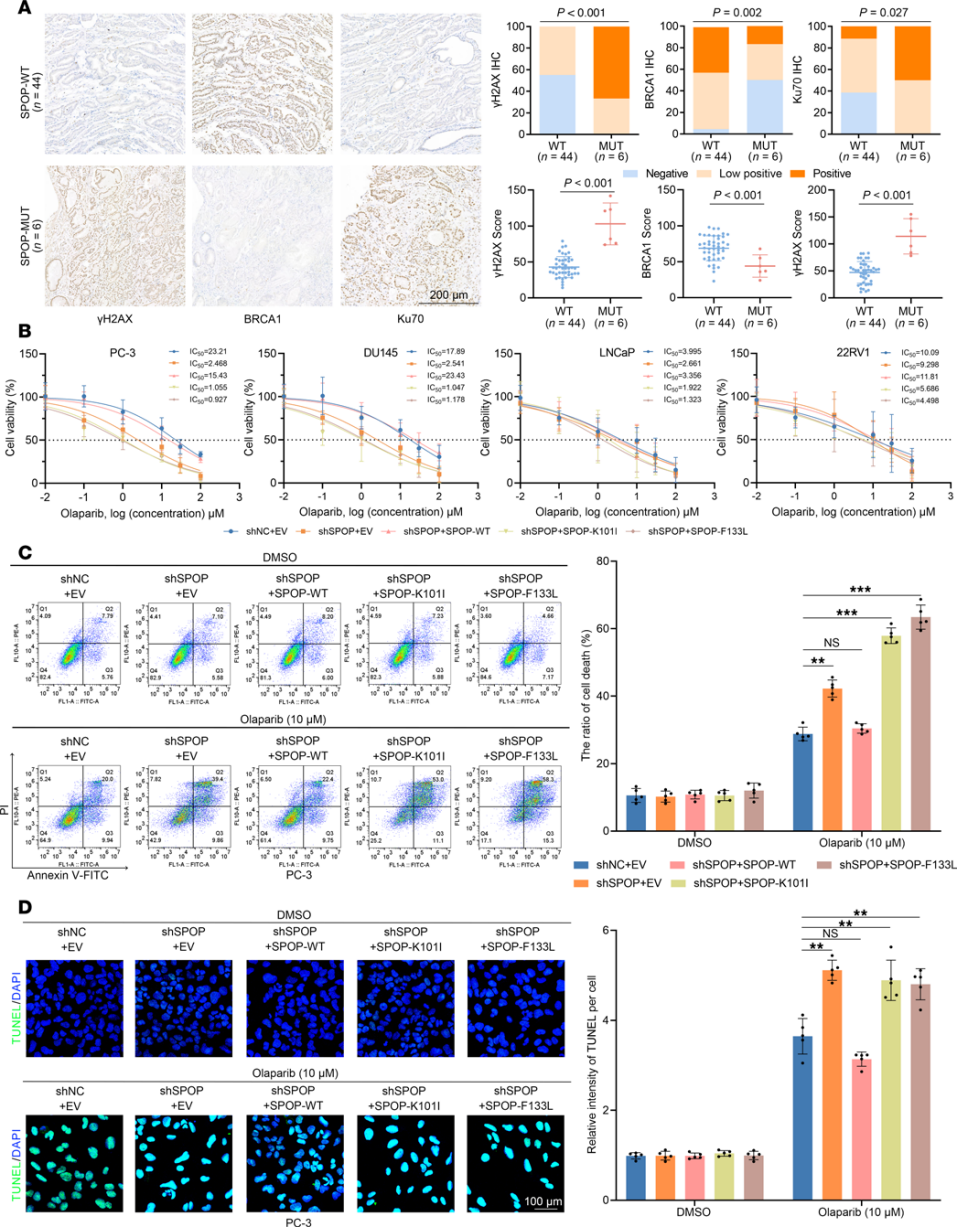
*SPOP*-mutated PCa cells are sensitive to olaparib-induced apoptosis. (**A**) Representative images of IHC staining for γH2AX/BRCA1/Ku70 (left) in PCa tissues with SPOP-WT group (*n* = 44) and SPOP-MUT (*n* = 6), and the graphs showing staining intensities and scores (right). Scale bar: 200 μm. (**B**) Cell viability assays were performed to calculate the IC_50_ values of PC-3, DU145, LNCaP, and 22RV1 cells in each group treated with different concentrations of olaparib (0.01, 0.1, 1, 10, 30, and 100 μM). (**C**) Representative flow cytometry histograms (left) and graphs (right, *n* = 5) showing the apoptosis levels of PC-3 cells in each group induced by olaparib (10 μM). (**D**) Representative images (left) and graph (right, *n* = 5) of TUNEL staining showing the apoptosis levels of PC-3 cells in each group induced by olaparib (10 μM). Scale bar: 100 μm. Data are shown as mean ± SD. **P < 0.01; ***P < 0.001 by 2-tailed, unpaired Student’s *t* test.

*SPOP* gene mutations occur in 10%–15% of PCa cases and are predominantly located within the substrate-binding MATH domain (8) (Supplemental Figure 1A; data from COMSIC, https://cancer.sanger.ac.uk/cosmic; supplemental material available online with this article; https://doi.org/10.1172/jci.insight.186871DS1). We collected 50 PCa tissue specimens after surgery with corresponding clinicopathological features (Supplemental Table 1). Genomic DNA was extracted from PCa tissue samples and specific primers were designed to amplify exons 6 and 7 of the *SPOP* gene (corresponding to the MATH domain; Supplemental Figure 1B). Subsequently, DNA sequencing was performed to identify 6 PCa patients harboring *SPOP* genes (c.268C>G, L90V; c.302A>T, K101I; c.373G>T, F125V; c.385A>G, K129E; c.399C>G, F133L; and c.399C>A, F133L) with a mutation rate of approximately 12% (6 out of 50; Supplemental Figure 1C). PCa with *SPOP* mutations exhibits high genomic instability, specifically characterized by the HR deficiency and NHEJ overactivation in DDR. Therefore, we stained γH2AX (DDR marker), BRCA1 (HR marker), and Ku70 (NHEJ marker) in the collected PCa specimens and analyzed the intensity of IHC staining (Figure 1A). Indeed, we observed enhanced γH2AX and Ku70 staining as well as weakened BRCA1 staining in the *SPOP*-mutated PCa, suggesting genomic instability in *SPOP*-mutated PCa and potential sensitivity to PARP inhibitors.

Given that genomic instability may improve the sensitivity of PCa cells to olaparib treatment, we constructed stable PCa cell lines, including 2 androgen receptor–negative (AR-negative) PCa cell lines (PC-3 and DU145) and 2 AR-postive (LNCaP and 22RV1) cell lines, carrying the SPOP-WT or SPOP mutants (SPOP-K101I and -F133L), respectively, to test this hypothesis. Utilizing cell viability assays to determine the IC_50_ values of olaparib in each group of PCa cells, we found that *SPOP* knockdown significantly reduced the IC_50_ values of olaparib in PC-3 and DU145 cells, but moderately reduced those in LNCaP and 22RV1 cells (Figure 1B). Notably, the SPOP-K101I and -F133L mutants showed a further decrease in IC_50_ values of all PCa cell lines, indicating a dominant negative function (Figure 1B). Given that PARP inhibitors are more commonly applied in CRPC, PC-3 and DU145 were studied as the primary cell models in subsequent functional assays.

PARP inhibitors cause DNA damage within cells, ultimately leading to apoptosis. Therefore, we assessed apoptosis changes in PCa cells treated with olaparib using flow cytometry, TUNEL staining, and apoptosis-related markers (cleaved caspase-7) by Western blotting. Based on the IC_50_ values obtained from Figure 1B, we assessed the apoptosis level of PCa cells PC-3 and DU145, as well as LNCaP cells, after 24 hours of treatment and found that PCa cells with *SPOP* knockdown exhibited increased apoptosis under olaparib treatment, whereas PCa cells carrying SPOP-K101I/F133L exhibited more pronounced apoptosis (Figure 1, C and D, and Supplemental Figure 1, D–F). Taken together, our data demonstrate the sensitivity of *SPOP*-mutated PCa cells to olaparib-induced apoptosis.

### Upon treatment with olaparib, PIAS1 may be involved in SPOP-associated DDR and the response of SPOP to olaparib-induced apoptosis.

To elucidate the mechanism underlying SPOP involvement in the response to olaparib treatment, we first established stable PC-3 cell lines overexpressing 3×FLAG-SPOP. Subsequently, we obtained intracellular 3×FLAG-SPOP protein complexes before and after olaparib treatment by immunoprecipitation (IP) (Supplemental Figure 2A). SPOP protein complexes were analyzed by mass spectrometry (MS) to determine the protein abundance. By comparing the SPOP protein complex before and after olaparib treatment, we identified proteins whose interactions with SPOP were altered by olaparib (Supplemental Figure 2A). Furthermore, olaparib-resistant PCa cell lines were constructed and subjected to proteomic analysis along with their corresponding parental cell lines to identify proteins associated with olaparib resistance (Supplemental Figure 2B). Finally, by generating Venn diagrams, we identified proteins that exhibited significant changes in abundance in both sets of experiments (Supplemental Figure 2C). We found that the protein abundance of PIAS1 was significantly increased in both experimental groups (Supplemental Figure 2C). Similarly, there was a significant increase in the abundance of HIPK2 and 53BP1, which are key nondegradative substrates of SPOP involved in DDR (Supplemental Figure 2C).

PIAS1 is a SUMO E3 ligase that participates in cell signaling, cell cycle regulation, and DDR by mediating the SUMOylation modification of substrates. The role of PIAS1 as a tumor suppressor or promoter has not been conclusively determined. The Cancer Genome Atlas (TCGA) database analysis indicates a significant decrease in mRNA expression levels of *PIAS1* in PCa tissues compared with normal tissues (Supplemental Figure 3A). However, *PIAS1* knockdown in PC-3 and DU145 cells did not significantly affect the proliferation and migration capabilities of these PCa cells (Supplemental Figure 3, B and C), nor did it affect the tumor formation of PC-3 cells in CDX models (Supplemental Figure 3D), suggesting that PIAS1 may not be a major tumor suppressor or promoter under normal physiological conditions. However, IHC staining for γH2AX and PIAS1 proteins in SPOP-WT PCa tissues revealed a significant negative correlation between the protein levels of PIAS1 and γH2AX, suggesting that PIAS1 was involved in maintaining genomic stability (Supplemental Figure 3E). Next, we stained the nuclear BRCA1 (a marker of HR) and Ku70 foci (a marker of NHEJ) in PCa cells to determine the specific role of PIAS1 in the DDR. *PIAS1* knockdown resulted in the suppression of HR and activation of NHEJ following treatment with olaparib, especially at the 8-hour time point. At the 24-hour time point, the remaining activated Ku70 foci and BRCA1 foci may indicate a delay in the overall DDR process in the *PIAS1*-knockdown group (Supplemental Figure 3, F and G), indicating that PIAS1 maintains genomic stability by promoting HR while inhibiting NHEJ to facilitate DDR, similar to the reported roles of SPOP in the PCa DDR process (6, 7). Thus, we speculated that PIAS1 may collaborate with SPOP to participate in the therapeutic response to olaparib. Indeed, *PIAS1* knockdown promoted olaparib-induced apoptosis, which was suppressed by SPOP overexpression in PC-3 cells (Supplemental Figure 3, H and I).

### CK2 mediates phosphorylation of PIAS1-Ser468, enhancing its interaction with SPOP under olaparib treatment.

**Figure 2:**
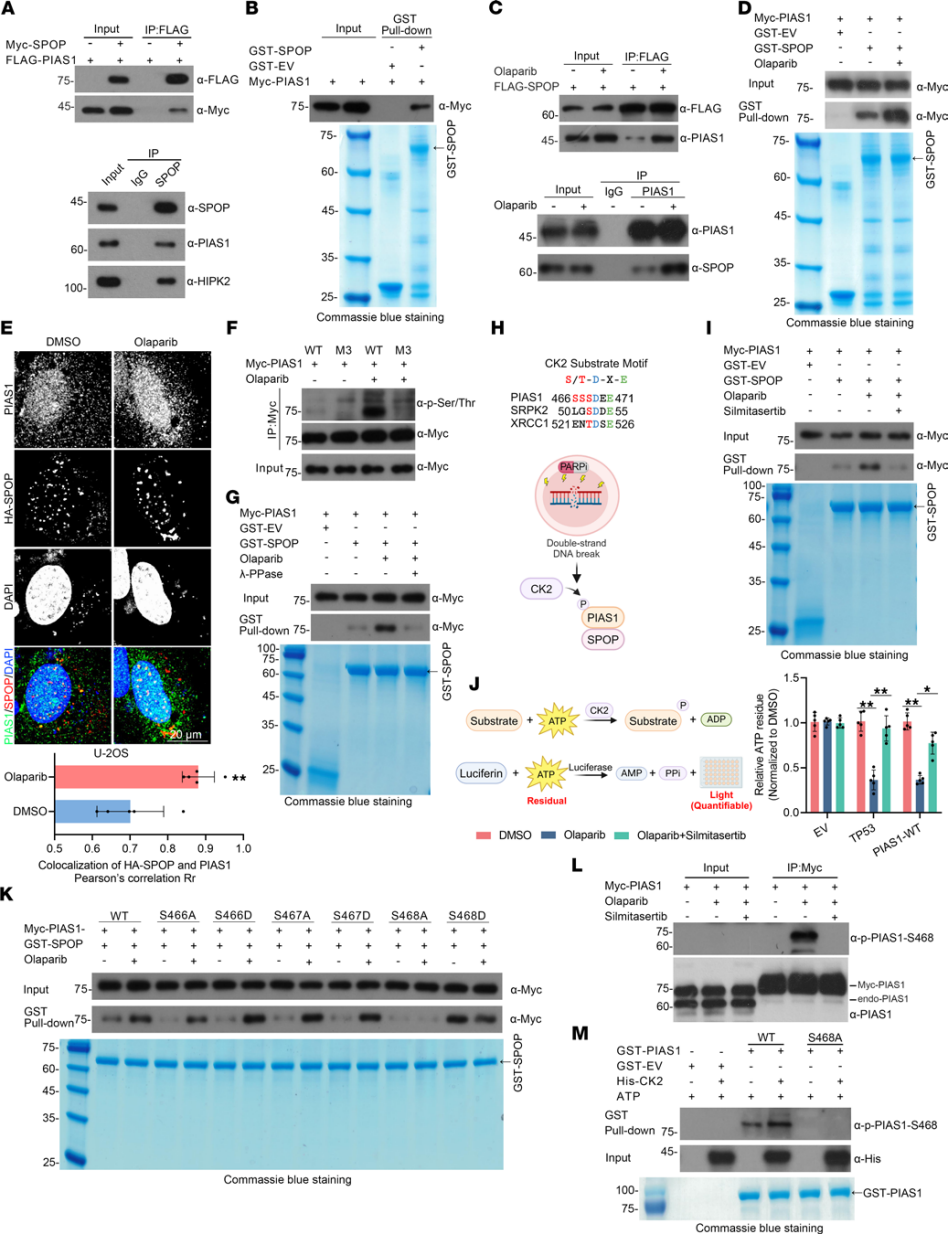
Olaparib induces CK2-mediated phosphorylation of PIAS1-S468, increasing PIAS1-SPOP interactions. (**A**) In vivo interaction assays in HEK-293T cells with exogenous (top) and endogenous (bottom) PIAS1 and SPOP protein levels showing the interaction between PIAS1 and SPOP. (**B**) In vitro interaction assays utilizing cell lysates from Myc-PIAS1–transfected HEK-293T cells and purified GST-SPOP or GST from BL21 (DE3) competent cells. (**C**) In vivo interaction assays of exogenous (top) and endogenous (bottom) protein levels from HEK-293T cells showing the enhanced interaction between PIAS1 and SPOP under olaparib (10 μM) treatment. (**D**) In vitro interaction assays utilizing cell lysates from Myc-PIAS1–transfected HEK-293T cells treated with olaparib (10 μM) or not and purified GST-SPOP or GST from BL21 (DE3) competent cells. (**E**) Top: Representative immunofluorescence images of U-2OS cells transfected with HA-SPOP plasmid and/or not treated with olaparib (10 μM), stained for HA-SPOP (green), PIAS1 (red), and with DAPI (blue). Scale bar: 20 μm. Bottom: Quantitative analysis (*n* = 5) of colocalization between SPOP and PIAS1 through Image-Pro Plus 6.0 software. (**F**) Co-IP utilizing cell lysates from Myc-PIAS1-WT– or Myc-PIAS1-M3–transfected HEK-293T cells treated with olaparib or not, stained with a pan-phosphorylation antibody. (**G**) In vitro interaction assays utilizing phosphatase-treated cell lysates from Myc-PIAS1–transfected HEK-293T cells treated with olaparib (10 μM) or not. (**H**) Top: Diagram showing CK2 substrate motif in PIAS1. Bottom: CK2 mediates phosphorylation of PIAS1 to promote the interaction between PIAS1 and SPOP under olaparib treatment. Created in BioRender. (**I**) In vitro interaction assays utilizing cell lysate from Myc-PIAS1–transfected HEK-293T cells treated with olaparib (10 μM) and CK2 inhibitor (silmitasertib) or not. (**J**) Left: Working model of luminescence-based kinase assay. Created in BioRender. Right: Kinase activity assays utilizing purified CK2 from HEK-293T cells treated with olaparib or not, and silmitasertib or not, as well as purified GST-EV, TP53, and PIAS1 proteins from BL21 cells. (**K**) In vitro interaction assays utilizing cell lysates from Myc-PIAS1-WT– or Myc-PIAS1-MUT–transfected HEK-293T cells treated with olaparib (10 μM) or not. (**L**) Co-IP utilizing cell lysates from Myc-PIAS1–transfected HEK-293T cells treated with olaparib and silmitasertib or not, stained with anti-PIAS1 and anti–PIAS1-Ser468 antibodies. (**M**) In vitro interaction assays utilizing purified GST-PIAS1-WT, GST-PIAS1-S468A, GST-EV, and His-CK2 from BL21 (DE3) competent cells. Top: Western blot with anti–PIAS1-Ser468 and anti-His antibodies. Bottom: Coomassie blue –stained gel. Data are shown as mean ± SD. *P < 0.05; **P < 0.01 by 2-tailed, unpaired Student’s *t* test.

To elucidate the underlying mechanism by which PIAS1 participates in SPOP-associated DDR and response to olaparib treatment, we sought to identify the interaction between PIAS1 and SPOP in vivo and in vitro. We demonstrated an interaction between PIAS1 and SPOP at both endogenous and exogenous levels in PC-3 cells in vivo (Figure 2A). Furthermore, GST pull-down (in vitro) confirmed the direct interaction between PIAS1 and SPOP (Figure 2B). Notably, the interaction between PIAS1 and SPOP was significantly enhanced at both exogenous and endogenous levels in PC-3 cells (Figure 2C). The enhancement of the interaction was also observed at the in vitro level (Figure 2D), and immunofluorescence showed an increase in the colocalization of PIAS1 and SPOP, especially in the nucleus, after olaparib treatment (Figure 2E). Thus, we confirmed that the interaction between PIAS1 and SPOP is dramatically enhanced by olaparib treatment.

We then identified key domains/motifs involved in the interaction between SPOP and PIAS1. Similar to previous reports (8), SPOP lacking the substrate-binding domain (MATH domain, SPOP-ΔMATH) lost its interaction with PIAS1 (Supplemental Figure 4, A and B). Additionally, proteins that interact with SPOP often contain one or several SPOP-binding consensus (SBC) motifs (Φ-π-S/T-S/T-S/T, where Φ represents nonpolar residues and π polar residues; Supplemental Figure 4C). We examined the PIAS1 protein sequence and identified 2 potential SBC motifs (^464^IDSSS^468^ and ^582^PYTSS^586^; Supplemental Figure 4D). Potential SBC motif–mutated PIAS1 mutants (PIAS1-M1/M2/M3) were generated and tested for their interaction with SPOP (Supplemental Figure 4D). Co-IP and GST pull-down assays showed that both SBC motifs were required for the binding of PIAS1 to SPOP (Supplemental Figure 4, D and E).

Upon DNA damage, various kinases are required to respond to stress by mediating substrate phosphorylation and initiating the DDR process. In addition, previous studies have shown that phosphorylation of SBC motifs of substrates can affect their interactions (8, 20). To determine whether phosphorylation of the SBC motifs affected the interaction between PIAS1 and SPOP, we initially assessed the changes in PIAS1-WT and PIAS1-M3 phosphorylation levels upon olaparib treatment. After Myc-PIAS1-WT or -M3 enrichment and pan-phosphorylation antibody detection, we found that the phosphorylation level of Myc-PIAS1-WT, but not Myc-PIAS1-M3, was markedly elevated following olaparib treatment (Figure 2F). In addition, we treated the cell lysates with λ protein phosphatase (λ-PPase), and GST pull-down assay revealed that the olaparib-enhanced interaction between SPOP and PIAS1 was dramatically reduced, indicating that phosphorylation of PIAS1 at its SBC motifs may enhance the SPOP-PIAS1 interaction (Figure 2G). To identify the kinase responsible for PIAS1 phosphorylation in cells treated with olaparib, we first performed a search using 2 web servers, NetPhos 3.1 and PhosphoNET, both of which predicted protein kinase CK2 as the top candidate (Table 1). Notably, a consensus motif (S/T-D-X-E) of the CK2 phosphorylation site was identified near the N-terminal SBC motif of PIAS1 (Figure 2H). To experimentally validate this prediction, we used silmitasertib (CX-4945), a potent and highly selective chemical inhibitor of CK2, which has been tested in clinical trials (ClinicalTrials.gov NCT03904862) (21, 22), to confirm the role of CK2-mediated phosphorylation of PIAS1. We found a marked decrease in the interaction between PIAS1 and SPOP, which was enhanced by olaparib treatment, suggesting that CK2-mediated phosphorylation indeed promoted the interaction between PIAS1 and SPOP under olaparib treatment (Figure 2, H and I). Furthermore, we demonstrated that olaparib mediates enhancement of CK2 kinase activity through in vitro kinase activity assays (Figure 2J). Indeed, olaparib did activate CK2 kinase, as confirmed by ATP consumption by TP53 (a positive control) (23, 24) and PIAS1 compared with that in the group without olaparib treatment, while silmitasertib could dramatically reverse the olaparib-induced CK2 kinase activation enhancement (Figure 2J).

Given the potential phosphorylation sites of PIAS1 (Ser466, Ser467, and Ser468) (25), we generated a series of mutants, including phosphomimetic mutants (S466D, S467D, and S468D) and phospho-null mutants (S466A, S467A, and S468A). We found that changes in the phosphorylation of Ser468 significantly reduced the interaction between SPOP and PIAS1 under olaparib treatment (Figure 2K). Therefore, an anti–p- PIAS1-Ser468 antibody was custom-made to verify CK2-mediated phosphorylation of PIAS1 induced by olaparib (Figure 2L). We found that olaparib indeed induced the phosphorylation of Myc-PIAS1 at Ser468, which could be reversed by silmitasertib (Figure 2L). Moreover, in vitro phosphorylation experiments confirmed that CK2 kinase directly mediates the phosphorylation of Myc-PIAS1 at Ser468 (Figure 2M). In line with these results, olaparib-activated CK2 kinase–mediated phosphorylation of PIAS1 was also observed in PCa cell lines PC-3, DU145, and LNCaP (Supplemental Figure 4F).

### PIAS1-mediated SUMOylation of SPOP enhances SPOP-associated DDR.

**Figure 3:**
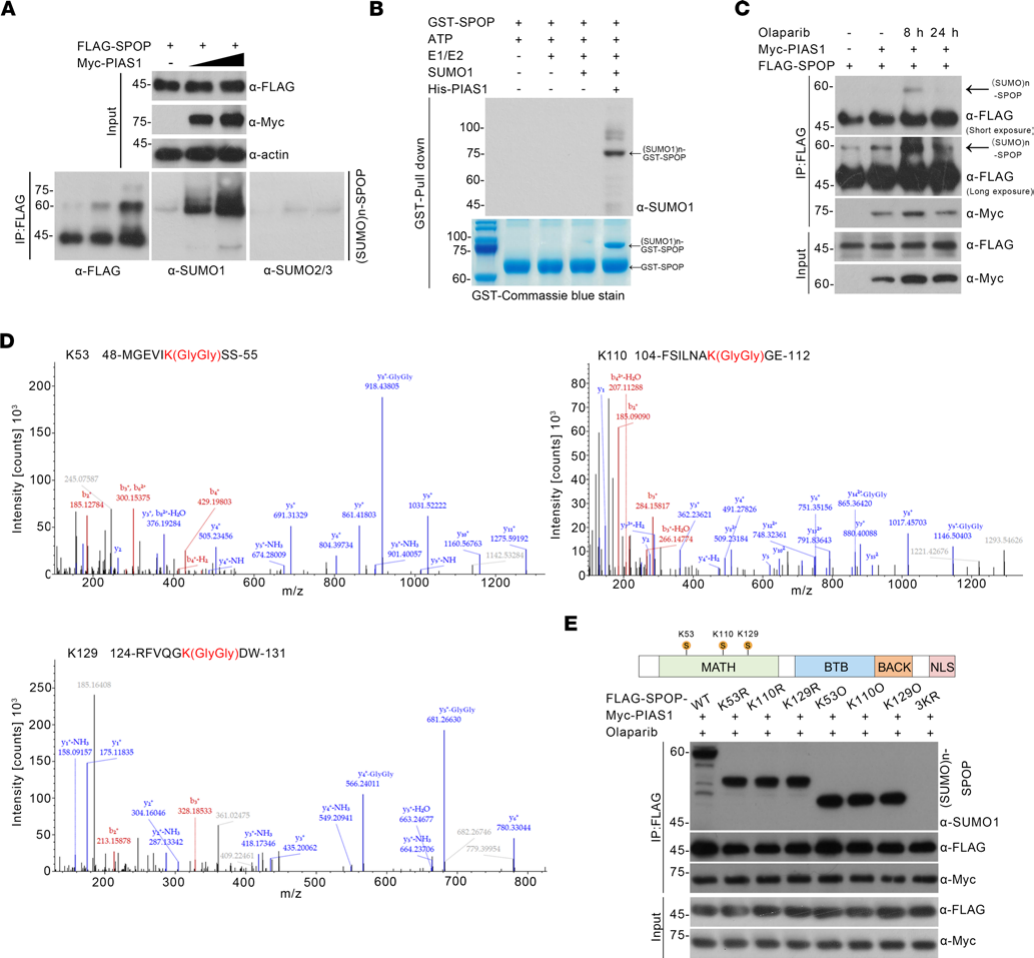
PIAS1 mediates SUMOylation of SPOP upon DNA damage. (**A**) In vivo SUMOylation assays showing the PIAS1-mediated SUMOylation of SPOP using lysates from HEK-293T cells transfected with FLAG-SPOP and increased dosage of Myc-PIAS1 plasmids, staining with anti-FLAG, anti-SUMO1, anti-SUMO2/3, anti-Myc, and anti-actin antibodies. (**B**) In vitro SUMOylation assays using reconstituted His-PIAS1, E1 and E2 enzymes, ATP, SUMO1, and GST-SPOP to show the PIAS1-mediated SUMOylation of SPOP. (**C**) In vivo SUMOylation assays showing the PIAS1-mediated enhanced SUMOylation of SPOP under olaparib (10 μM) treatment using lysates from HEK-293T cells transfected with FLAG-SPOP and Myc-PIAS1 plasmids, staining with anti-FLAG and anti-Myc bodies. (**D**) Mass spectrometry analysis showing potential SUMOylation site of SPOP. (**E**) In vivo SUMOylation assays showing the PIAS1-mediated SUMOylation of SPOP under olaparib (10 μM) treatment using lysates from HEK-293T cells transfected with FLAG-SPOP-WT or FLAG-SPOP-MUT and Myc-PIAS1 plasmids, staining with anti-FLAG, anti-Myc, and anti-SUMO1 antibodies.

Given that SPOP acts as a key E3 ubiquitin ligase, we hypothesized that SPOP mediates the ubiquitination of PIAS1 following olaparib treatment. However, an in vivo ubiquitination assay showed that SPOP did not mediate ubiquitination and degradation of PIAS1 (Supplemental Figure 4, G–I). Therefore, we tested whether PIAS1 could serve as a SUMO E3 ligase. Indeed, PIAS1 mediates SUMOylation of SPOP, which is predominantly SUMOylated by SUMO1 (Figure 3A). To demonstrate the reliability of PIAS1-mediated SUMO1 modification of SPOP, we conducted in vitro SUMOylation assays, which further confirmed that PIAS1 primarily mediates SUMOylation of SPOP with SUMO1 (Figure 3B). Additionally, the SUMOylation level of SPOP was dramatically increased at the 8-hour time point, suggesting that PIAS1-mediated SUMOylation of SPOP is associated with the early stages of DDR (Figure 3C). Moreover, potential SUMOylation sites (K53, K110, and K129) on SPOP modified by PIAS1 were revealed through MS analysis (Figure 3D); correspondingly, SUMOylation site–mutated SPOP mutants (K→R) were generated. In vivo SUMOylation assays suggested that all K53, K110, and K129 sites were involved in PIAS1-mediated SUMOylation (Figure 3E), while the mutations at the SUMOylation sites did not affect the PIAS1-SPOP interaction (Figure 3E), indicating that all SUMOylation site–mutated SPOP mutants (SPOP-3KR) could be utilized to study the PIAS1/SPOP axis–associated DDR process (Figure 3E).

**Figure 4:**
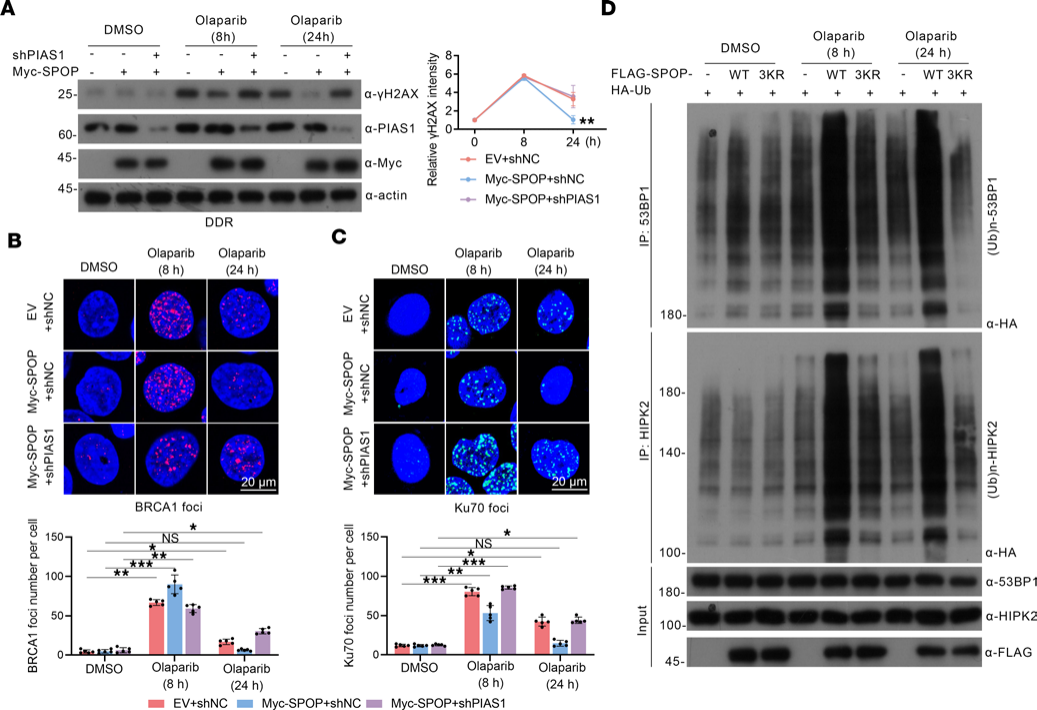
PIAS1-mediated SUMOylation of SPOP enhances SPOP-induced DDR in olaparib treatment. (**A**) Western blotting (left) and quantification (right) of γH2AX showing the DDR process. (**B**) BRCA1 (HR marker) staining (top) and corresponding graph (bottom, *n* = 5). (**C**) Ku70 foci (NHEJ marker) were stained (top) and corresponding graph (bottom, *n* = 5). Scale bars: 20 μm. (**D**) In vivo ubiquitination assays utilizing cell lysates from FLAG-SPOP-WT– or FLAG-SPOP-3KR–transfected HEK-293T cells treated with olaparib or not, stained with HA, 53BP1, HIPK2, and FLAG antibodies. Data are shown as mean ± SD. *P < 0.05; **P < 0.01; ***P < 0.001 by 2-tailed, unpaired Student’s *t* test.

To identify the impact of PIAS1-mediated SUMOylation on SPOP-associated DDR, we first tested changes in the SPOP-induced DDR process by *PIAS1* knockdown following olaparib treatment. Evaluation of γH2AX expression intensity demonstrated that *PIAS1* knockdown delayed the SPOP-mediated DDR process (Figure 4A). A decrease in the number of BRCA1 foci (Figure 4B) and an increase in the number of Ku70 foci (Figure 4C) were observed, suggesting inhibition of HR and activation of NHEJ. To address the underlying mechanism, we enriched the SPOP protein complexes after olaparib treatment and *PIAS1* knockdown (Supplemental Figure 5A) and found distinct changes in the interaction of SPOP with its associated substrates, especially HIPK2 and 53BP1 (Supplemental Figure 5B). In vivo ubiquitination assays showed that *PIAS1* knockdown inhibited the SPOP-mediated ubiquitination of HIPK2 and 53BP1 following olaparib treatment (Supplemental Figure 5C). Additionally, we found that the SPOP-3KR–mediated (SUMOylation-deficient mutant) ubiquitination of HIPK2 and 53BP1 was dramatically decreased, highlighting the role of the PIAS1-mediated SUMOylation of SPOP in DDR (Figure 4D). Together, these results suggest that PIAS1-mediated SUMOylation of SPOP enhances SPOP-associated DDR in PCa cell models.

SPOP enhances its interaction with substrates and promotes ubiquitination through self-oligomerization. To test this notion, we sought to observe SPOP speckle changes under olaparib treatment, and an increase in the size of the SPOP speckle under olaparib treatment was observed, which could be counteracted by *PIAS1* knockdown (Supplemental Figure 5D). Additionally, co-IP revealed that olaparib treatment enhanced the self-oligomerization of SPOP, whereas this effect disappeared with *PIAS1* knockdown (Supplemental Figure 5E). Moreover, SPOP-3KR exhibited impaired enhancement of self-oligomerization upon olaparib treatment, suggesting that the PIAS1-mediated SUMOylation of SPOP may influence the formation of SPOP speckles and the interaction between SPOP and substrates (Supplemental Figure 5G).

### Dysregulation of the CK2/PIAS1/SPOP axis impairs DDR.

**Figure 5:**
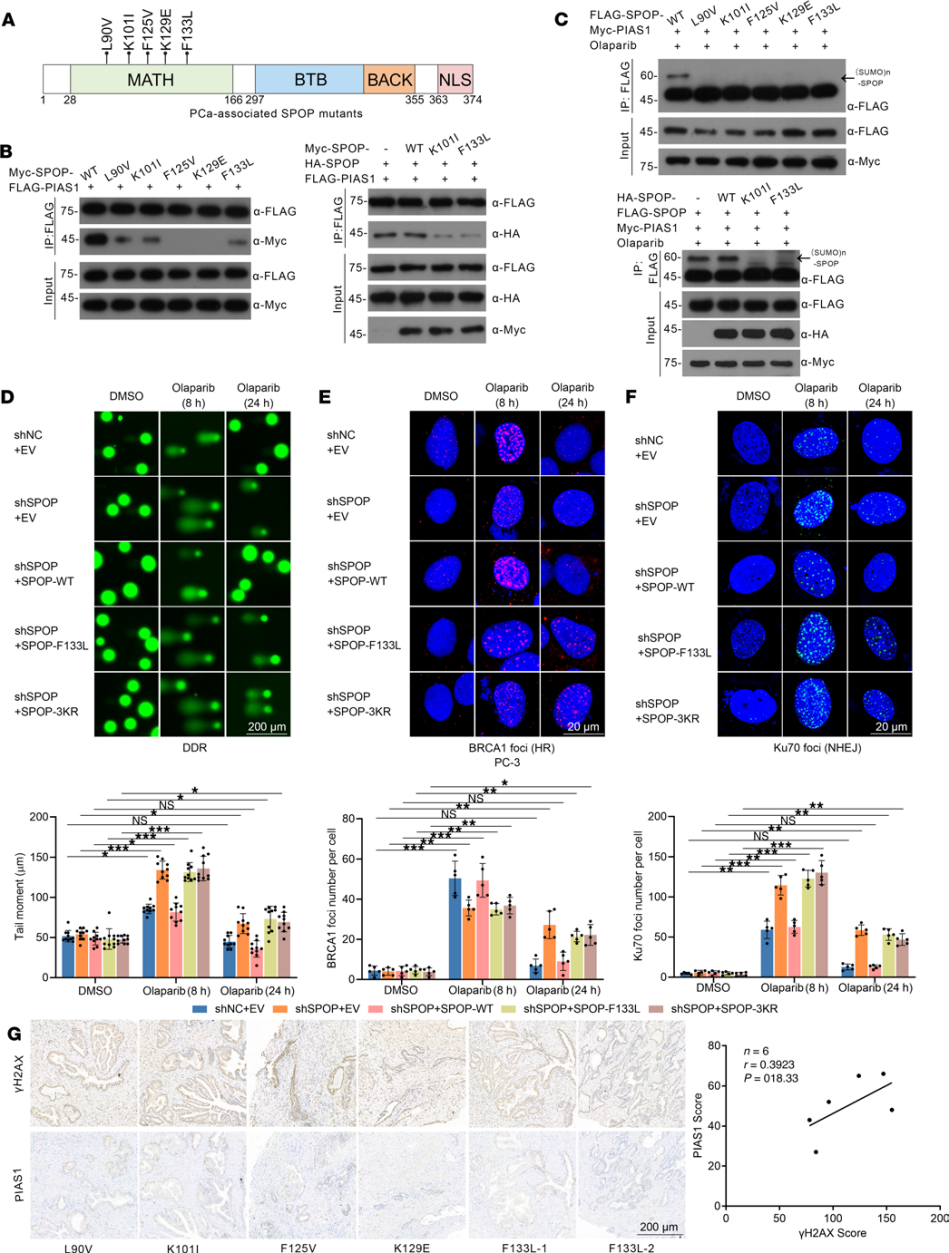
Dysregulation of CK2/PIAS1/SPOP axis leads to impaired DDR in PC-3 cells. (**A**) Diagram showing PCa-associated SPOP mutants. (**B**) Co-IP showing that SPOP mutants exhibit defects in their interaction with PIAS1 (left), and even inhibits the interaction between SPOP-WT and PIAS1 (right). (**C**) In vivo SUMOylation assays showing that PCa-associated SPOP mutants escape PIAS1-mediated SUMOylation (top) and inhibit PIAS1-mediated SUMOylation of SPOP-WT (bottom). (**D**) The comet assays revealed the overall DDR process in PC-3 cells under olaparib treatment (10 μM), and the representative images (top) and graph (bottom, *n* = 10) are shown. The comet assays were then performed and 10 cells from each sample were analyzed based on the tail moment, utilizing Komet software. (**E**) BRCA1 foci were stained (top) and the corresponding graph (bottom, *n* = 5) under olaparib (10 μM) treatment to determine the HR process in PC-3 cells. (**F**) Ku70 foci were stained (top) and corresponding graph (bottom, *n* = 5) under olaparib (10 μM) treatment to determine the NHEJ process in PC-3 cells. (**G**) The images of IHC staining for γH2AX and PIAS1 (left) in each *SPOP*-mutated PCa tissue, along with the corresponding statistical analysis of staining intensity (right); *n* = 6, *r* = 0.4813, *P* = 0.3338. Scale bars: 200 μm (**D** and **G**) and 20 μm (**E** and **F**). Data are shown as mean ± SD. *P < 0.05; **P < 0.01; ***P < 0.001 by 2-tailed, unpaired Student’s *t* test.

We generated SPOP mutants (L90V, K101I, F125V, K129E, and F133L) detected in PCa patients and analyzed the impact of these mutants on the PIAS1/SPOP axis (Supplemental Figure 1C and Figure 5A). In vivo interaction assays demonstrated that PCa-associated SPOP mutants lost their interaction with PIAS1 under olaparib treatment and exhibited a dominant negative effect on SPOP-WT (Figure 5B). Furthermore, in vivo SUMOylation assays showed that SPOP mutants evaded SUMOylation mediated by PIAS1 and simultaneously inhibited the SUMOylation of SPOP-WT by PIAS1 (Figure 5C).

Next, we explored the effect of SPOP mutants SPOP-F133L (highest frequency) and SPOP-3KR on DDR related to the PIAS1/SPOP axis. We used comet assays to assess the overall DDR process, as well as analysis of BRCA1 and Ku70 foci, to detect the components of HR and NHEJ in DDR. Comet assays revealed that under olaparib treatment, SPOP-F133L and SPOP-3KR resulted in compromised DDR processes (Figure 5D), with suppression of HR (Figure 5E and Supplemental Figure 6A) and excessive activation of NHEJ (Figure 5F and Supplemental Figure 6B), suggesting the significance of PIAS1-mediated SUMOylation of SPOP in DDR (Figure 5, D and E, and Supplemental Figure 6, A–C). Additionally, a negative correlation between PIAS1 and γH2AX scores was observed in *SPOP*-WT PCa tissue groups (Supplemental Figure 3E), but not in *SPOP*-mutated PCa tissue groups (Figure 5G), suggesting that the role of PIAS1 in regulating genomic stability is associated with SPOP.

**Figure 6:**
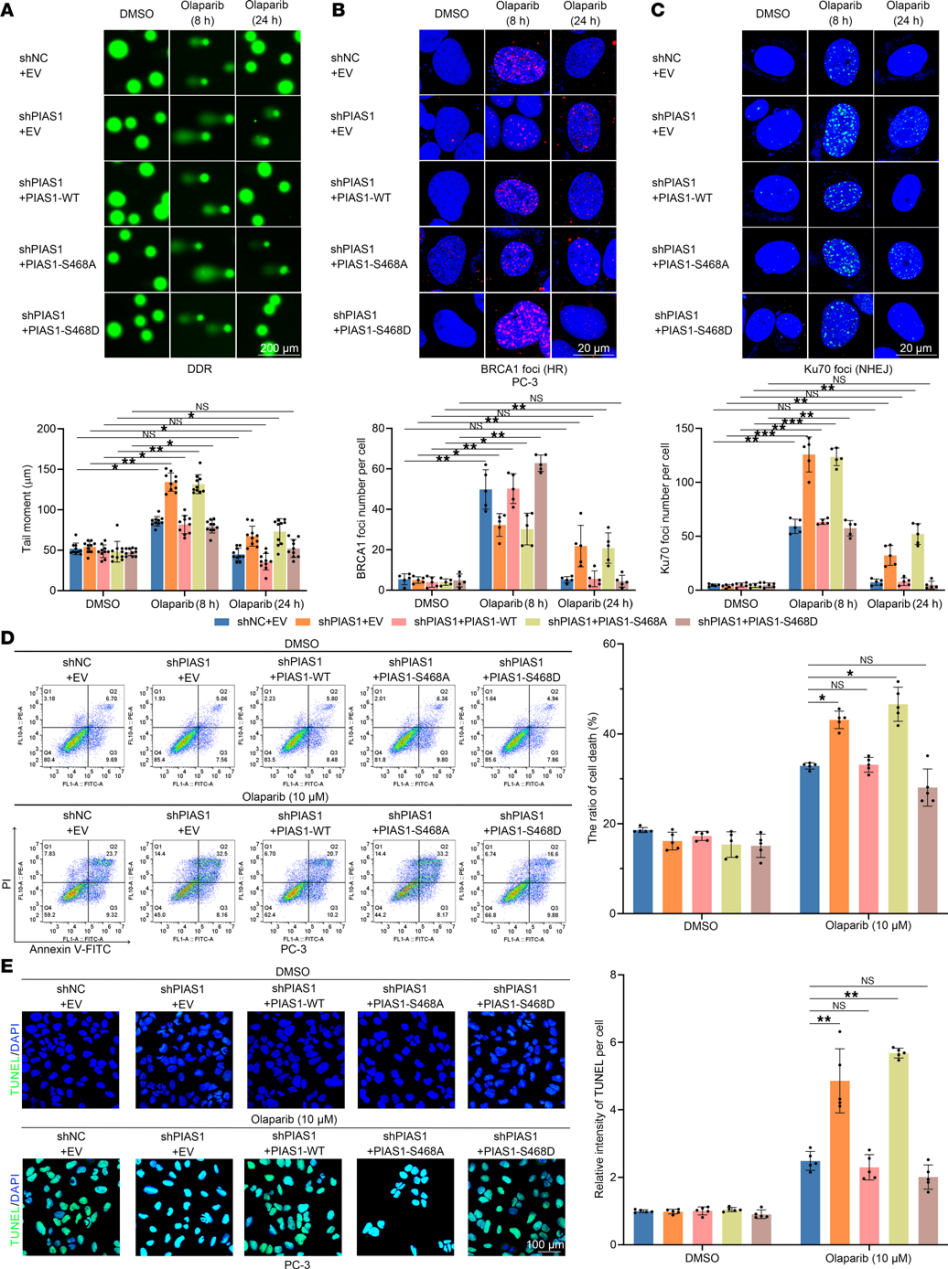
CK2-mediated phosphorylation of PIAS1-S468 increases DDR and inhibits olaparib-induced apoptosis. (**A**) The comet assays revealed the overall DDR process in PC-3 cells under olaparib treatment (10 μM), and the representative images (top) and graph (bottom, *n* = 10) are shown. The comet assays were then performed and 10 cells from each sample were analyzed based on the tail moment, utilizing Komet software. (**B**) BRCA1 foci were stained (top) and corresponding graph (bottom, *n* = 5) under olaparib (10 μM) treatment to determine the HR process in PC-3 cells. (**C**) Ku70 foci were stained (top) and corresponding graph (bottom, *n* = 5) under olaparib (10 μM) treatment to determine the NHEJ process in PC-3 cells. (**D**) Rep resentative flow cytometry histograms (left) and graph (right, *n* = 5) showing the apoptosis levels of PC-3 cells in each group induced by olaparib (10 μM). (**E**) Representative images (left) and graph (right, *n* = 5) of TUNEL staining showing the apoptosis levels of PC-3 cells in each group induced by olaparib (10 μM). Scale bars: 200 μm (**A**), 20 μm (**B** and **C**), and 100 μm (**E**). Data are shown as mean ± SD. *P < 0.05; **P < 0.01; ***P < 0.001 by 2-tailed, unpaired Student’s *t* test.

Additionally, phosphorylation site–mutated PIAS1 mutants (S468A/D) were studied to show their impact on DDR. The phospho-null PIAS1 mutant (S468A) similarly exhibited compromised DDR (Figure 6A) and HR processes (Figure 6B and Supplemental Figure 6D) along with hyperactive NHEJ (Figure 6C and Supplemental Figure 6E), whereas the phosphomimetic PIAS1 mutant (S468D) showed enhanced DDR and HR processes (Figure 6, A–C, and Supplemental Figure 6, D and E), suggesting the importance of CK2-mediated phosphorylation of PIAS1 in DDR. We also assessed the responses of PC-3 and DU145 cells to olaparib-induced apoptosis at different levels of PIAS1 phosphorylation. Apoptosis markers (Supplemental Figure 6G), flow cytometry (Figure 6D), and TUNEL staining (Figure 6E and Supplemental Figure 6H) indicated that PC-3 cells carrying PIAS1-S468A were more sensitive to olaparib-induced apoptosis, whereas those carrying PIAS1-S468D were resistant to olaparib (Figure 6, D and E, and Supplemental Figure 6, G and H), further suggesting the importance of CK2-mediated phosphorylation of PIAS1 in DDR and the response of PCa cells to olaparib. This may offer the potential to interfere with the phosphorylation levels of PIAS by targeting CK2 to assist in olaparib treatment.

In summary, our data confirmed that dysregulation of the CK2/PIAS1/SPOP axis impairs DDR in PCa cells under olaparib treatment, potentially explaining the underlying reason why *SPOP*-mutated PCa cells exhibit more pronounced apoptosis under olaparib treatment (Figure 1 and Supplemental Figure 1).

### Dysregulation of the PIAS1/SPOP axis affects the response of CDX models and organoid models to olaparib treatment.

**Figure 7:**
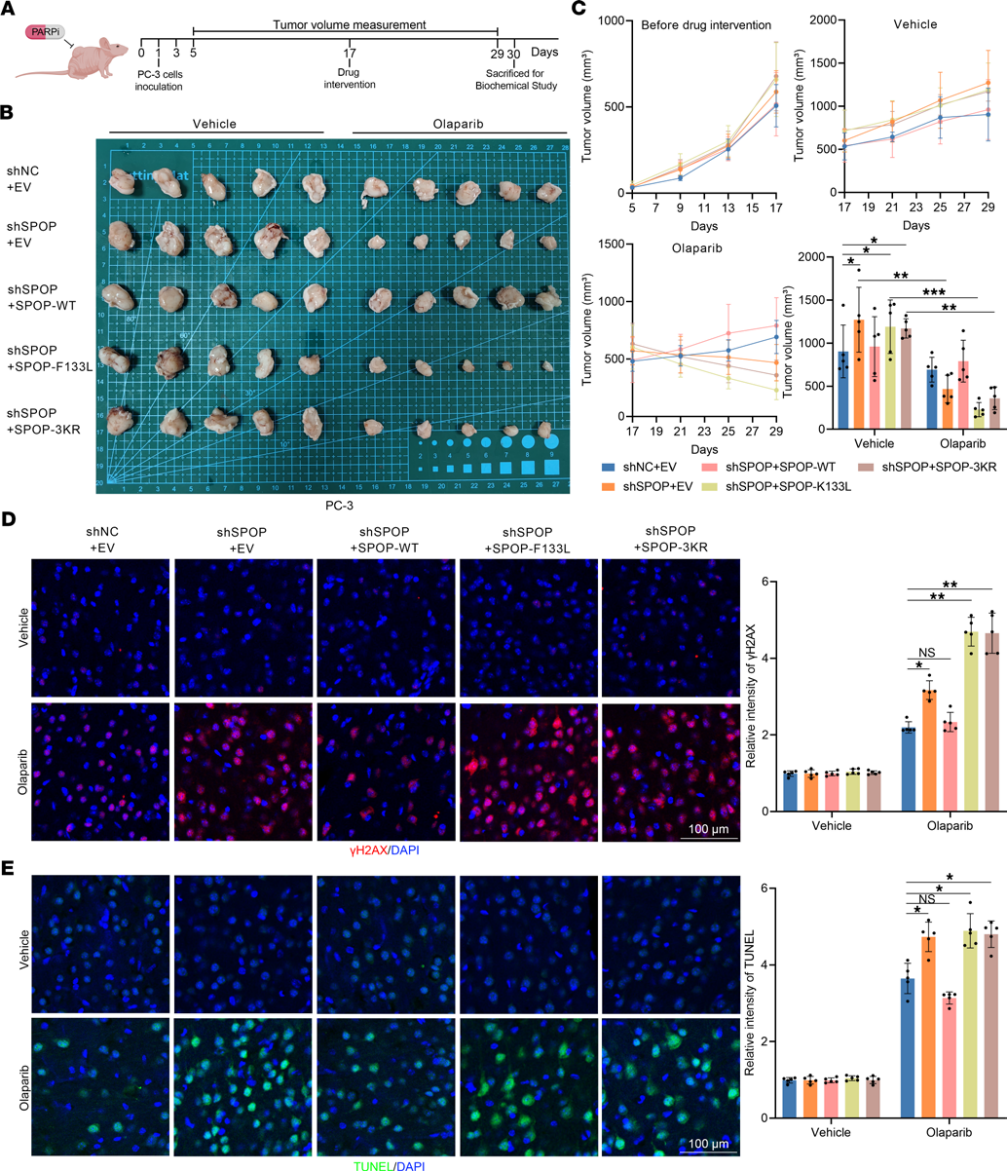
Dysregulation of the PIAS1/SPOP axis affects the response of CDX models to olaparib treatment. (**A**) The flowchart for detecting the sensitivity of the CDX models to olaparib. Created in BioRender. (**B**) Each group of PC-3 cells was inoculated into the right flank of BALB/c nude mice, which were then treated with vehicle or olaparib (50 mg/kg). Tumor growth was measured every 4 days. On the 17th day after PC-3 cell inoculation, each group of CDX models (*n* = 10) was divided into 2 groups: vehicle (*n* = 5) and olaparib (*n* = 5), to assess the sensitivity of each group of CDX models to olaparib treatment. Tumors in each group were harvested on day 30 and photographed. (**C**) The tumor growth curves and statistical graph of tumor volume before and after olaparib treatment. (**D**) Representative images of γH2AX staining (left) and the graph (right) of γH2AX staining intensity for each group. (**E**) Representative images of TUNEL staining (left) and the graph (right) of TUNEL staining intensity for each group. Scale bars: 100 μm. Data are shown as mean ± SD. *P < 0.05; **P < 0.01; ***P < 0.001. The differences between 2 groups were analyzed using unpaired, 2-tailed Student’s *t* test, and multiple comparisons were performed using 2-way ANOVA.

We then sought to determine the sensitivity of PCa to olaparib due to disruption of the PIAS1/SPOP signaling axis in the CDX models. PC-3 cells were injected into the right dorsal neck of BALB/c nude mice, and tumor volume was measured every 3 days. When the tumors in each group grew to approximately 500 mm^3^, mice in each group (*n* = 10) were divided into 2 groups (*n* = 5 each). One group was administered normal saline (vehicle) via gavage, and the other group was administered olaparib (50 mg/kg). Gavage administration was conducted once a day for 14 days, and tumor growth was observed in the CDX model (Figure 7A). First, we observed that PC-3 cells with *SPOP* knockdown and carrying a PCa-associated SPOP mutant (F133L) exhibited stronger tumorigenicity, which is consistent with previous reports (8) on *SPOP* gene mutations as pathogenic factors in PCa (Figure 7, B and C). Similarly, SPOP-3KR cells showed enhanced tumorigenicity (Figure 7, B and C). After olaparib treatment, CDX models with *SPOP* knockdown and SPOP-F133L and SPOP-3KR exhibited significant tumor regression, suggesting sensitivity to olaparib treatment (Figure 7, B and C). Tumor tissues were collected and subjected to IHC staining for γH2AX (Figure 7D) and TUNEL staining (Figure 7E). The results revealed that after olaparib treatment, tumor tissues from CDX models with *SPOP* knockdown and SPOP-F133L and SPOP-3KR exhibited a pronounced increase in DNA damage and apoptosis.

**Figure 8:**
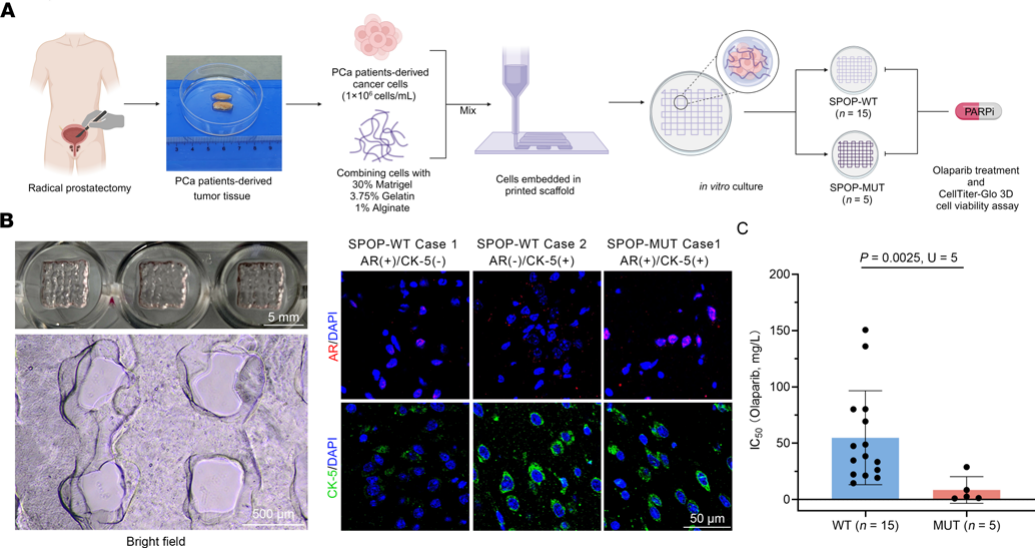
Dysregulation of the PIAS1/SPOP axis affects the response of organoid models to olaparib treatment. (**A**) Flowchart illustrating the workflow for constructing 3DP-POs. Created in BioRender. (**B**) Left: Bright-field images of 3DP-POs. Right: CK-5 and AR staining (right) of Case 1 and Case 2 in the SPOP-WT group, and Case 1 in the SPOP-MUT group. Scale bars: 5 mm (top), 500 μm (bottom), and 50 μm (right). (**C**) The statistical analysis of IC_50_ values of olaparib for 3DP-POs, including the SPOP-WT group (*n* = 15) and SPOP-MUT group (*n* = 5). Data in **C** were assessed with the Mann-Whitney *U* test.

In addition, we constructed 3D patient tumor avatars derived from patients with PCa through 3D bioprinting, named 3D bioprinted PCa organoids (3DP-POs), to further confirm the sensitivity of *SPOP*-mutated PCa to olaparib (Figure 8, A and B). The clinical pathological features of each PCa patient used for constructing the 3DP-POs are provided in the supplemental Supporting Data Values file. We performed calcein-AM/PI staining and 3D cell viability assays at multiple time points (days 1, 3, 5, and 7) during the model construction to ensure the survival and proliferation of PCa cells (Supplemental Figure 7, A and B). Given that 3DP-POs carry the patient’s native genetic background and pathological features, we investigated cytokeratin 5 (CK-5) and ARs by IHC staining of 3DP-POs, as well as the PSA secretion in the culture medium of 3DP-POs. Notably, CK-5 and AR staining and PSA secretion corresponded well with the pathological data from clinical patients (Figure 8B, Supplemental Figure 7C, and Table 2), suggesting that 3DP-POs indeed recapitulated the pathological features of the patients. Because of the limited quantity of tumor tissue and tumor cells obtained from the surgical resection of PCa patients, we were unable to perform individual staining for all cases; therefore, 3DP-POs were primarily used for measuring the IC_50_ values of olaparib. IC_50_ values were plotted to reflect the 3DP-POs’ sensitivity to olaparib using the CellTiter-Glo 3D Cell Viability Assay (Supplemental Figure 8A). At the same time, we matched a portion of the 3DP-POs with BRCA1 staining of PCa tissue samples from Figure 1 (Supplemental Figure 8A). A strong positive correlation was observed between the IC_50_ values of the 3DP-POs and the intensity of BRCA1 staining (Supplemental Figure 8B). Compared with 3DP-POs with SPOP-WT, 3DP-POs carrying *SPOP* mutations (including F133L, etc.) had significantly lower IC_50_ values (Figure 8C). Altogether, these results indicate that dysregulation of the PIAS1/SPOP axis increases the response to olaparib treatment in CDX models and organoid models.

### Targeting the CK2/PIAS1/SPOP axis contributes to the treatment sensitivity of PCa to olaparib.

**Figure 9:**
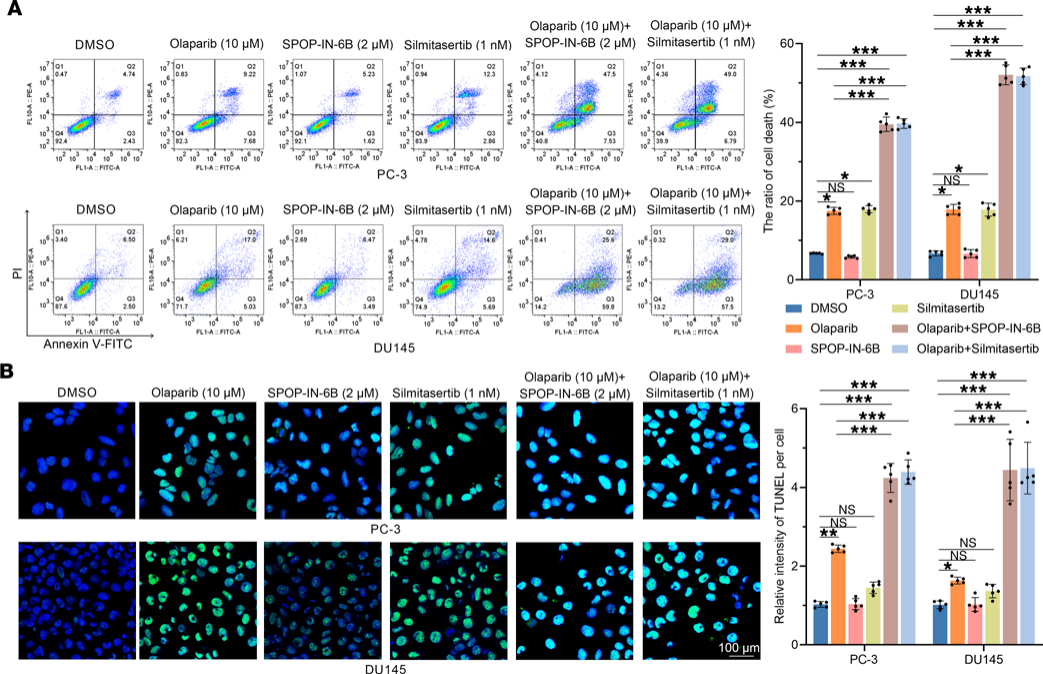
Targeting the CK2/PIAS1/SPOP axis contributes to the treatment sensitivity of PCa cells to olaparib. (**A**) Representative flow cytometry histograms (left) and graph (right, *n* = 5) showing the apoptosis levels of PC-3 cells in each group induced by olaparib (10 μM), in combination or not with SPOP-IN-6b (2 μM) or silmitasertib (1 nM). (**B**) Representative images (left) and graph (right, *n* = 5) of TUNEL staining showing the apoptosis levels of PC-3 cells in each group induced by olaparib (10 μM), in combination or not with SPOP-IN-6b (2 μM) or silmitasertib (1 nM). Scale bar: 100 μm. Data are shown as mean ± SD. *P < 0.05; **P < 0.01; ***P < 0.001 by unpaired, 2-tailed Student’s *t* test.

**Figure 10:**
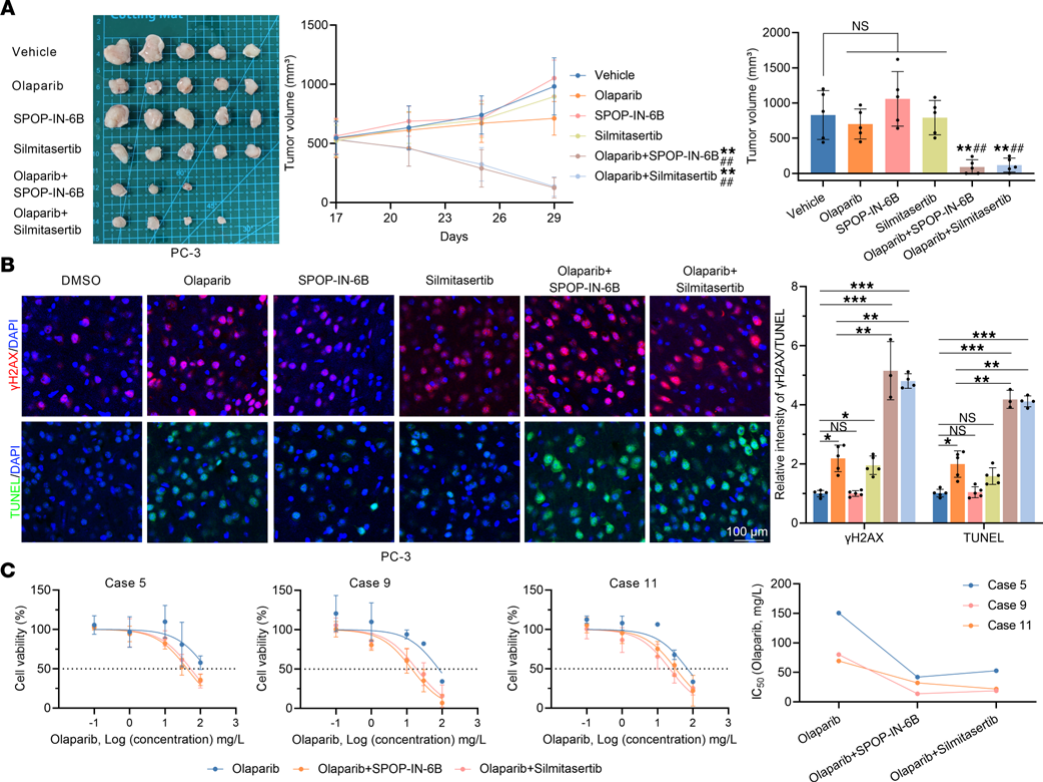
Targeting the CK2/PIAS1/SPOP axis contributes to the treatment sensitivity of CDX and organoid models to olaparib. (**A**) PC-3 cells were inoculated into the right flank of BALB/c nude mice (*n* = 30). Tumor growth was measured every 4 days. On the 17th day after PC-3 cell inoculation, mice were divided into 6 groups (*n* = 5 each) and treated with vehicle or olaparib (50 mg/kg), in combination or not with SPOP-IN-6b (40 mg/kg) or silmitasertib (25 mg/kg). Tumors in each group on day 30 were harvested and photographed. (**B**) Representative images of γH2AX and TUNEL staining (left) and the graph (right) of γH2AX and TUNEL staining intensity for each group. Scale bar: 100 μm. Data are shown as mean ± SD. In **A**, **P < 0.01 vs. vehicle; ^##^*P* < 0.01 vs. olaparib by 2-tailed Student’s *t* test. In **B**, *P < 0.05, **P < 0.01, ***P < 0.001 vs. vehicle by 2-tailed Student’s *t* test. The differences between 2 groups were analyzed using unpaired, 2-tailed Student’s *t* test, and multiple comparisons were performed using 2-way ANOVA. (**C**) The 3DP-POs were treated with olaparib in combination or not with SPOP-IN-6b (2 μM) or silmitasertib (1 nM), and the IC_50_ values of the 3DP-POs to olaparib were determined.

Given that olaparib was largely ineffective against CDX models and 3DP-POs with SPOP-WT (Figures 7 and 8), we explored whether artificially modulating the CK2/SPOP/PIAS1 axis could create “synthetic lethality,” thus enhancing the efficacy of olaparib. To investigate this, we attempted to augment olaparib treatment of PCa using a CK2 inhibitor (silmitasertib) and an SPOP inhibitor (SPOP-IN-6b) (26). Flow cytometry and TUNEL staining revealed that SPOP-IN-6b and silmitasertib sharply enhanced olaparib-induced apoptosis (Figure 9, A and B). Furthermore, in SPOP-WT CDX models, the combined use of SPOP-IN-6b or silmitasertib resulted in more dramatic tumor regression under olaparib treatment, with some CDX models even exhibiting complete tumor disappearance (Figure 10A), with higher levels of DNA damage and apoptosis, confirming that silmitasertib or SPOP-IN-6b indeed increased the efficacy of olaparib treatment (Figure 10B). Additionally, in 3DP-POs, we selected models from 3 patients with SPOP-WT (cases 5, 9, and 11) and found that silmitasertib and SPOP-IN-6b also markedly reduced the IC_50_ values of olaparib (Figure 10C). Together, these findings suggest that manipulating the CK2/SPOP/PIAS1 axis to assist olaparib treatment could potentially create a “synthetic lethality,” offering improved outcomes for patients with PCa receiving olaparib treatment.

## Discussion

Based on these findings, we reached the following conclusions: (a) Under normal physiological conditions, olaparib-induced DNA damage promotes CK2 kinase–mediated PIAS1-S468 phosphorylation, contributing to the interaction between PIAS1 and SPOP. Notably, these interactions trigger PIAS1-mediated SUMOylation of SPOP (K53, K110, and K129), mainly in a SUMO1-associated modification, thus enhancing SPOP-associated DDR. (b) In *SPOP*-mutated PCa, SPOP mutants evade olaparib-induced PIAS1-mediated SUMOylation, leading to a delay in DDR and triggering “synthetic lethality” apoptosis in PCa cells. (c) By interfering with the CK2/PIAS1/SPOP signaling axis using CK2 inhibitors and SPOP inhibitors, a “synthetic lethality” effect can be artificially created, impairing DDR in PCa cells with SPOP-WT and promoting olaparib-induced apoptosis.

Recently, some biomarkers have been considered indicative of sensitivity to PARP inhibitors in PCa, such as *BRCA1/2* gene mutations and defects in HR deficiency (27). Although several studies have uncovered potential biomarkers for PARP inhibitor sensitivity, few have progressed to clinical applications (9). In 2014, the first study discovered that SPOP is involved in DDR (28), and subsequent research revealed that SPOP promotes error-free HR (7), suppresses error-prone NHEJ (6, 9), and prevents excessive DNA replication (13) to maintain genomic stability. Therefore, the impairment of DDR and the resulting high genomic instability due to *SPOP* gene mutations may contribute to the sensitivity of PCa to PARP inhibitors, which was partially supported by a clinical retrospective study (5) as well as by cellular and animal model studies, but not yet at the clinical level (9, 10, 29). Our study represents the first evidence to our knowledge at the organ level (3DP-POs) in patients supporting the sensitivity of *SPOP*-mutated PCa to PARP inhibitors, providing stronger evidence for the future clinical application of PARP inhibitors in PCa patients with *SPOP* mutations, as well as SPOP-WT, together with inhibitors targeting the CK2/PIAS1/SPOP signaling axis.

Upon DNA damage, we found a pronounced role for the CK2/PIAS1/SPOP signaling axis in DDR. Specifically, the CK2/PIAS1/SPOP signaling axis promotes error-free HR but suppresses error-prone NHEJ. Notably, errors in DDR resulting from excessive activation of NHEJ are also recognized as markers of sensitivity to PARP inhibitors (30). The potential reason is that DNA repaired through NHEJ has been reported to be more prone to subsequent damage, thereby reactivating the DDR process (30), which partly explains why Ku70 foci and residual BRCA1 foci at the 24-hour time point were observed in our results due to disruption of the CK2/PIAS1/SPOP axis induced by olaparib (Figures 4–6 and Supplemental Figures 3 and 6). In addition, CK2 has previously been reported to promote DDR by facilitating HR (31) without an underlying mechanism. Herein, we provide a potential explanation for CK2-mediated phosphorylation of PIAS1-S468, which increases its interaction with SPOP in olaparib-induced DNA damage. Subsequent PIAS1-induced SUMOylation of SPOP promoted its affinity for different substrates of SPOP during DNA damage (Supplemental Figure 5B). Although SPOP has been identified as a tumor suppressor in PCa, some clinical retrospective studies have found that PCa patients with SPOP mutations have a better prognosis than those with SPOP-WT (10, 32). Consistently, we found that PCa cells, animal models, and 3DP-POs with SPOP-WT were insensitive to PARP inhibitors, which may be due to the activation of DDR through the CK2/PIAS1/SPOP signaling axis. Intriguingly, we artificially induced “synthetic lethality” by disrupting this signaling axis through a CK2 inhibitor (silmitasertib) or SPOP inhibitor (SPOP-IN-6b), finally enhancing the therapeutic efficacy of PARP inhibitors in patients with PCa. Silmitasertib is currently approved by the FDA for the treatment of cholangiocarcinoma (22, 33), and the use of SPOP-IN-6b in renal cancer has been tested at the preclinical study stage (26). Notably, a recent study revealed that CK2 inhibitors disrupt CK2-mediated phosphorylation of CBX3, thereby inhibiting the subsequent ubiquitination and degradation of CBX3 and ultimately enhancing the efficacy of PARP inhibitors in PCa treatment (34). CBX3 participates in chromatin assembly and stability regulation to maintain genomic stability and influences the expression of target genes through transcriptional regulation (35). Therefore, the expression levels of CBX3 in PCa may also predict the response to PARP inhibitors. Although the signaling pathways targeted in our study may vary, similar conclusions demonstrate the feasibility of artificially inducing “synthetic lethality.” Besides the changes in cell apoptosis induced by DNA damage, we also sought out to test whether SPOP-3KR affects other functions, including cell proliferation and migration in PCa cells. Therefore, we performed PIAS1-WT/M3 and/or SPOP-WT/3KR overexpression in PCa cells with *PIAS1* knockdown to detect the changes in these processes. Intriguingly, there was no significant change in cell proliferation and migration, further confirming the specific role of SUMOylation of SPOP in DDR but not in other processes (Supplemental Figure 9).

*SPOP* mutations often lead to the hyperactivation of the AR signaling pathway, but *SPOP*-mutated PCa are also likely to develop into CRPC (36). Thus, we also investigated the role of the CK2/PIAS1/SPOP axis in response to olaparib-induced DNA damage in AR-positive PCa cells (LNCaP). These results demonstrated that the disruption of the CK2/PIAS1/SPOP axis also contributes to delay in DDR of LNCaP (Supplemental Figure 10). However, the relatively moderate change in the IC_50_ value in AR-positive cells (Figure 1B) suggests that the residual presence of AR may have some counteractive effect. In line with our results, several relevant clinical trials (ClinicalTrials.gov NCT01972217, NCT03395197, and NCT03732820) have shown increased efficacy in the combination of abiraterone/enzalutamide and PARP inhibitors. Considering the sensitivity of *SPOP*-mutated PCa to abiraterone, the combination could be an effective strategy to further enhance the sensitivity of *SPOP*-mutant PCa to olaparib (37).

PIAS1 has been confirmed as a critical factor in DDR in previous studies (38–40), suggesting that its function may be relevant to the response to PARP inhibitors; however, its role in PCa remains controversial. Some studies have indicated that PIAS1 can stabilize AR expression, potentially acting as an oncogenic factor in PCa (41), while other studies have shown that PIAS1 mediates zinc-induced apoptosis in PCa cells, suggesting its role as a drug-sensitive factor (42). However, specific small-molecule inhibitors targeting PIAS1 have not been identified.

In addition, some studies have suggested that the accumulation of cytoplasmic DNA due to DNA damage caused by PARP inhibitors can activate the innate immune cGAS/STING signaling pathway, suggesting that combinations of PARP inhibitors with immunotherapy may yield better results (43–45). In addition, *SPOP*-mutated PCa exhibits oncogenic activation of the STING/NF-κB signaling pathway. The use of PARP inhibitors can shift this to the anticancer cGAS/STING signaling pathway, thereby further supporting the application of PARP inhibitors in *SPOP*-mutated PCa (10, 29).

In summary, our study is the first to our knowledge to reveal the critical role of CK2/PIAS1/SPOP in the DDR. PCa-associated SPOP mutants evade PIAS1-mediated SUMOylation, impairing DDR, promoting apoptosis, and increasing sensitivity to PARP inhibitors. Furthermore, we demonstrated the potential of a CK2 inhibitor and SPOP inhibitor to enhance the efficacy of PARP inhibitors in PCa with SPOP-WT.

## Methods

### Sex as a biological variable.

Our study exclusively examined male mice because the disease modeled (PCa) is only relevant in males.

### Animal experiments.

For animal experiments, 4-week-old BALB/c nude mice were obtained from Jiangsu GemPharmatech Co., Ltd., bred in-house, and used for animal experiments. All mice were housed under standard conditions with a 12-hour light/12-hour dark cycle and had ad libitum access to food and water. Each group of PC-3 cells was inoculated into the right flank of BALB/c nude mice (1 × 10^6^ cells per mouse), and treated with vehicle or drugs on the 17th day after PC-3 cell inoculation. Tumor growth was measured every 4 days. After the experiments, the mice were euthanized to obtain the tumor tissues. Tumors on day 30 were harvested and photographed.

### Cell culture.

HEK-293T (TCH-C101), PC-3 (TCH-C297), DU145 (TCH-C170), and human osteosarcoma (U-2OS; TCH-C365) cell lines were obtained from Haixing Biosciences. HEK-293T cells were cultured in Dulbecco’s modified Eagle medium (DMEM; Pricella, PM150210) supplemented with 10% fetal bovine serum (FBS; Vazyme, F101-01). The PC-3 cell line was cultured in Ham’s F-12K (Pricella, PM150910) with 10% FBS. DU145 cells were cultured in Minimum Essential Medium (MEM, Pricella, PM150410) supplemented with 10% FBS. The U-2OS cell line was cultured in McCoy’s 5A (Pricella, PM150710) with 10% FBS. All the cells were grown at 37°C with 5% CO_2_.

### Plasmids and mutagenesis.

Expression vectors for SPOP-WT and its mutants have been previously described (7). The FLAG- and Myc-PIAS1-WT constructs were obtained from the MiaoLing Plasmid Platform (MiaoLing Biology). SPOP-L90V/K101I and PIAS1 mutants were generated using the Mut Express II Fast Mutagenesis Kit V2 (Vazyme, C214) and specific primers (Supplemental Table 2).

### Flow cytometry.

Flow cytometry was performed using an Annexin V-FITC/PI apoptosis kit (MULTI SCIENCES, AT101), according to the manufacturer’s instructions.

### Transfection and virus infection.

All transfection experiments were performed using Lipo6000 transfection reagent (Beyotime, C0526) and Lipo8000 transfection reagent (Beyotime, C0533), according to the manufacturer’s instructions. The shRNA constructs and virus packing constructs were transfected into HEK-293T cells, and the virus supernatant was collected 48 hours after transfection. The indicated cancer cells were infected with virus-containing supernatants in the presence of polybrene (8 μg/mL) and then selected in growth medium containing puromycin (1.5 μg/mL). Total shRNA and siRNA sequences are listed in Supplemental Table 2. All constructs were verified by DNA sequencing.

### GST pull-down assays.

GST fusion proteins were obtained from *E*. *coli* BL21 (DE3) competent cells and purified using a GST-tag Protein Purification Kit (Beyotime, P2262). GST fusion proteins were immobilized on glutathione-Sepharose beads. After washing with pull-down buffer (20 mM Tris-HCl pH 7.5, 150 mM NaCl, 0.1% NP-40, 1 mM DTT, 10% glycerol, 1 mM EDTA, 2.5 mM MgCl_2_, and 1 μg/mL leupeptin), the beads were incubated with cell lysates for 2 hours. The beads were then washed 5 times with binding buffer and resuspended in sample buffer. The bound proteins were resolved by SDS-PAGE and analyzed by Western blotting and Coomassie blue staining.

### In vivo SUMOylation assay.

Cells were transfected with the indicated plasmids. Twenty-four hours after transfection, the cells were treated with 20 μM MG132 (Selleck, S2619) for 8 hours and lysed with IP buffer on ice for more than 10 minutes. The lysate was sonicated and centrifuged for 15 minutes at 12,000*g* and 4°C, and the supernatant was incubated with anti-FLAG antibody–conjugated Magnetic Beads (Bimake, B26101) with rotation at 4°C overnight. The next day, the beads were washed extensively with IP buffer and the proteins were extracted by boiling at 95°C for 5 minutes. Finally, the proteins were resolved by SDS-PAGE for Western blotting and stained with anti-FLAG, -SUMO1, and -SUMO2/3 antibodies.

### In vitro SUMOylation assay.

In vitro SUMOylation assays were performed using recombinant GST-SPOP, His-PIAS1, E1, E2, and SUMO1 proteins. The assays were performed in reaction buffer (50 mM Tris-HCl pH 7.5, 5 mM MgCl_2_, 2 mM ATP, 2 mM DTT, and 100 mM NaCl) and incubated at 37°C for 1 hour. The reaction products were analyzed using SDS-PAGE and Western blotting.

### In vivo ubiquitination assay.

Cells were transfected with HA-ubiquitin and the indicated plasmids. Twenty-four hours after transfection, the cells were treated with 20 μM MG132 for 8 hours and lysed with IP buffer on ice for more than 10 minutes. The lysate was sonicated and centrifuged for 15 minutes at 12,000*g* and 4°C, and the supernatant was incubated with anti-FLAG–conjugated Magnetic Beads (Bimake, B26101) with rotation at 4°C overnight. The next day, the beads were washed extensively with IP buffer and the proteins were extracted by boiling at 95°C for 5 minutes. Lastly, the proteins were resolved by SDS-PAGE for Western blotting.

### In vitro kinase activity assays.

In vitro kinase activity assays were performed using a Luminescent Kinase Assay Kit (Beyotime, S0158), according to the manufacturer’s instructions.

### Immunofluorescence and confocal microscopy.

For immunofluorescence, the cells were plated on chamber slides and fixed with 4% paraformaldehyde at room temperature for 30 minutes. After washing with PBS, cells were permeabilized with 0.1% Triton X-100 in PBS for 15 minutes. The cells were then washed with PBS, blocked with 2% BSA in PBS for 1 hour, and incubated with primary antibodies in PBS at 4°C overnight. After washing with PBS, fluorophore-labeled secondary antibodies were applied and cells were counterstained with DAPI for 1 hour at room temperature. Cells were visualized and imaged using a confocal microscope (Leica TCS SP8 STED).

DNA damage foci (γH2AX, BRCA1, and Ku70) were counted manually or quantified using Image-Pro Plus 6.0 software (Media Cybernetics). For each cell line analyzed using Image-Pro, 5 randomly selected photographs that included more than 100 cells were used to standardize foci counting and integration of the optical density.

TUNEL staining was performed using the TUNEL Apoptosis Kit (UElandy, T603) according to the manufacturer’s instructions and imaged using a confocal microscope (Leica TCS SP8 STED).

### Comet assays.

Comet assays were performed using the Comet Assay Kit (Beyotime, C0533), according to the manufacturer’s instructions and imaged using a confocal microscope (Leica TCS SP8 STED). The comet assays were then performed, and 10 cells from each sample were analyzed based on the tail moment using the Komet 3.1 Image Analysis System (Kinetic Imaging).

### Cell viability assay.

The IC_50_ value of olaparib in PCa cells was determined using a cell viability assay. Cells (5000 cells per well) under each condition were plated in 96-well plates and cultured in 100 μL of the indicated medium containing 10% serum. After 24 hours, the cells were treated with various concentrations of compounds in 100 μL of medium or left untreated for 24–48 hours, and cell viability was measured using the Cell Counting Kit-8 (CCK-8) (APExBIO, K1018), according to the manufacturer’s instructions. Absorbance was measured at 450 nm using a microplate absorbance reader (Bio-Rad). All cell viability experiments were conducted in triplicate and the average value represents the value of a single biological replicate.

### 3DP-POs and CellTiter-Glo 3D cell viability assay.

The development of 3D bioprinted primary PCa models and conducting in vitro drug sensitivity tests were supported by ChangDu BioEng. & Therapy Co.,Ltd

### Data acquisition.

*PIAS1* mRNA levels in PCa were obtained from TCGA. Further analyses, including differential expression analyses, were performed using R software (version 4.2.1; https://www.r-project.org/).

### Proteomics and bioinformatics.

Proteomics services were provided by Shanghai Bioprofile and OE Biotech. Proteomics data were processed using volcano plots, Venn diagrams, and heatmaps using R software (version 4.2.1). Relevant data can be obtained from Supporting Data Values file in the supplemental material.

### Statistics.

Statistical calculations were performed using GraphPad Prism software. Data are presented as mean ± SD for experiments performed with at least 3 replicates. The differences between 2 groups were analyzed using Student’s *t* test, and multiple comparisons were performed using 2-way analysis of variance (ANOVA). The χ^2^ test was used for statistical analysis of categorical data. A *P* value of less than 0.05 was considered significant.

### Study approval.

The study protocol for clinical samples of PCa tissues and the construction of 3DP-POs from patients were approved by the Institutional Ethics Committee of Ningbo University (NBU-2024-125). All the patients provided written informed consent to participate in the study. Animal experiments were approved by the Institutional Animal Care and Use Committee of the Ningbo University (NBU-20220202).

### Data availability.

All data are available in the full unedited blot/gel images and the Supporting Data Values file.

## Author contributions

HZ, LK, JL, ZL, YZ, XL, LW, LC, HY, JJ, XC, ZZ, and YL developed methodology, conducted experiments, and generated figures. HZ wrote the manuscript. XJ and ZY supervised the study and reviewed and edited the manuscript.

## Supplementary Material

Supplemental data

Unedited blot and gel images

Supporting data values

## Figures and Tables

**Table 1:**
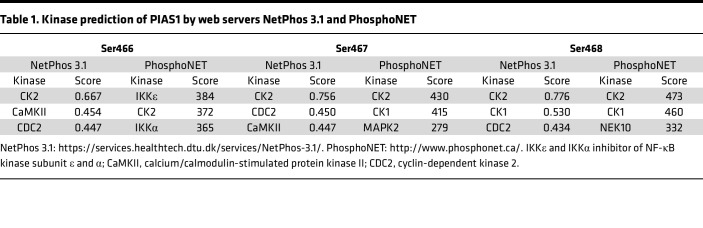
Kinase prediction of PIAS1 by web servers NetPhos 3.1 and PhosphoNET

**Table 2:**
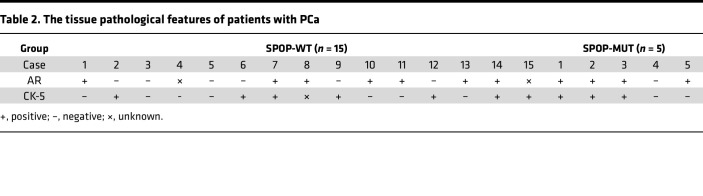
The tissue pathological features of patients with PCa

## Version 1 04/22/2025

Electronic publication

## Version 2 04/24/2025

The Acknowledgments and References were updated.
