# Supplementary material for: SPOP mutations increase PARP inhibitor sensitivity via CK2/PIAS1/SPOP axis in prostate cancer
Source: JCI Insight. 2025 Apr 22;10(8):e186871. doi: 10.1172/jci.insight.186871 (PMC12016936; doi:10.1172/jci.insight.186871)
Supplement: Supplemental data [file jciinsight-10-186871-s013.pdf]

Supplementary materials for

SPOP mutations increase PARP inhibitor sensitivity via CK2-PIAS1-SPOP axis in prostate cancer

Hui Zhang *et.al*

Corresponding author: jinxiaofeng@nbu.edu.cn (X.J.); fyyyanzejun@nbu.edu.cn (Z.Y.)

The PDF file includes:

Supplementary Figure 1 to 10

Supplementary Table 1 to 2

Other Supplementary Material for this manuscript includes the following:

Supporting data values and full unedited blot/gel images

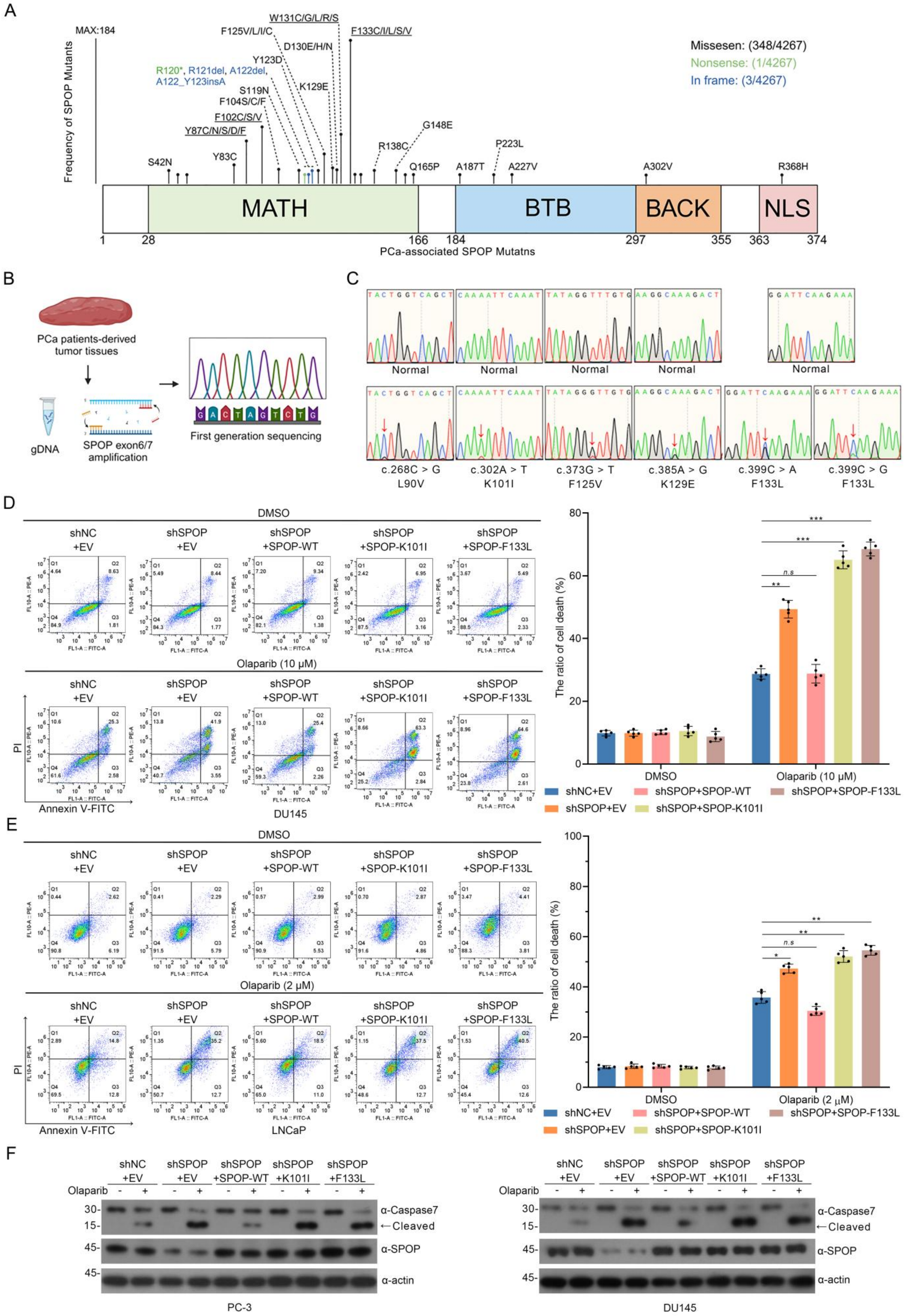

A
MAX:184
Frequency of SPOP Mutants
Missesen: (348/4267)
Nonsense: (1/4267)
In frame: (3/4267)
S42N
Y83C
Y87C/N/S/D/F
F102C/S/V
F104S/C/F
S119N
R120*, R121del, A122del, A122_Y123insA
Y123D
F125V/L/I/C
K129E
D130E/H/N
W131C/G/L/R/S
F133C/I/L/S/V
R138C
G148E
Q165P
A187T
P223L
A227V
A302V
R368H
MATH
BTB
BACK
NLS
1 28 166 184 297 355 363 374
PCa-associated SPOP Mutatns
B
PCa patients-derived tumor tissues
gDNA
SPOP exon6/7 amplification
First generation sequencing
C
Normal
c.268C > G L90V
c.302A > T K101I
c.373G > T F125V
c.385A > G K129E
c.399C > A F133L
c.399C > G F133L
D
DMSO
Olaparib (10 μM)
shNC +EV
shSPOP +EV
shSPOP +SPOP-WT
shSPOP +SPOP-K101I
shSPOP +SPOP-F133L
PI
Annexin V-FITC
DU145
The ratio of cell death (%)
shNC+EV
shSPOP+EV
shSPOP+SPOP-WT
shSPOP+SPOP-K101I
shSPOP+SPOP-F133L
E
DMSO
Olaparib (2 μM)
LNCaP
F
Olaparib
α-Caspase7
←Cleaved
α-SPOP
α-actin
PC-3
DU145

Supplementary Figure 1. The high-frequency mutations of *SPOP* in PCa lead to increased sensitivity of PCa cells to Olaparib-induced apoptosis. (A) *SPOP* mutations occur in 10-15% of PCa, and these mutations are predominantly located within the MATH domain. **(B)** Flowchart for identification of *SPOP* gene mutations in PCa patients. **(C)** In the collected 50 cases of PCa tissues, 6 cases were detected with missense mutations in the *SPOP* gene. **(D)** Representative images (left) and statistical graph (right) of flow cytometry showing the apoptosis levels of DU145 cells in each group induced by Olaparib (10 μM). **(E)** Representative images (left) and statistical graph (right, n = 5) of flow cytometry showing the apoptosis levels of LNCaP cells in each group induced by Olaparib (2 μM). **(F)** Western blotting of WCLs obtained from PC-3 (left) and DU145 (right) cells in each group under Olaparib (10 μM) treatment; staining with anti-Casepase-7 antibodies. Data were shown as the mean ± SD, two-tailed unpaired Student's t test. **P*<0.05, ***P*<0.01, ***P*<0.001.

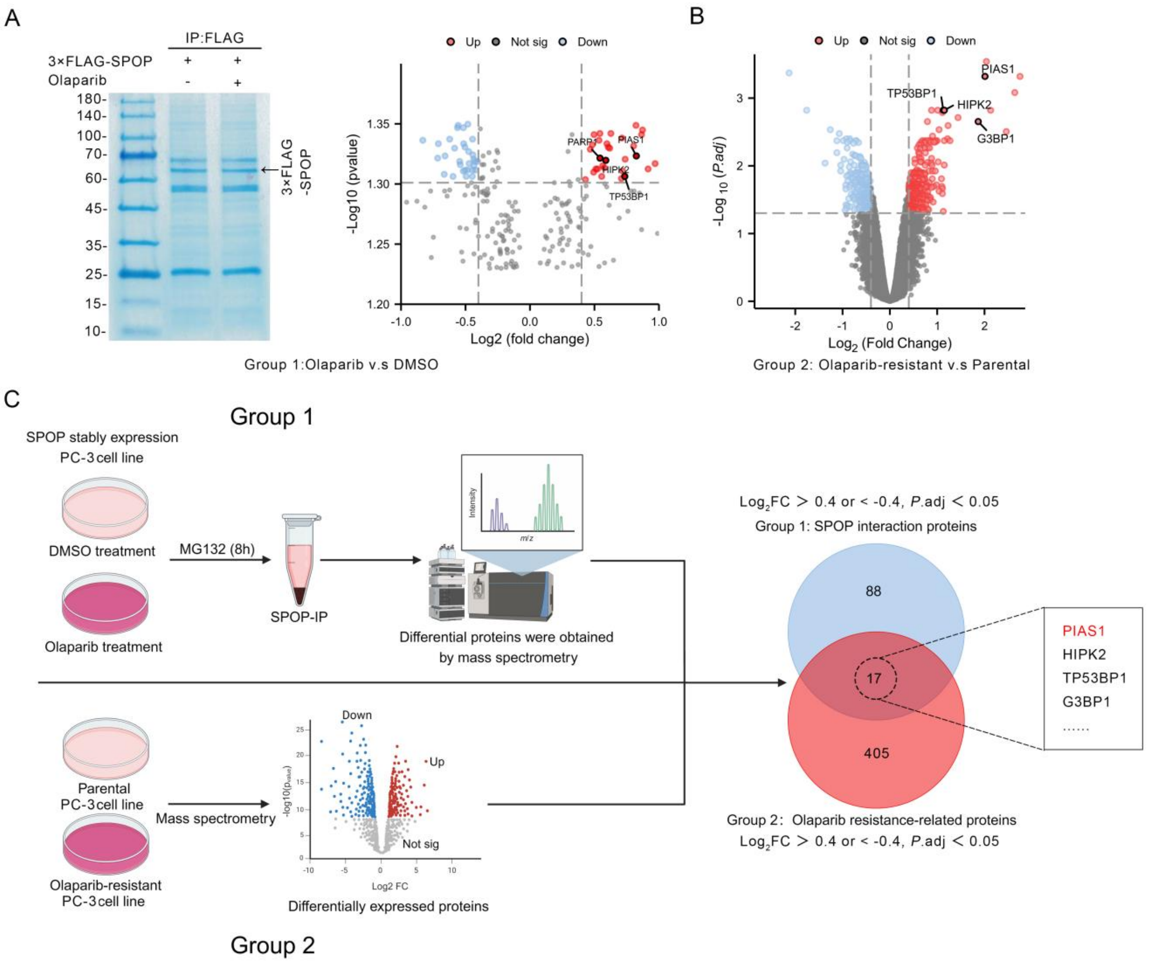

Supplementary Figure 2. Proteomic analysis reveals proteins potentially involved in SPOP-associated DDR and the response of SPOP to Olaparib-induced apoptosis. (A) 3×FLAG-SPOP protein complex are obtained from PC-3 cells under Olaparib (10 μM) treatment or not through co-IP of anti-FLAG antibody and detected by Coomassie Blue staining (left) and mass spectrometry (right). Using bioinformatics to generate volcano plots, proteins with significant changes in interaction with SPOP under Olaparib (10 μM) treatment were identified; Log2 (fold change) > 0.4 or < -0.4, -Log10 (p value) > 1.30. **(B)** Proteomic analysis of protein expression differences between Olaparib-resistant PC-3 cells and their corresponding parental cell lines was conducted, and Olaparib resistance-related proteins were identified by generating volcano plots; Log2 (fold change) > 1 or < -1, -Log10 (p value) > 1.30. **(C)** The specific workflow diagrams for **A** and **B**, as well as the VENN diagram identifying proteins with significant differential changes identified in **A** and **B**.

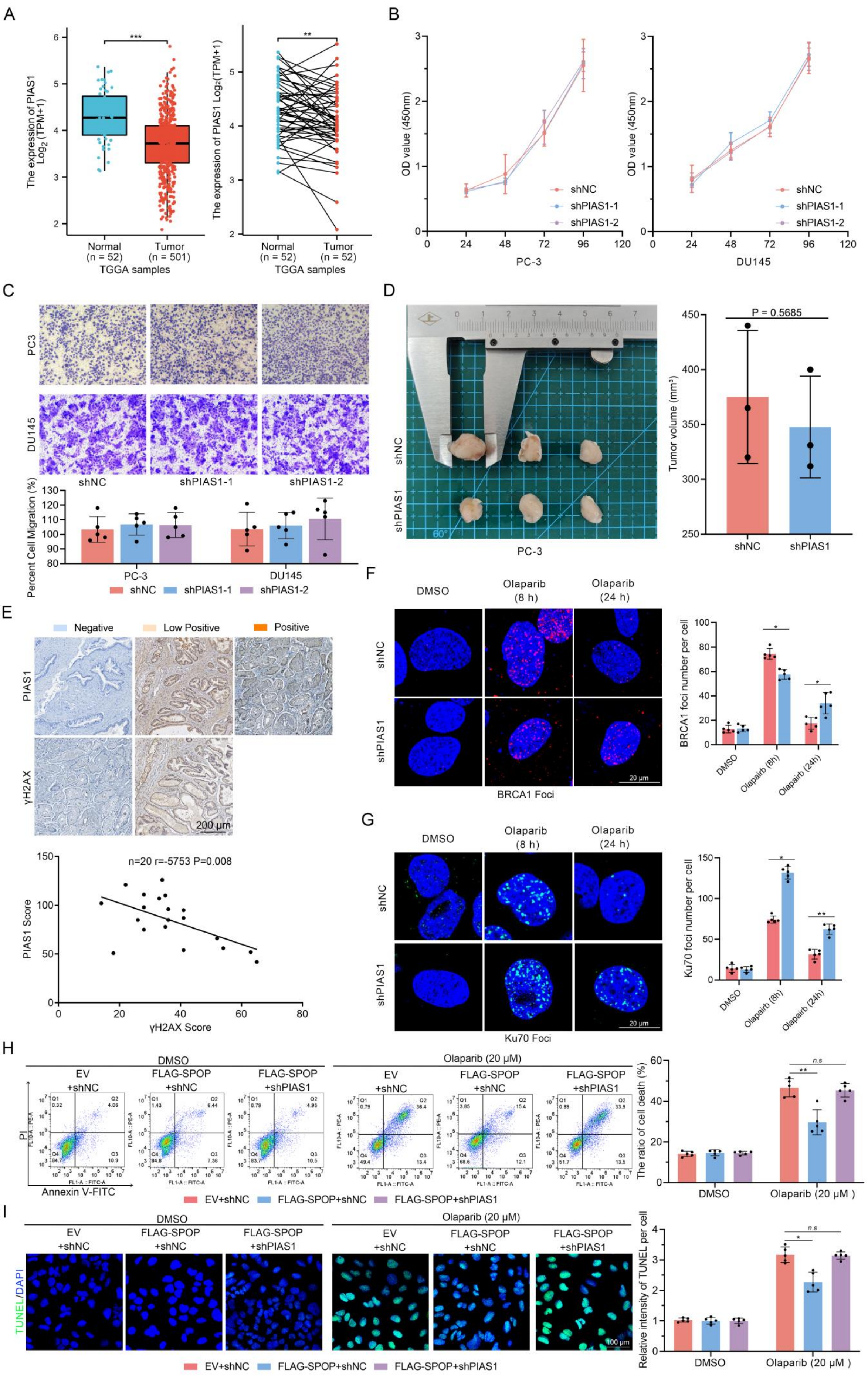
A
The expression of PIAS1 Log2(TPM+1)
Normal (n = 52)
Tumor (n = 501)
TGGA samples
Normal (n = 52)
Tumor (n = 52)
B
OD value (450nm)
shNC
shPIAS1-1
shPIAS1-2
PC-3
DU145
C
PC3
DU145
shNC
shPIAS1-1
shPIAS1-2
Percent Cell Migration (%)
PC-3
DU145
D
shNC
shPIAS1
PC-3
P = 0.5685
Tumor volume (mm³)
E
Negative
Low Positive
Positive
PIAS1
γH2AX
200 μm
n=20 r=-5753 P=0.008
PIAS1 Score
γH2AX Score
F
DMSO
Olaparib (8 h)
Olaparib (24 h)
BRCA1 Foci
20 μm
BRCA1 foci number per cell
G
Ku70 Foci
Ku70 foci number per cell
H
DMSO
Olaparib (20 μM)
EV +shNC
FLAG-SPOP +shNC
FLAG-SPOP +shPIAS1
PI
Annexin V-FITC
The ratio of cell death (%)
EV+shNC
FLAG-SPOP+shNC
FLAG-SPOP+shPIAS1
I
TUNEL/DAPI
100 μm
Relative intensity of TUNEL per cell

Supplementary Figure 3. PIAS1 does not affect the proliferation, migration, and tumor formation of PCa cells but is involved in maintaining genomic stability. (A) The TCGA database indicates a significant decrease in *PIAS1* mRNA levels in PCa compared to normal tissue. **(B)** The CCK-8 assay demonstrates that PIAS1 does not affect the proliferation of PC-3 and DU145 cells. **(C)** The migration assay (up) and corresponding statistical graph (down) reveals that PIAS1 does not affect the migration of PC-3 and DU145 cells. **(D)** The CDX models (left) and corresponding statistical graph (right) reveals that PIAS1 does not affect the tumor formation of PC-3 cells. **(E)** Representative images of IHC staining for γH2AX and PIAS1 (up) in PCa tissues, along with the corresponding statistical analysis of staining intensity (down); n = 20, r = -0.5753, p = 0.008. **(F)** BRCA1 foci (left) were stained and corresponding statistical graph (right) in PC-3 cells; **(G)** Ku70 foci (left) were stained and corresponding statistical graph (right) in PC-3 cells. **(H)** Representative images (left) and statistical graph (right, n = 5) of flow cytometry and TUNEL staining showing the apoptosis levels of PC-3 cells in each group under Olaparib (20 μM) treatment. **(I)** Representative images (left) and statistical graph (right, n = 5) of TUNEL staining showing the apoptosis levels of PC-3 cells in each group under Olaparib (20 μM) treatment. Data were shown as the mean ± SD, two-tailed unpaired Student's t test. *P<0.05, **P<0.01, **P<0.001.

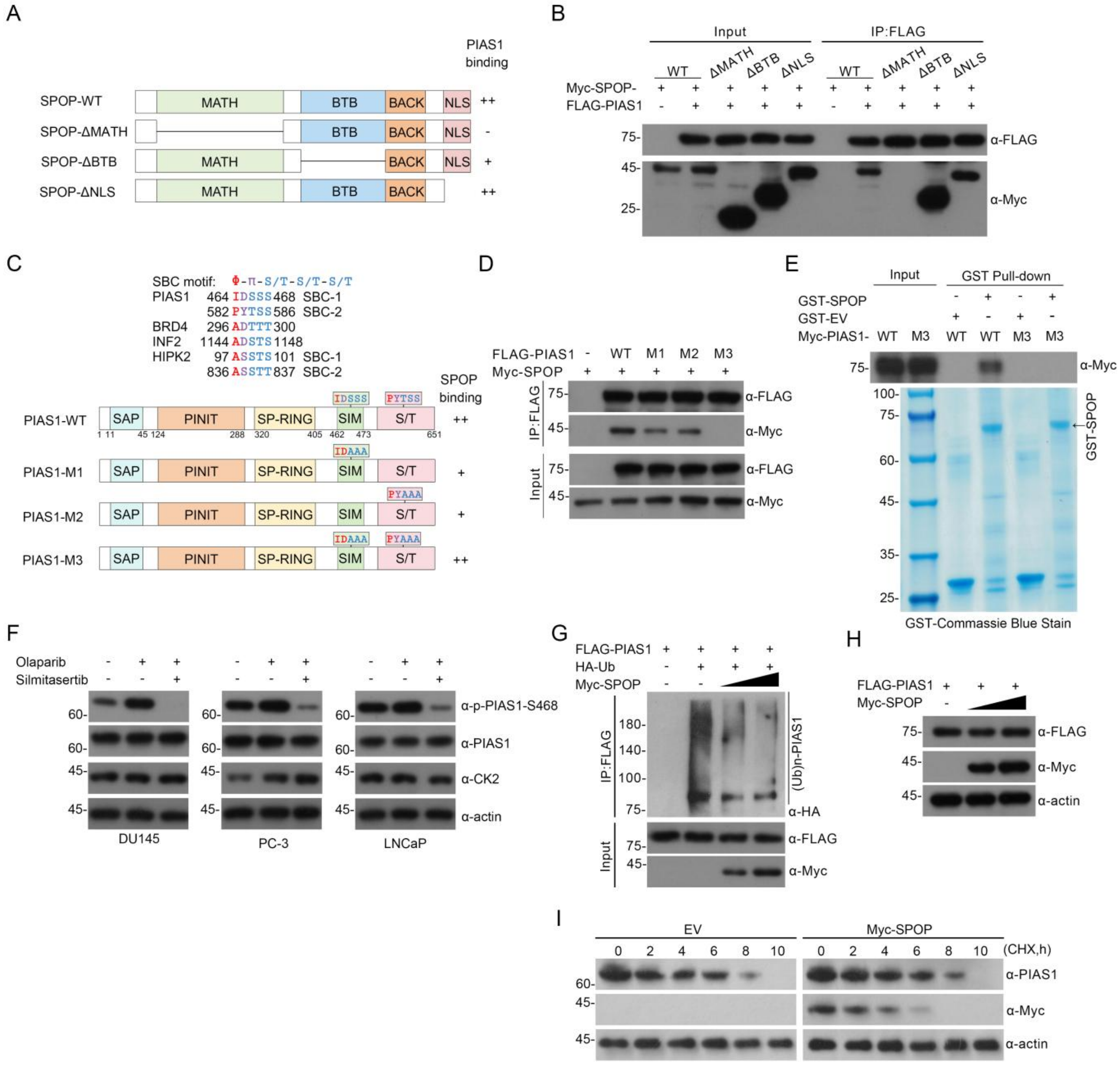

Supplementary Figure 4. Identification of the domains/motifs involved in the interaction between PIAS1 and SPOP. (A) Diagram showed SPOP-WT/ΔMATH/ΔBTB/ΔNLS; **(B)** Western blotting of the indicated proteins in WCL and co-IP samples of anti-FLAG antibody obtained from HEK-293T cells transfected with indicated plasmids (right). **(C)** Diagram showed the potential SBC motif on PIAS1 and its corresponding mutants. **(D)** Western blot of the indicated proteins in WCL and co-IP samples of anti-FLAG antibody obtained from HEK-293T cells transfected with indicated plasmids. **(E)** *in vitro* (GST-SPOP pull-down) interaction assays from HEK-293T cells showed the impaired interaction of Myc-PIAS1-M3 and SPOP. **(F)** Western Blotting utilizing cell lysates from DU145/PC-3/LNCaP cells treated with Olaparib/Silmitasertib or not. **(G)** *In vivo* ubiquitination assay showed that SPOP did not mediate the ubiquitination of PIAS1. **(H)** Western blotting showed that SPOP did not affect the protein levels of PIAS1. **(I)** Western blotting showed that SPOP did not affect the protein degradation half-life of PIAS1.

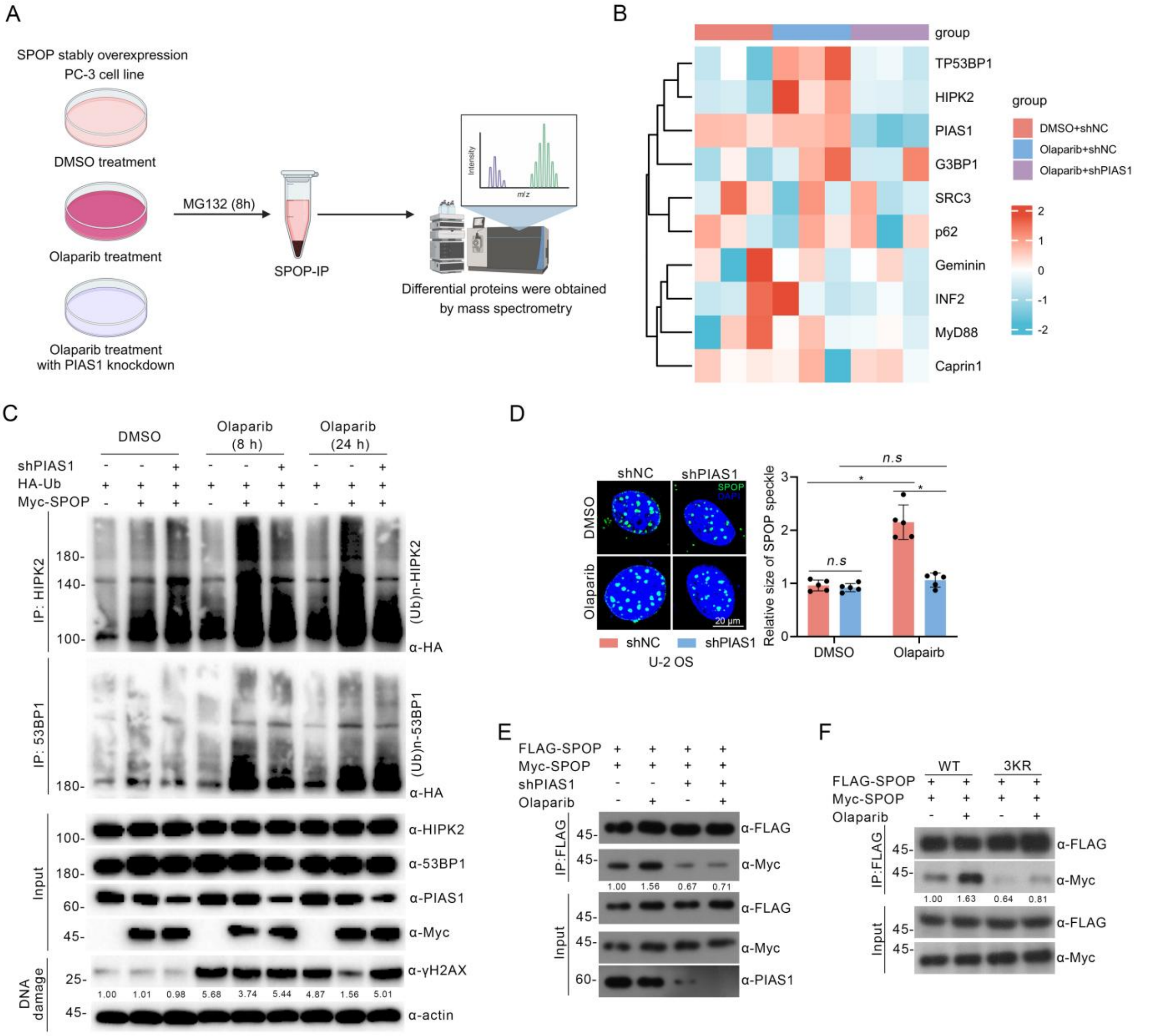

Supplementary Figure 5. PIAS1-mediated SUMOylation of SPOP may influence SPOP-associated DDR. **(A)** The specific workflow diagrams of identification for SPOP-interacting substrates under the Olaparib treatment and *PIAS1* knockdown. **(B)** The heatmap shows changes in the interaction between SPOP and previously reported substrates fFollowing treatment with Olaparib and *PIAS1* knockdown. **(C)** *in vivo* ubiquitination assays showed the SPOP-mediated ubiquitination of HIPK2 and 53BP1 under Olaparib treatment through using lysate from HEK-293T cells transfected with Myc-SPOP and shPIAS1 plasmids. Western blotting and quantification of γH2AX showed the DDR process of HEK-293T cells. **(D)** SPOP speckle were stained (left) and statistical graph (right) of speckle size. **(E)**co-IP utilizing cell lysates from FLAG-SPOP-WT/3KR and Myc-PIAS1 transfected HEK-293T cells treated with Olaparib or not, staining with FLAG and Myc antibodies. **(F)** co-IP utilizing cell lysates from FLAG-SPOP-WT/3KR and Myc-SPOP transfected HEK-293T cells treated with Olaparib or not, staining with FLAG and Myc antibodie. Data were shown as the mean ± SD, two-tailed unpaired Student's t test. *P<0.05.

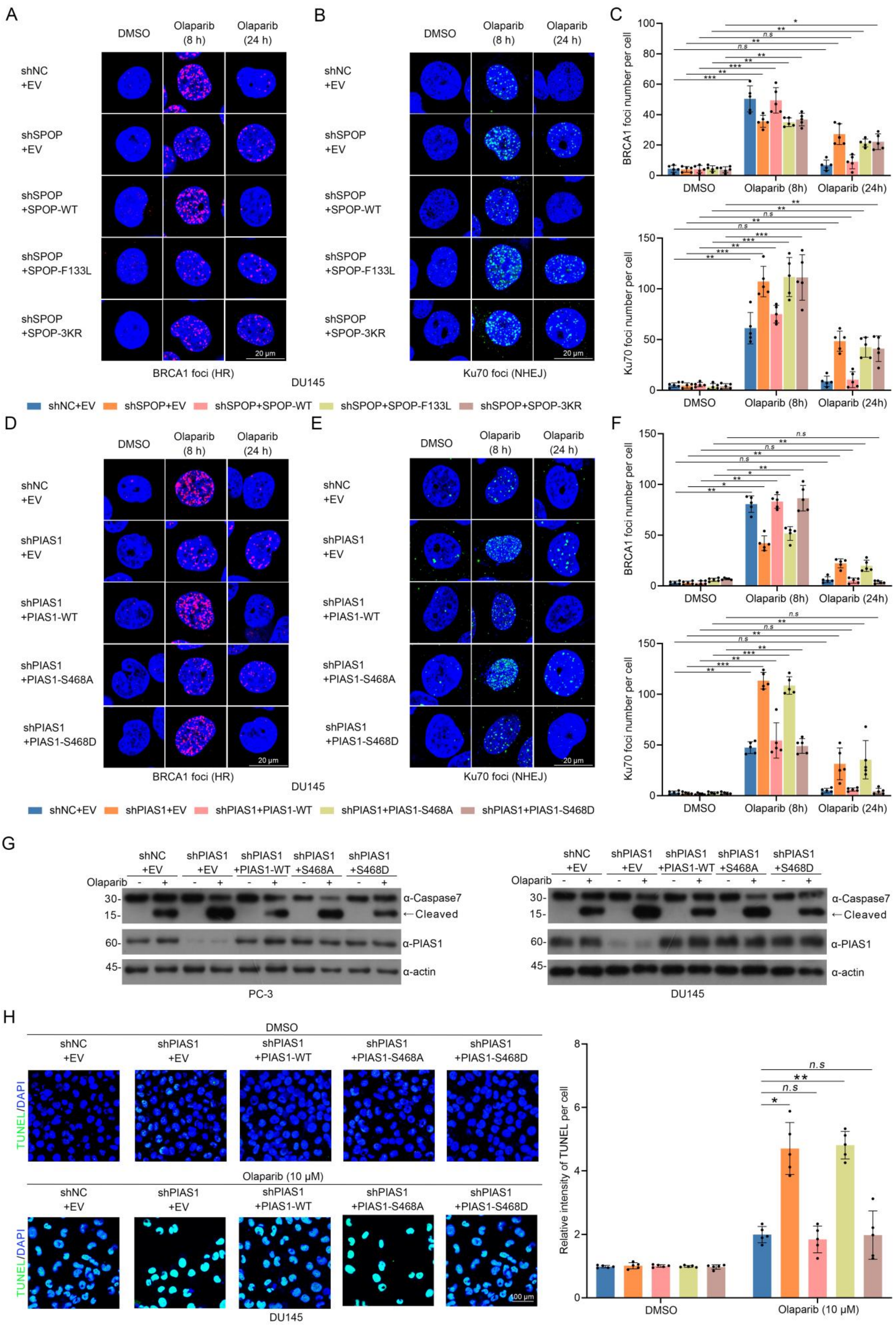
A
DMSO
Olaparib (8 h)
Olaparib (24 h)
shNC +EV
shSPOP +EV
shSPOP +SPOP-WT
shSPOP +SPOP-F133L
shSPOP +SPOP-3KR
20 μm
BRCA1 foci (HR)
B
Ku70 foci (NHEJ)
DU145
C
BRCA1 foci number per cell
Ku70 foci number per cell
DMSO
Olaparib (8h)
Olaparib (24h)
shNC+EV
shSPOP+EV
shSPOP+SPOP-WT
shSPOP+SPOP-F133L
shSPOP+SPOP-3KR
D
E
F
shPIAS1 +EV
shPIAS1 +PIAS1-WT
shPIAS1 +PIAS1-S468A
shPIAS1 +PIAS1-S468D
shNC+EV
shPIAS1+EV
shPIAS1+PIAS1-WT
shPIAS1+PIAS1-S468A
shPIAS1+PIAS1-S468D
G
Olaparib
α-Caspase7
←Cleaved
α-PIAS1
α-actin
PC-3
DU145
H
TUNEL/DAPI
Olaparib (10 μM)
100 μm
Relative intensity of TUNEL per cell
DMSO
Olaparib (10 μM)

Supplementary Figure 6. Deregulation of CK2-PIAS1-SPOP axis leads to impaired DDR, and sensitivity to Olaparib-induced apoptosis in PCa cells. (A) BRCA1 foci were stained under Olaparib (10 μM) treatment to determine the HR process in DU145 cells. **(B)** Ku70 foci were stained under Olaparib (10 μM) treatment to determine the NHEJ process in DU145 cells. **(C)** Corresponding statistical graph of BRCA1 foci (up, n = 5) in **(A)** and Ku70 foci (down, n = 5) in **(B)**. **(D)** BRCA1 foci were stained under Olaparib (10 μM) treatment to determine the HR process in DU145 cells. **(E)** Ku70 foci were stained under Olaparib (10 μM) treatment to determine the NHEJ process in DU145 cells. **(F)** Corresponding statistical graph of BRCA1 foci (up, n = 5) in **(D)** and Ku70 foci (down, n = 5) in **(E)**. **(G)** WCLs obtained from PC-3 (left) and DU145 (right) cells in each group under Olaparib (10 μM) treatment; staining with anti-Casepase-7 antibodies. **(H)** Representative images (left) and statistical graph (right, n = 5) of TUNEL staining showing the apoptosis levels of DU145 cells in each group under Olaparib (10 μM) treatment. Data were shown as the mean ± SD, two-tailed unpaired Student's t test. *P<0.05, **P<0.01, **P<0.001.

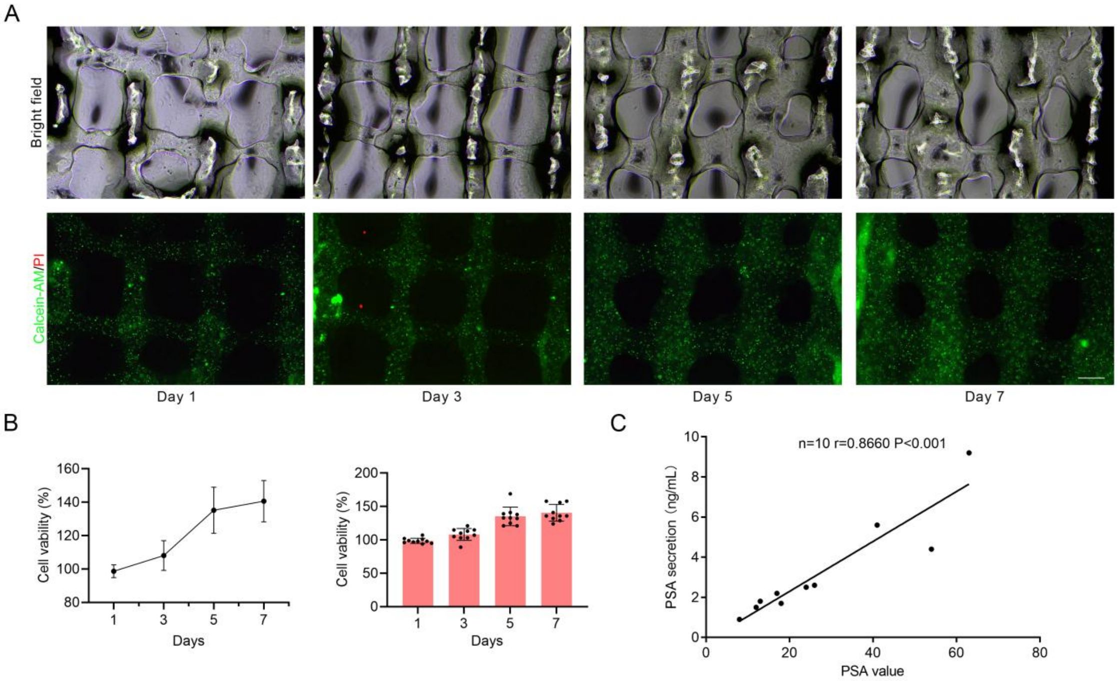

Supplementary Figure 7. Constructing 3D-POs. (A) Calcein-AM/PI staining of 3DP-POs after construction in day 1/3/5/7. **(B)** 3D cell viability assays of 3DP-POs after construction in day 1/3/5/7 (n = 10). **(C)** The correlation between PSA secretion from 3DP-POs and the PSA value obtained from patients clinical data.

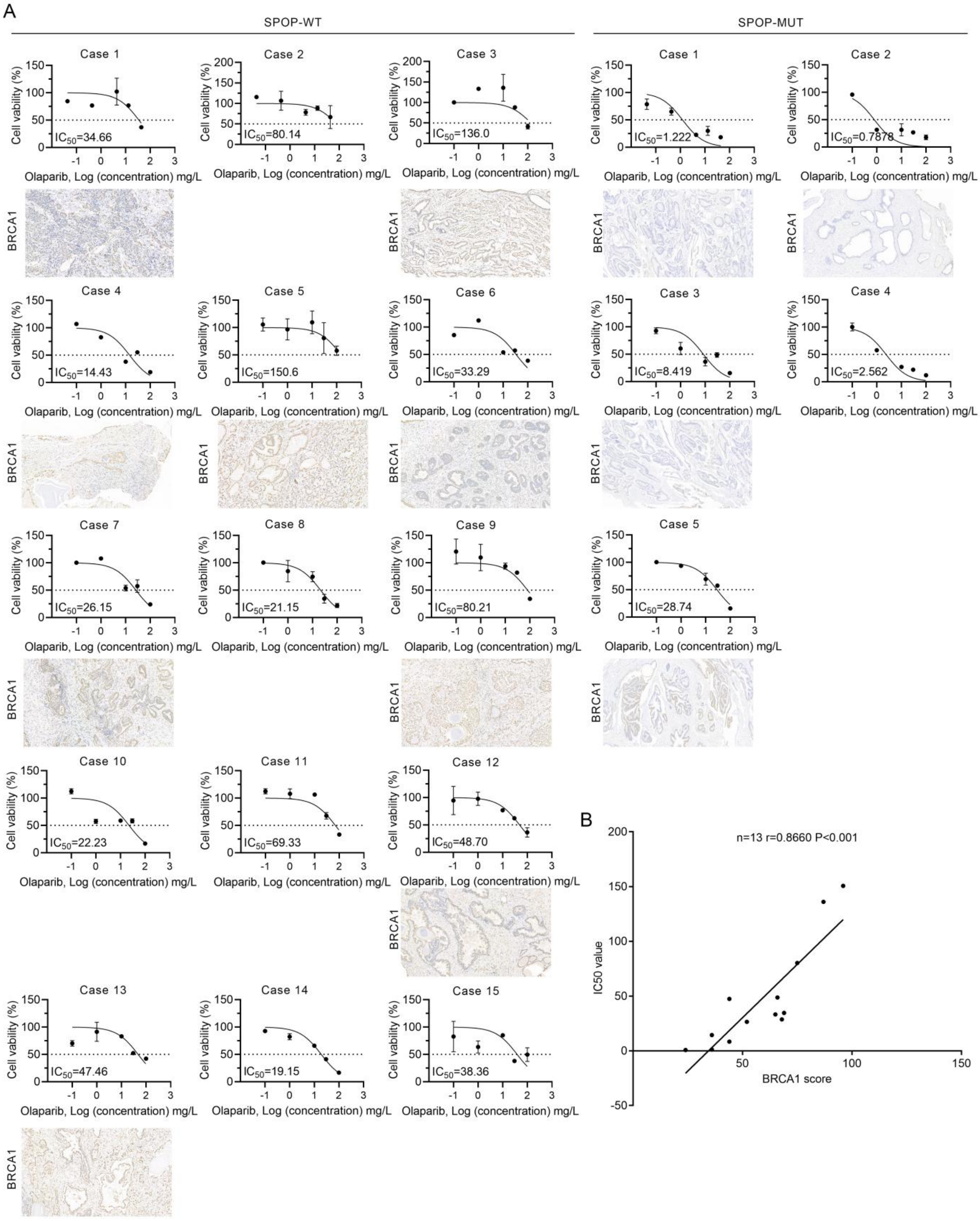

Supplementary Figure 8. **(A)** The 3D-POs were divided into two groups (SPOP-WT, n = 15; SPOP-MUT, n =5) based on the *SPOP* gene mutation; the IC50 value of each 3DP-POs to Olaparib in all cases were determined, and corresponding BRCA1 staining in partial cases were staining. **(B)** Correlation analysis between the BRCA1 staining and the IC50 values (n = 13, r=0.8660, P<0.001).

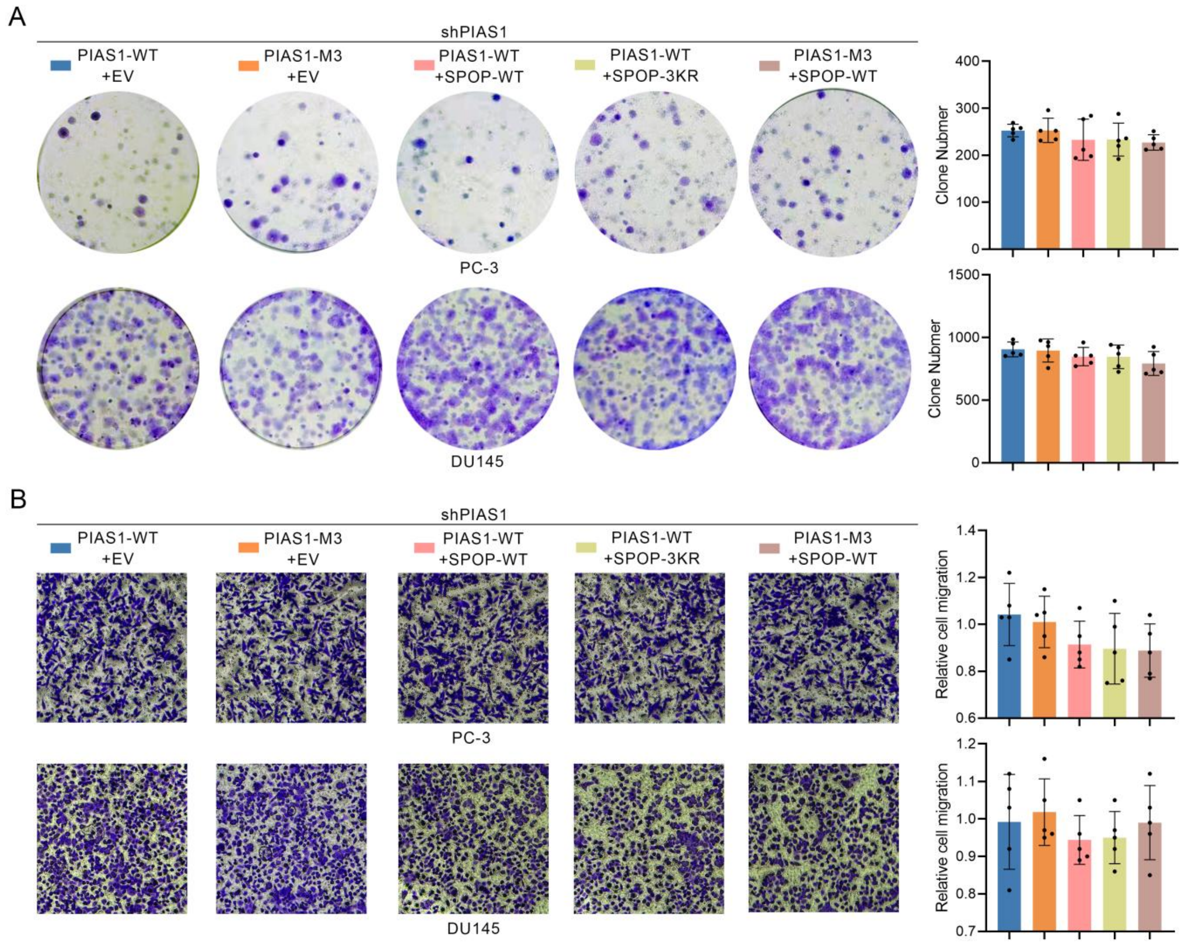

Supplementary Figure 9. (A) The colony formation (left) and corresponding statistical graph (right; n = 5) demonstrates proliferation ability of PC-3 and DU145 cells. **(B)** The migration assay (left) and corresponding statistical graph (right; n = 5) demonstrates migration ability of PC-3 and DU145 cells.

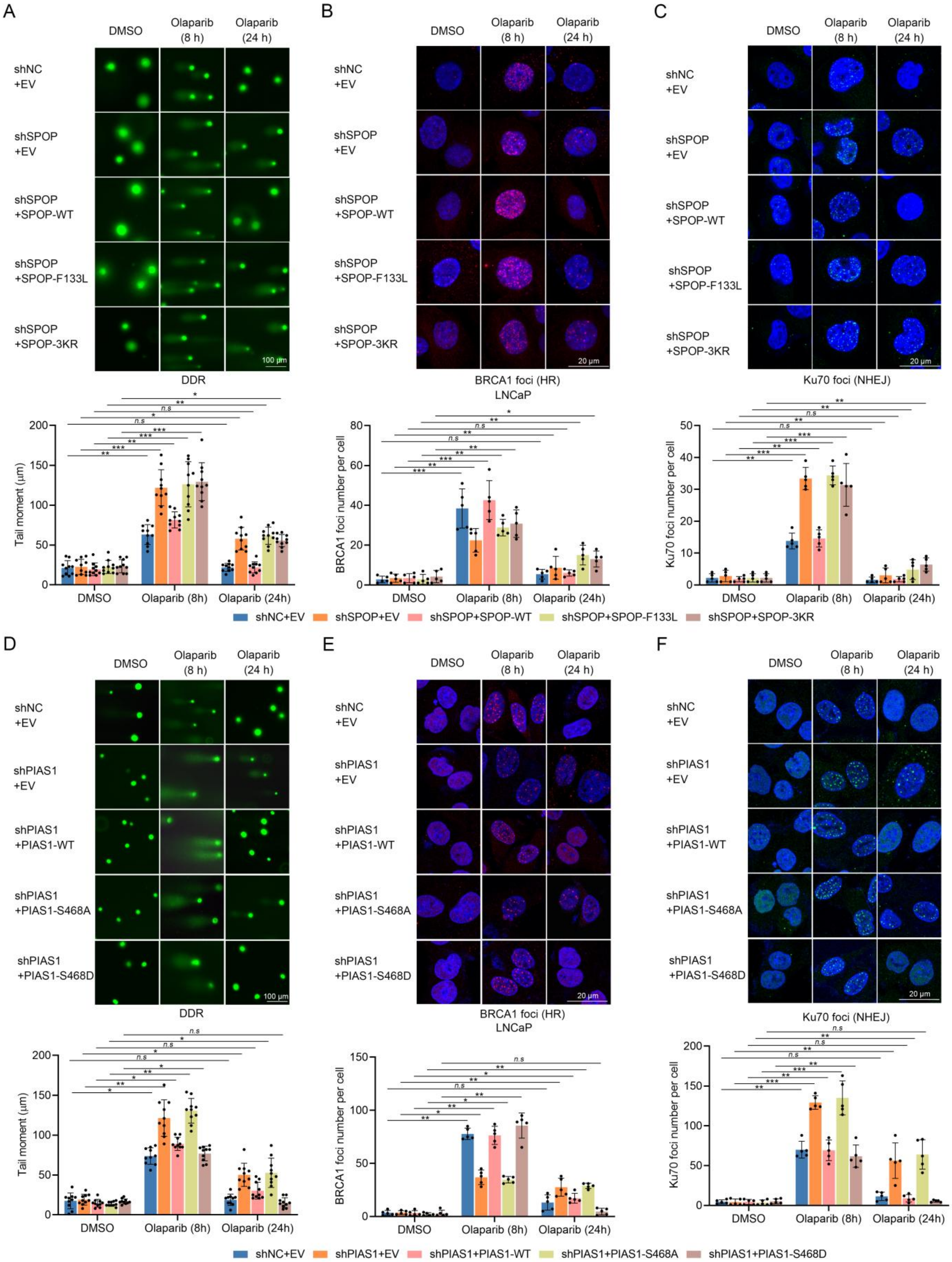
A
DMSO
Olaparib (8 h)
Olaparib (24 h)
shNC +EV
shSPOP +EV
shSPOP +SPOP-WT
shSPOP +SPOP-F133L
shSPOP +SPOP-3KR
100 μm
DDR
Tail moment (μm)
DMSO
Olaparib (8h)
Olaparib (24h)
B
BRCA1 foci (HR)
LNCaP
BRCA1 foci number per cell
20 μm
C
Ku70 foci (NHEJ)
Ku70 foci number per cell
shNC+EV
shSPOP+EV
shSPOP+SPOP-WT
shSPOP+SPOP-F133L
shSPOP+SPOP-3KR
D
shPIAS1 +EV
shPIAS1 +PIAS1-WT
shPIAS1 +PIAS1-S468A
shPIAS1 +PIAS1-S468D
E
F
shNC+EV
shPIAS1+EV
shPIAS1+PIAS1-WT
shPIAS1+PIAS1-S468A
shPIAS1+PIAS1-S468D

Supplementary Figure 10. (A) The comet assays revealed the overall DDR process in LNCaP cells under Olaparib treatment (2 μM), and the representative images (up) and statistical graph (down, n = 10) . The comet assays were then performed and 10 cells from each sample were analyzed based on the tail moment, utilizing the Komet software. **(B)** BRCA1 foci were stained (up) and corresponding statistical graph (down, n =5) under Olaparib (2 μM) treatment to determine the HR process in LNCaP cells. **(C)** Ku70 foci were stained (up) and corresponding statistical graph (down, n =5) under Olaparib (2 μM) treatment to determine the NHEJ process in LNCaP cells. **(D)** The comet assays revealed the overall DDR process in LNCaP cells under Olaparib treatment (2 μM), and the representative images (up) and statistical graph (down, n = 10) . The comet assays were then performed and 10 cells from each sample were analyzed based on the tail moment, utilizing the Komet software. **(E)** BRCA1 foci were stained (up) and corresponding statistical graph (down, n =5) under Olaparib (2 μM) treatment to determine the HR process in LNCaP cells. **(F)** Ku70 foci were stained (up) and corresponding statistical graph (down, n =5) under Olaparib (2 μM) treatment to determine the NHEJ process in LNCaP cells. *P<0.05, **P<0.01, **P<0.001.

Supplementary Table 1. Association between the *SPOP* gene mutations and clinic pathological features in PCa patients

Characteristics	SPOP-WT	SPOP-MUT	P value
n	44	6	
Pathologic T stage, n (%)			0.5682
T2	10 (20%)	2 (4%)	
T3&T4	34 (68%)	4 (8%)	
Clinical M stage, n (%)			0.4413
M0	40 (80%)	6 (12%)	
M1	4 (8%)	0 (0%)	
Pathologic N stage, n (%)			0.5940
N0	42 (84%)	6 (12%)	
N1	2 (4%)	0 (0%)	
Gleason score, n (%)			0.6466
6&7	25 (50%)	4 (8%)	
8&9&10	19 (38%)	2 (4%)	
PSA Level			0.6806
<10	8 (16%)	2 (4%)	
10–50	26 (52%)	3 (6%)	
>50	10 (20%)	1 (2%)	

Supplementary Table 2. The primers, shRNA and siRNA sequence, antibodies and chemicals

Primers sequence	
Gene	Sequence 5'-3'
SPOP-exon6-F	TTTTCTATCTGTTTTGGACAGG
SPOP-exon6-R	CAAAGCCACAACTTGTCAGTG
SPOP-exon7-F	TTTGCGAGTAACCCCAAAG
SPOP-exon7-R	CTCATCAGATCTGGGAACTGC
SPOP-L90V-F	AGACAGTGCATCTGATGAAGAGGAAGAAGAGCC
SPOP-L90V-R	CATCAGATGCACTGTCTATGGTTAGGTCAATCACTTCT
SPOP-K101I-F	GTGAAGTTCGGGCAATATTCAAATTCTCCATCCTGAATGC
SPOP-K101I-R	TATTGCCCGAACTTCACTCTTTGGACAGCTGA
PIAS1-S466A-F	CCATAGACGCTTCATCTGATGAAGAGGAAGAAGAGC
PIAS1-S466A-R	CAGATGAAGCGTCTATGGTTAGGTCAATCACTTCTACTT
PIAS1-S467A-F	AGACAGTGCATCTGATGAAGAGGAAGAAGAGCC
PIAS1-S467A-R	CATCAGATGCACTGTCTATGGTTAGGTCAATCACTTCT
PIAS1-S468A-F	GACAGTTCGCTGATGAAGAGGAAGAAGAGCCA
PIAS1-S468A-R	TTCATCAGCGAACTGTCTATGGTTAGGTCAATCACTT
PIAS1-S466D-F	CCATAGACGATTCATCTGATGAAGAGGAAGAAGAGC
PIAS1-S466D-R	CAGATGAATCGTCTATGGTTAGGTCAATCACTTCTACTT
PIAS1-S467D-F	AGACAGTGACTCTGATGAAGAGGAAGAAGAGCCA
PIAS1-S467D-R	CATCAGAGTCACTGTCTATGGTTAGGTCAATCACTTCT
PIAS1-S468D-F	GTTCAGATGATGAAGAGGAAGAAGAGCCATC
PIAS1-S468D-R	CCTCTTCATCATCTGAACTGTCTATGGTTAGGTCAATCA

shRNA and siRNA sequence	
Gene	Sequence 5'-3'
shPIAS1-1	CCGGGCAAATGGTTATGAGCCTTAGCTCGAGCTAAGGCTCATAACCATTTGCTTTTT
shPIAS1-2	CCGGGCAACTTTGTCTCCATCTACCCTCGAGGGTAGATGGAGACAAAGTTGCTTTTT
shSPOP	GGTTAGATGAAGAAAGCAAAGTTCAAGAGACTTTGCTTTCTTCATCTAACC

Antibodies and Chemicals				
No.	Name	Species	Cat No.	Source
1	Anti-Myc-HRP	Mouse	M192-7	MBL
2	Anti-FLAG-HRP	Mouse	M185-7	MBL
3	Anti-HA-HRP	Mouse	M180-7	MBL
4	Anti-SUMO1	Rabbit	A19121	Abclonal
5	Anti-SUMO2/3	Rabbit	A5066	Abclonal
6	Anti-γH2AX	Rabbit	AP0687	Abclonal
7	Anti-BRCA1	Rabbit	A11034	Abclonal
8	Anti-Ku70	Rabbit	A0883	Abclonal
9	Anti-CK-5	Rabbit	A11396	Abclonal
10	Anti-β-actin	Mouse	AC043	Abclonal
11	Anti-HA	Mouse	AE008	Abclonal

12	Anti-HIPK2	Rabbit	ab108543	abcam
13	Anti-53BP1	Rabbit	ab175933	abcam
14	Anti-AR	Rabbit	22089-1-AP	Proteintech
15	Anti-PIAS1	Rabbit	R383086	zenbio
16	Anti-Caspase-7	Rabbit	9492	CST
17	CHX		HY-B0713	MCE
18	Olaparib		T3015	TOPSCIENCE
19	IPTG		T4336	TOPSCIENCE
20	SPOP-IN-6b		T16922	TOPSCIENCE
21	Silmitasertib		T2259	TOPSCIENCE
22	Lambda Protein Phosphatase		P2316	Beyotime
23	Anti-γH2AX	Rabbit	ab81299	abcam
24	Anti-PIAS1-Ser468	Rabbit	custom-made	Biodragon
