# Supplementary material for: SPOP mutations increase PARP inhibitor sensitivity via CK2/PIAS1/SPOP axis in prostate cancer
Source: JCI Insight. 2025 Apr 22;10(8):e186871. doi: 10.1172/jci.insight.186871 (PMC12016936; doi:10.1172/jci.insight.186871)

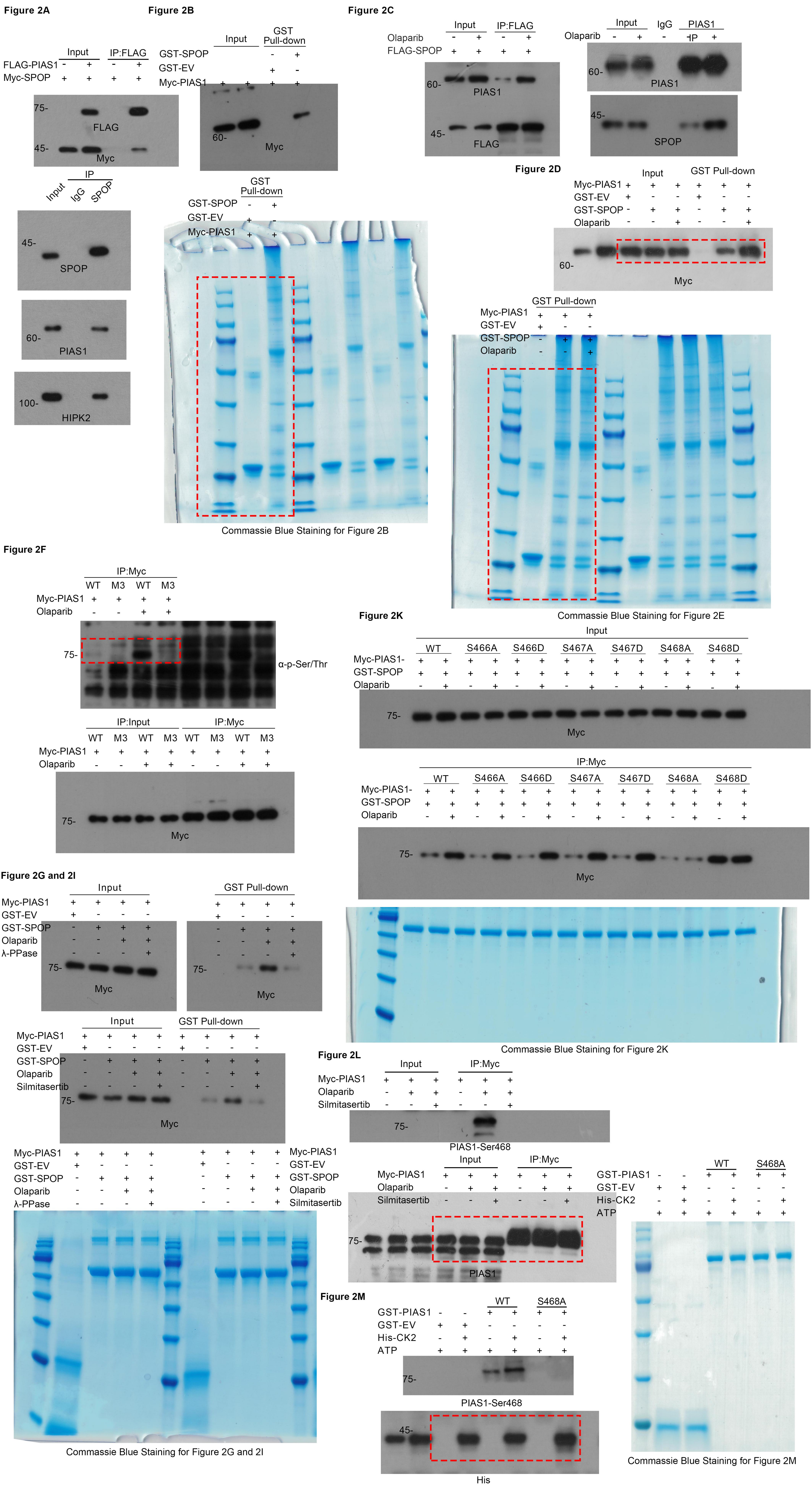

Figure 2A
Input
IP:FLAG
FLAG-PIAS1
Myc-SPOP
75-
FLAG
45-
Myc
IP
Input
IgG
SPOP
45-
SPOP
60-
PIAS1
100-
HIPK2
Figure 2B
Input
GST Pull-down
GST-SPOP
GST-EV
Myc-PIAS1
60-
Myc
GST Pull-down
GST-SPOP
GST-EV
Myc-PIAS1
Commassie Blue Staining for Figure 2B
Figure 2C
Input
IP:FLAG
Olaparib
FLAG-SPOP
60-
PIAS1
45-
FLAG
Input
IgG
PIAS1
Olaparib
60-
PIAS1
45-
SPOP
Figure 2D
Input
GST Pull-down
Myc-PIAS1
GST-EV
GST-SPOP
Olaparib
60-
Myc
GST Pull-down
Myc-PIAS1
GST-EV
GST-SPOP
Olaparib
Commassie Blue Staining for Figure 2E
Figure 2F
IP:Myc
WT
M3
WT
M3
Myc-PIAS1
Olaparib
75-
α-p-Ser/Thr
IP:Input
IP:Myc
WT
M3
WT
M3
WT
M3
WT
M3
Myc-PIAS1
Olaparib
75-
Myc
Figure 2K
Input
WT
S466A
S466D
S467A
S467D
S468A
S468D
Myc-PIAS1-
GST-SPOP
Olaparib
75-
Myc
IP:Myc
WT
S466A
S466D
S467A
S467D
S468A
S468D
Myc-PIAS1-
GST-SPOP
Olaparib
75-
Myc
Commassie Blue Staining for Figure 2K
Figure 2G and 2I
Input
GST Pull-down
Myc-PIAS1
GST-EV
GST-SPOP
Olaparib
λ-PPase
75-
Myc
75-
Myc
Input
GST Pull-down
Myc-PIAS1
GST-EV
GST-SPOP
Olaparib
Silmitasertib
75-
Myc
Myc-PIAS1
GST-EV
GST-SPOP
Olaparib
λ-PPase
Myc-PIAS1
GST-EV
GST-SPOP
Olaparib
Silmitasertib
Commassie Blue Staining for Figure 2G and 2I
Figure 2L
Input
IP:Myc
Myc-PIAS1
Olaparib
Silmitasertib
75-
PIAS1-Ser468
Input
IP:Myc
Myc-PIAS1
Olaparib
Silmitasertib
75-
PIAS1
Figure 2M
WT
S468A
GST-PIAS1
GST-EV
His-CK2
ATP
75-
PIAS1-Ser468
45-
His
WT
S468A
GST-PIAS1
GST-EV
His-CK2
ATP
Commassie Blue Staining for Figure 2M

Figure 3A

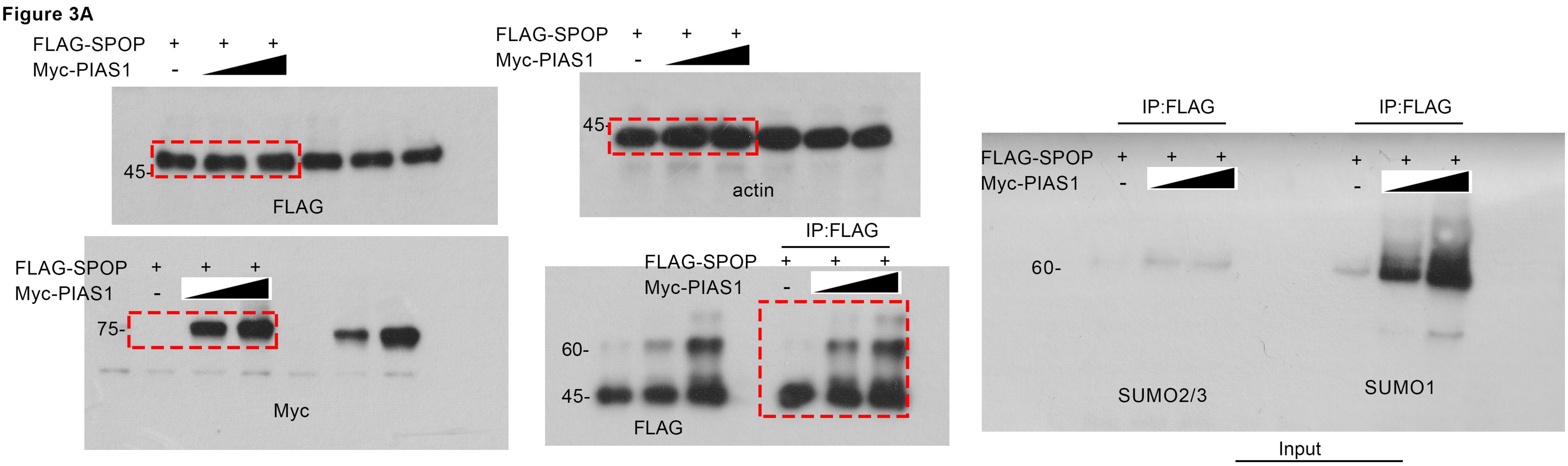

Figure 3B

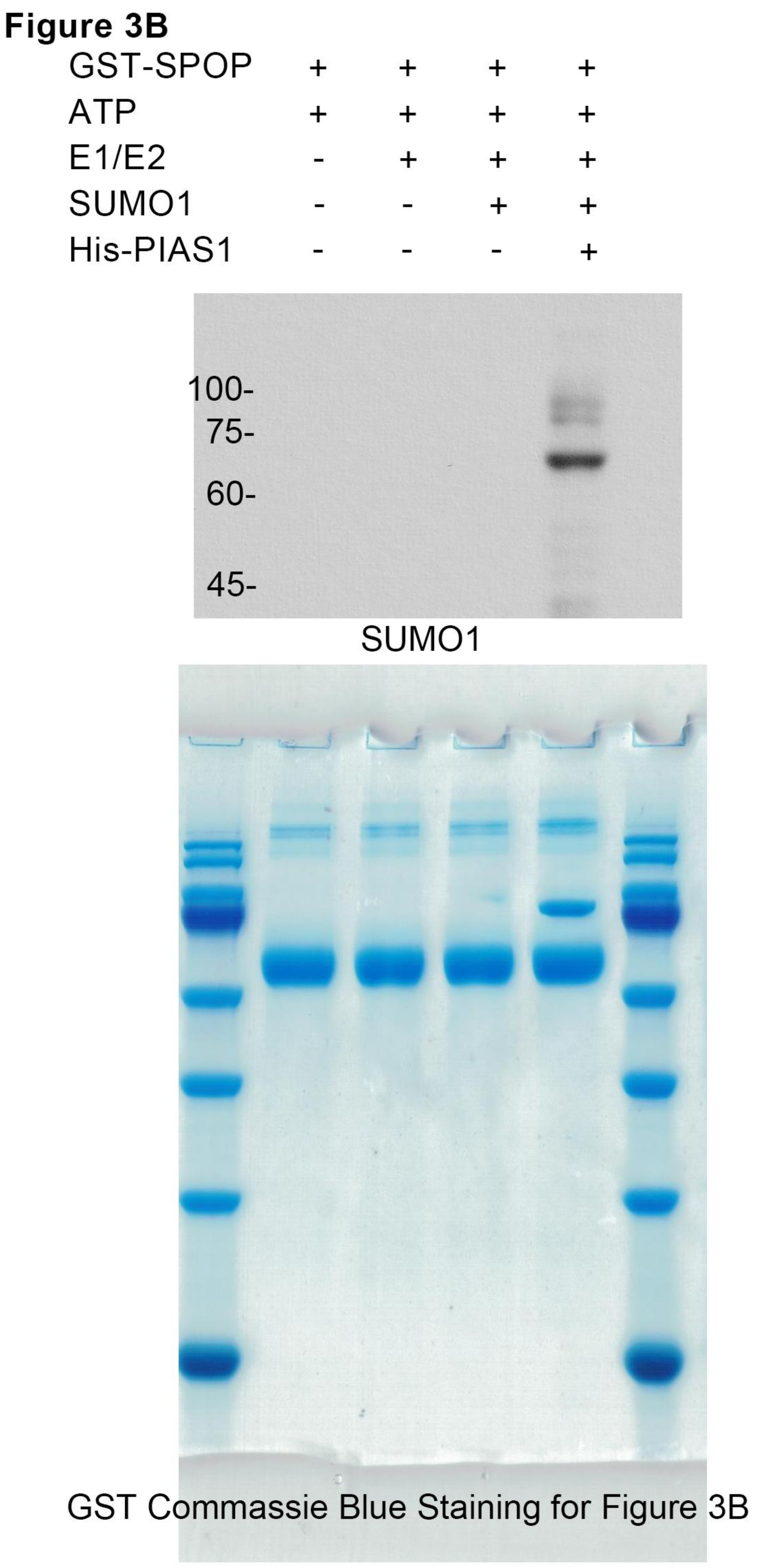

Figure 3C

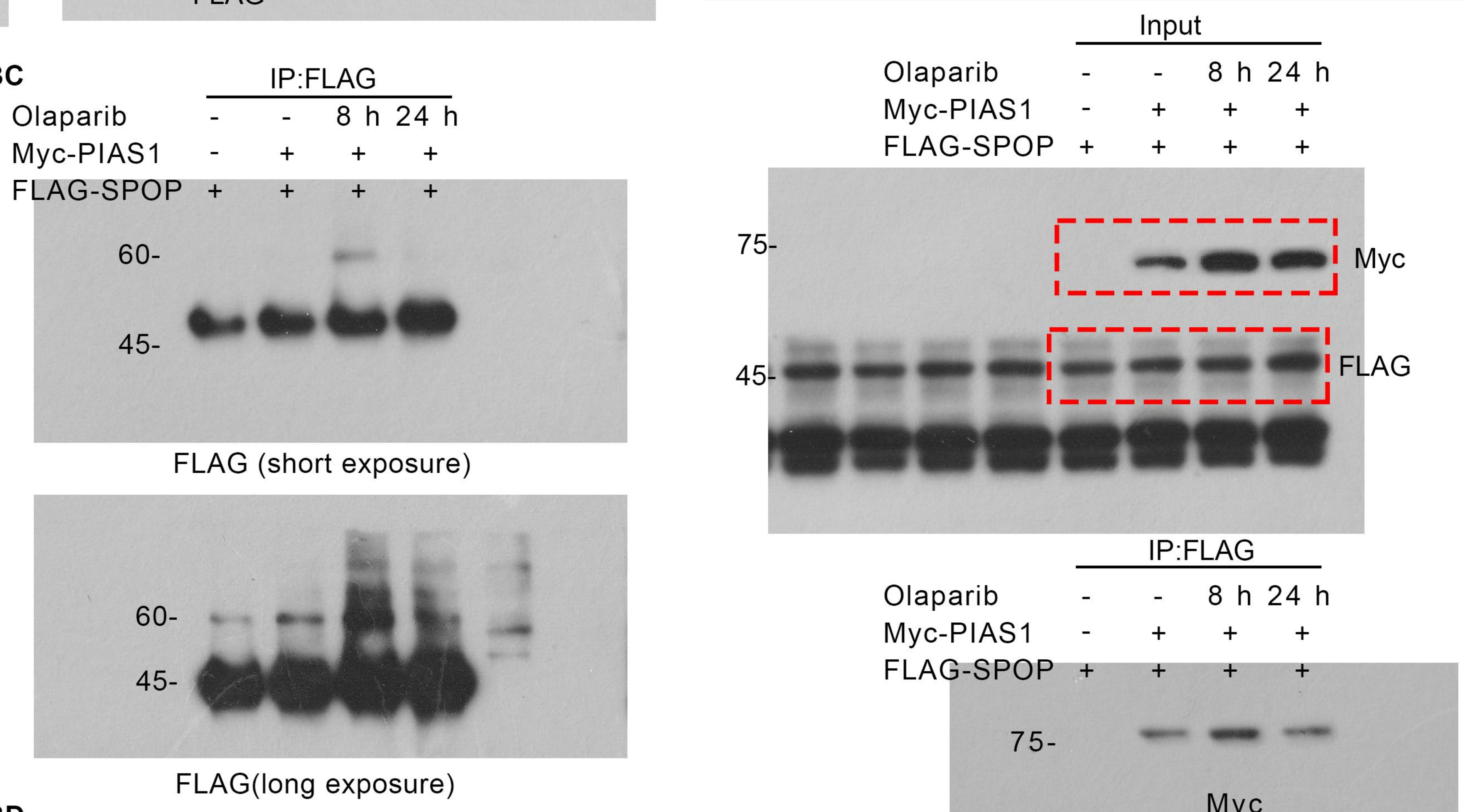

Figure 3D

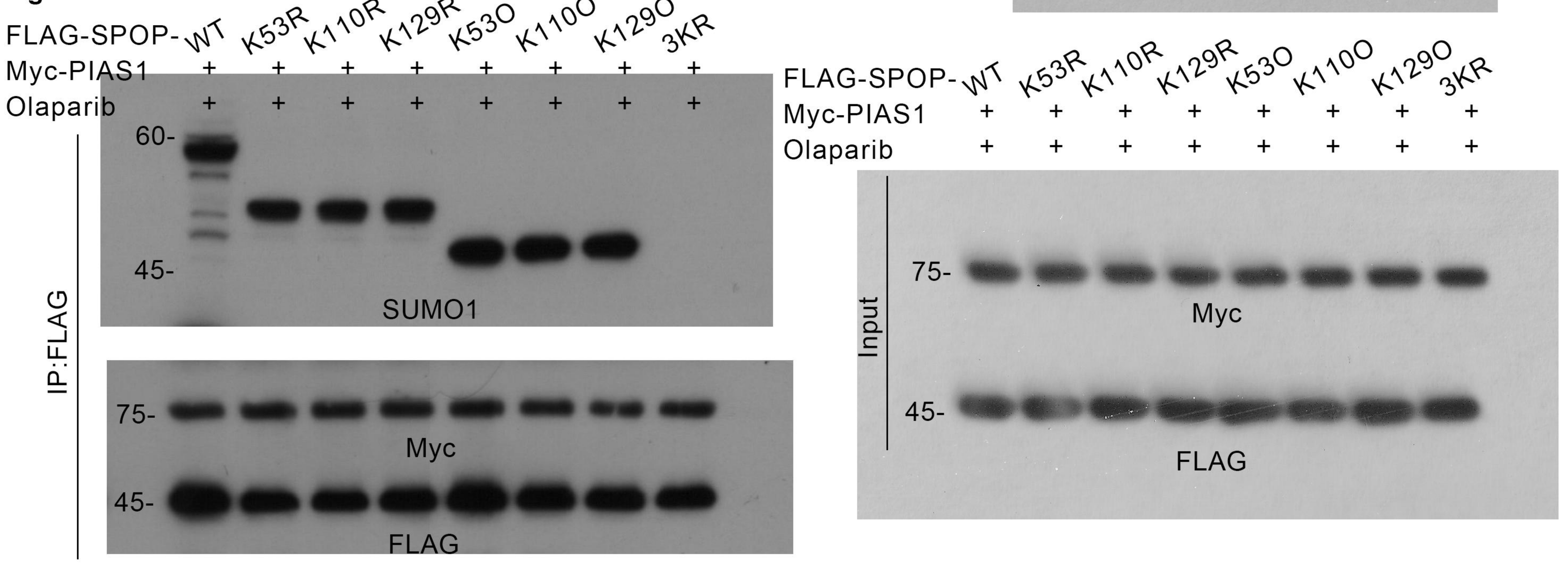

Figure 4A

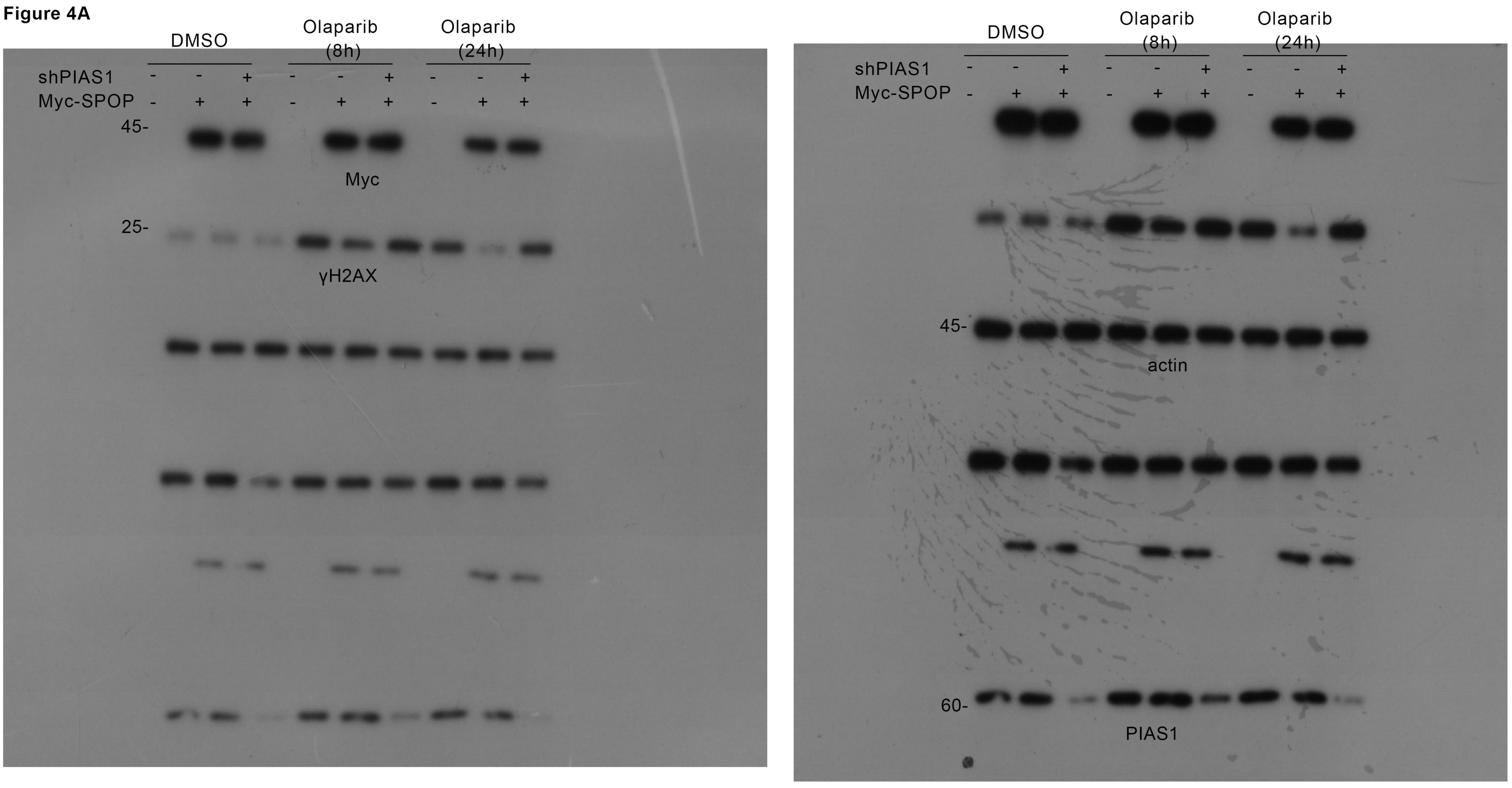

Figure 4D

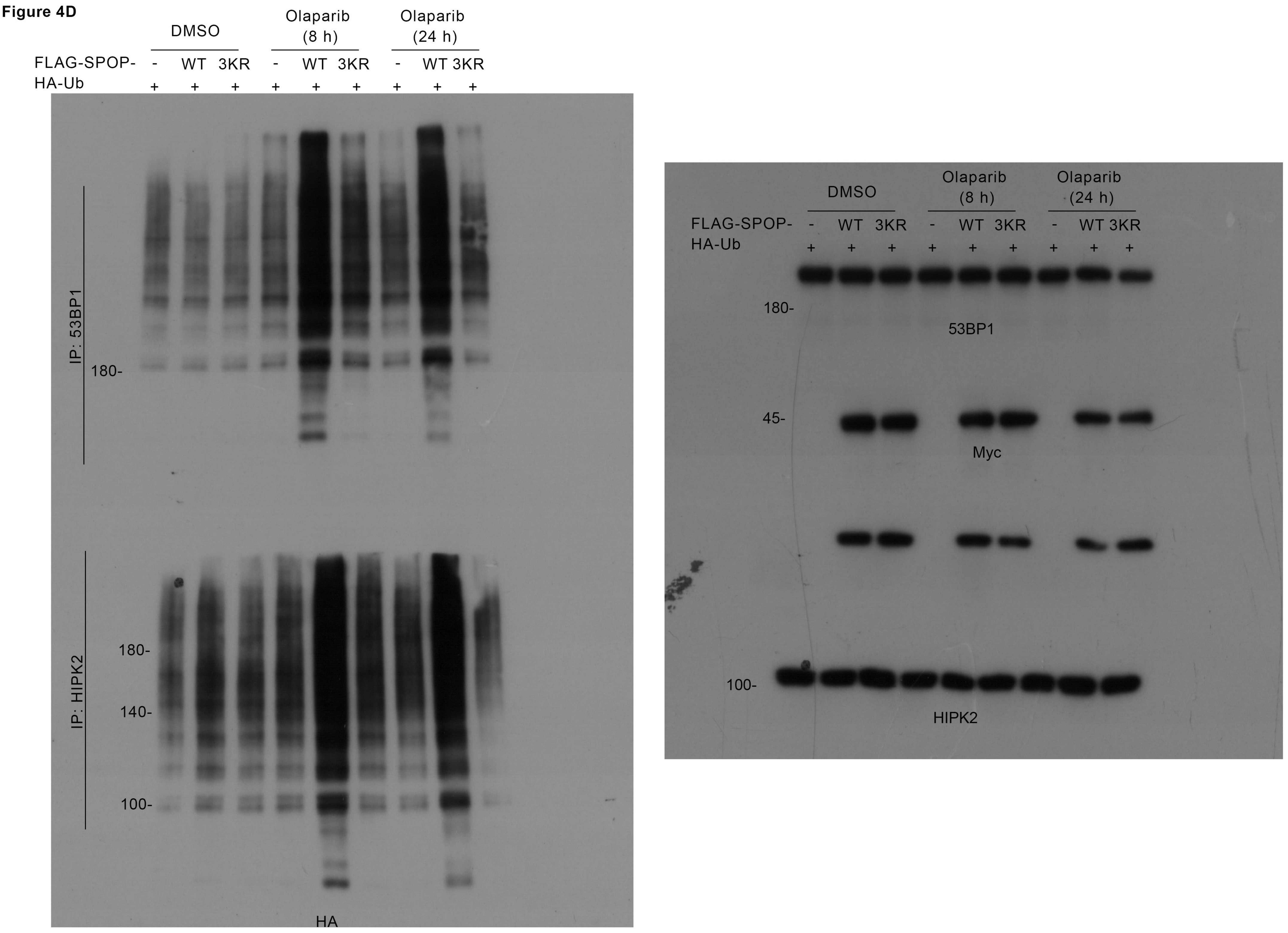

Figure 5B **Figure 5C**

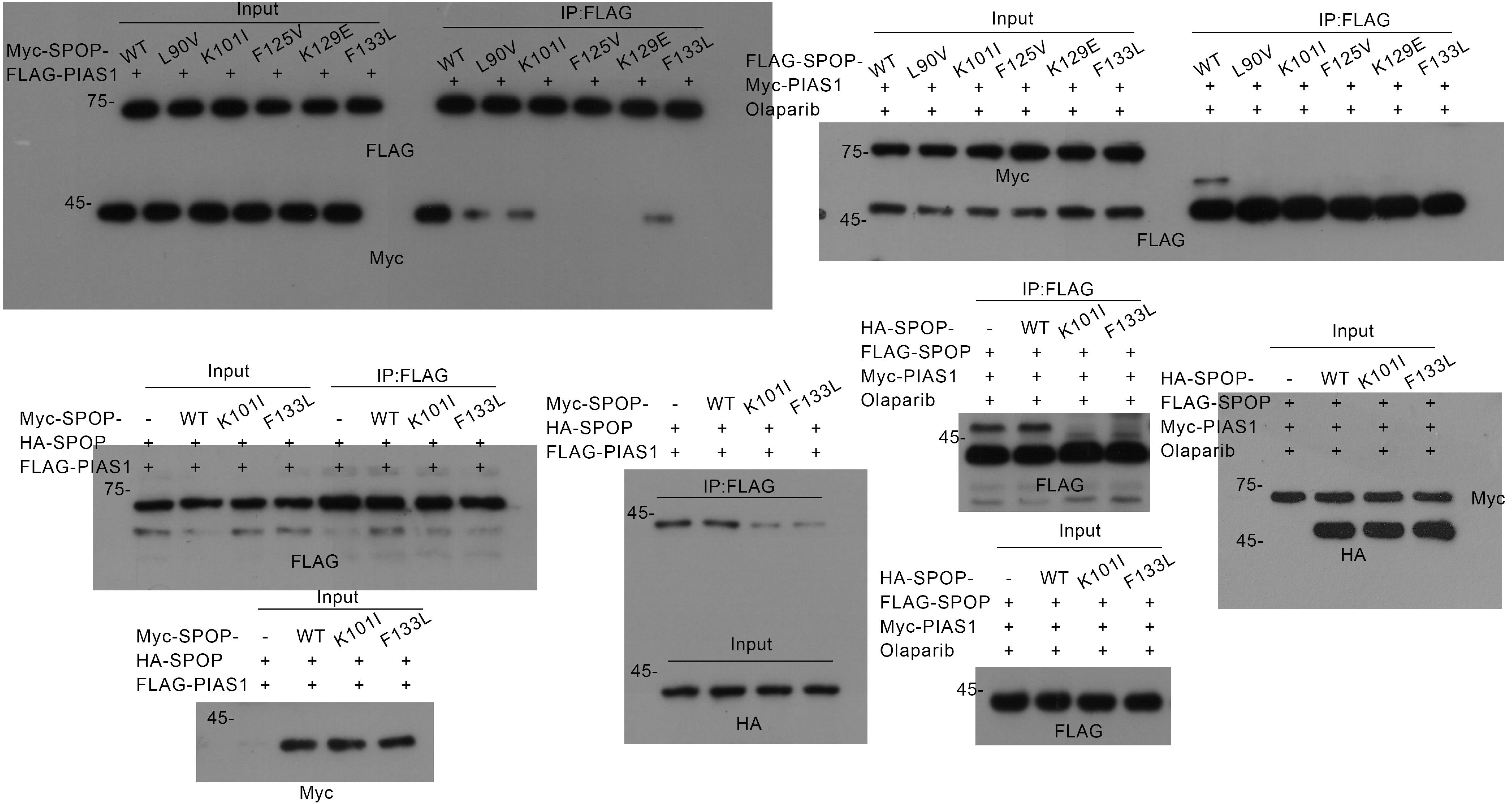

Supplementary Figure 1F

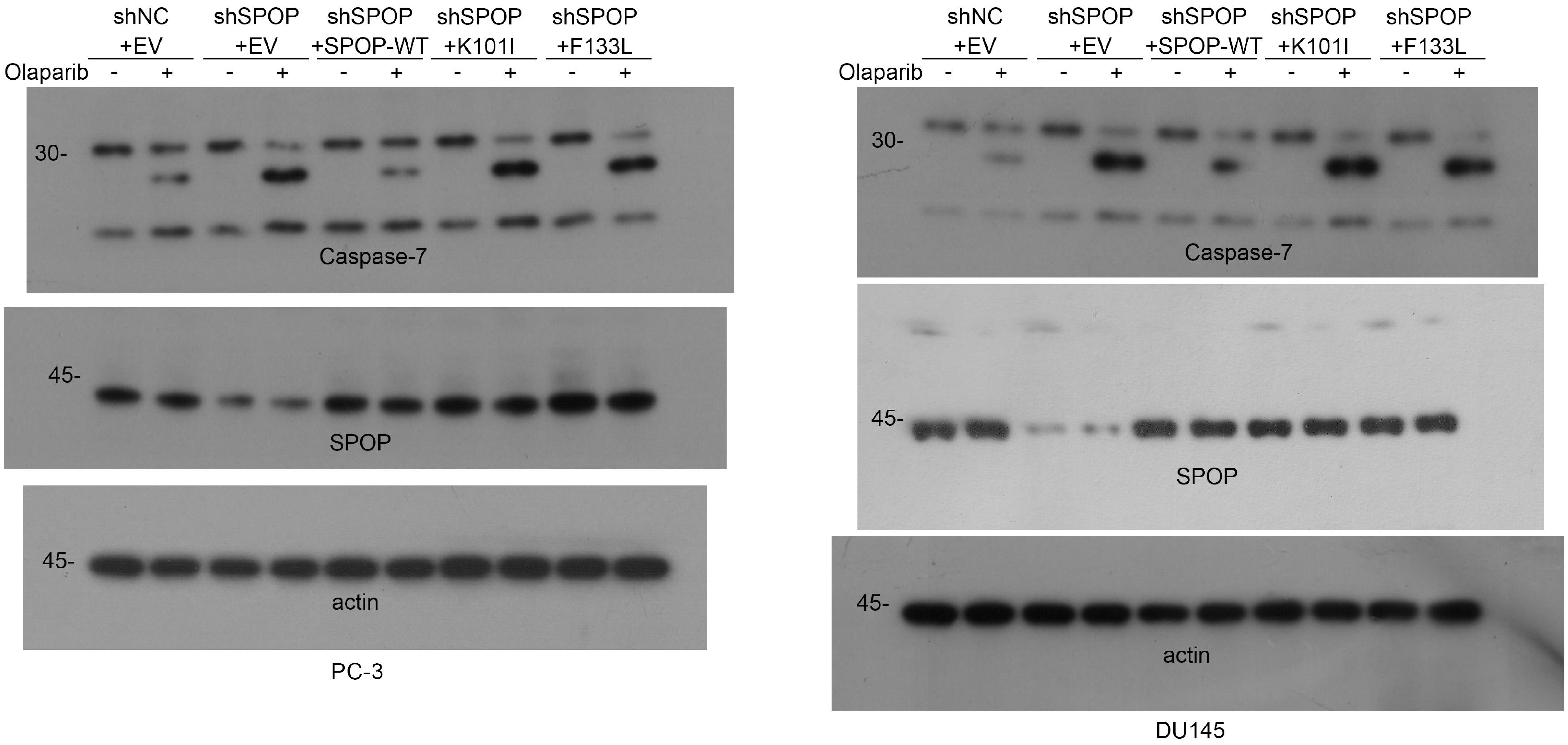

Supplementary Figure 4B

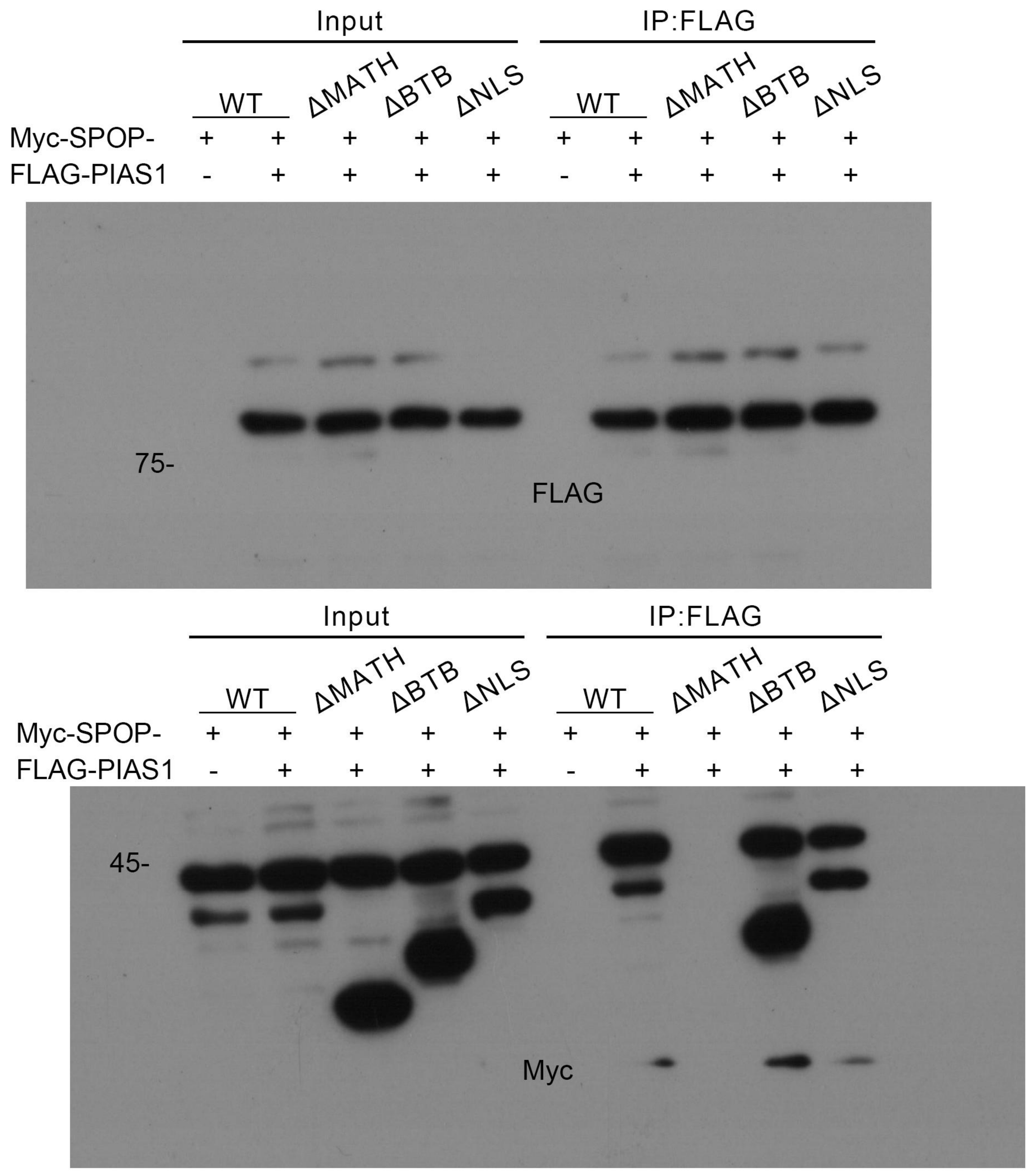

Supplementary Figure 4D

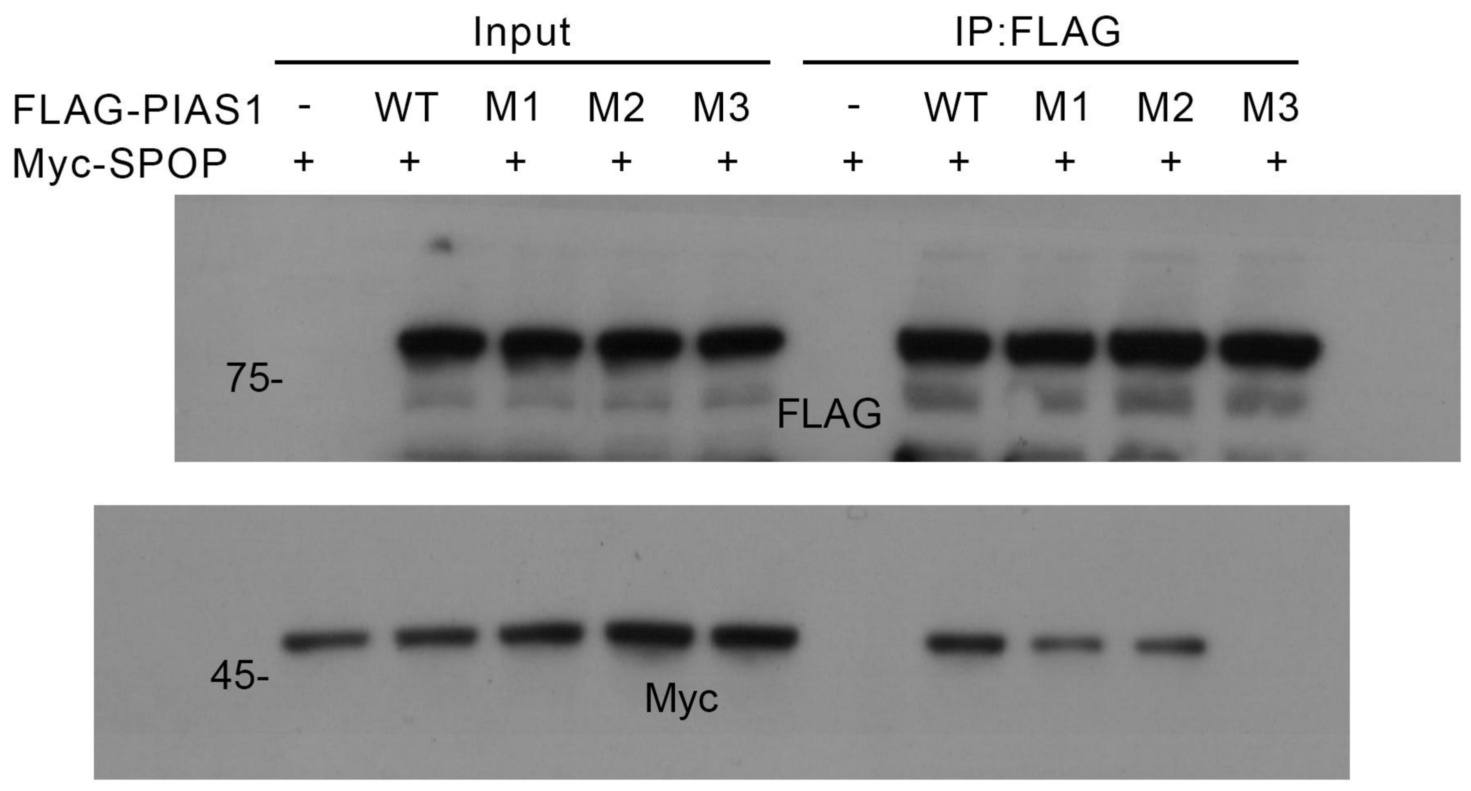

Supplementary Figure 4F

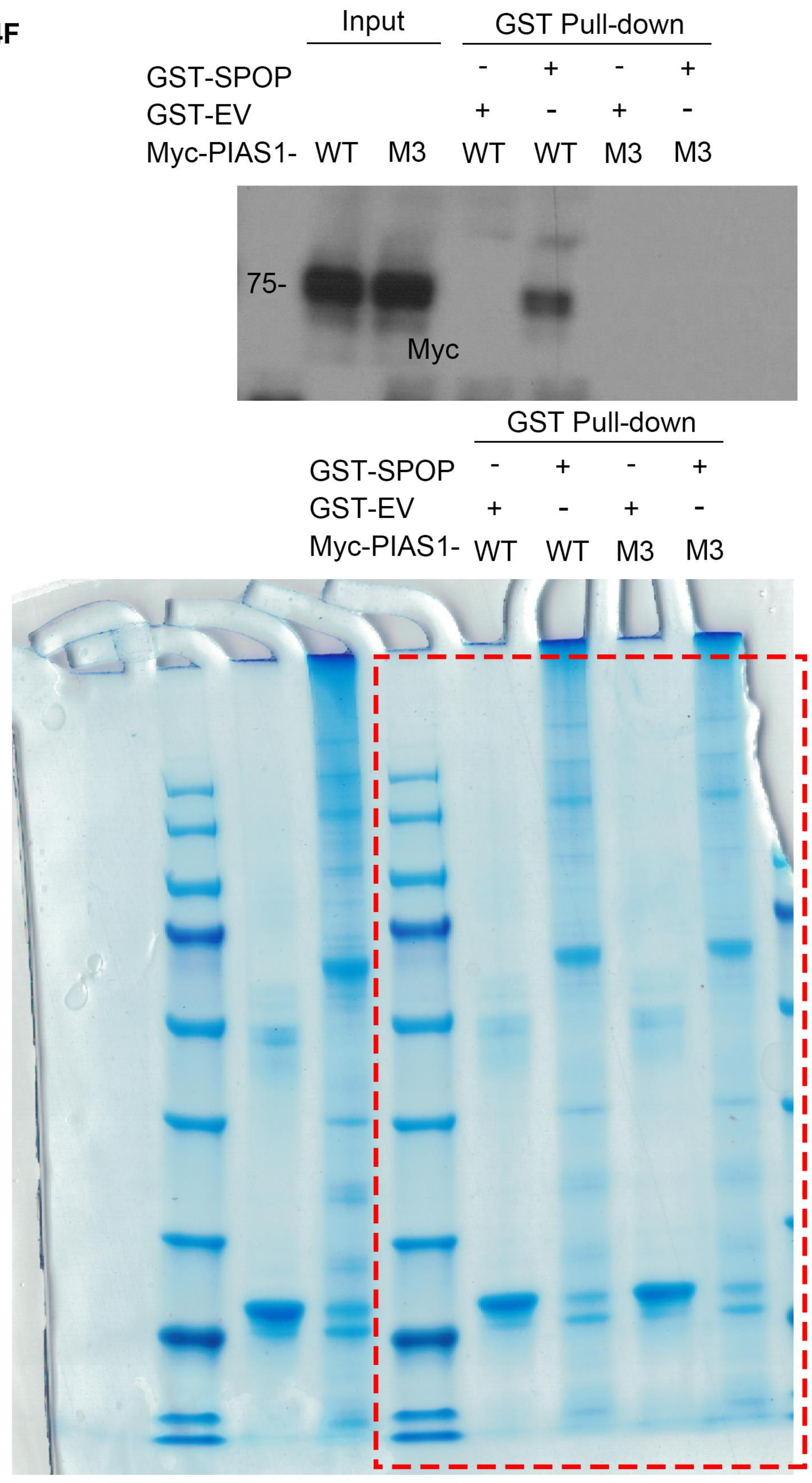

Commassie Blue Staining for Supplementary Figure 4F

Supplementary Figure 4F

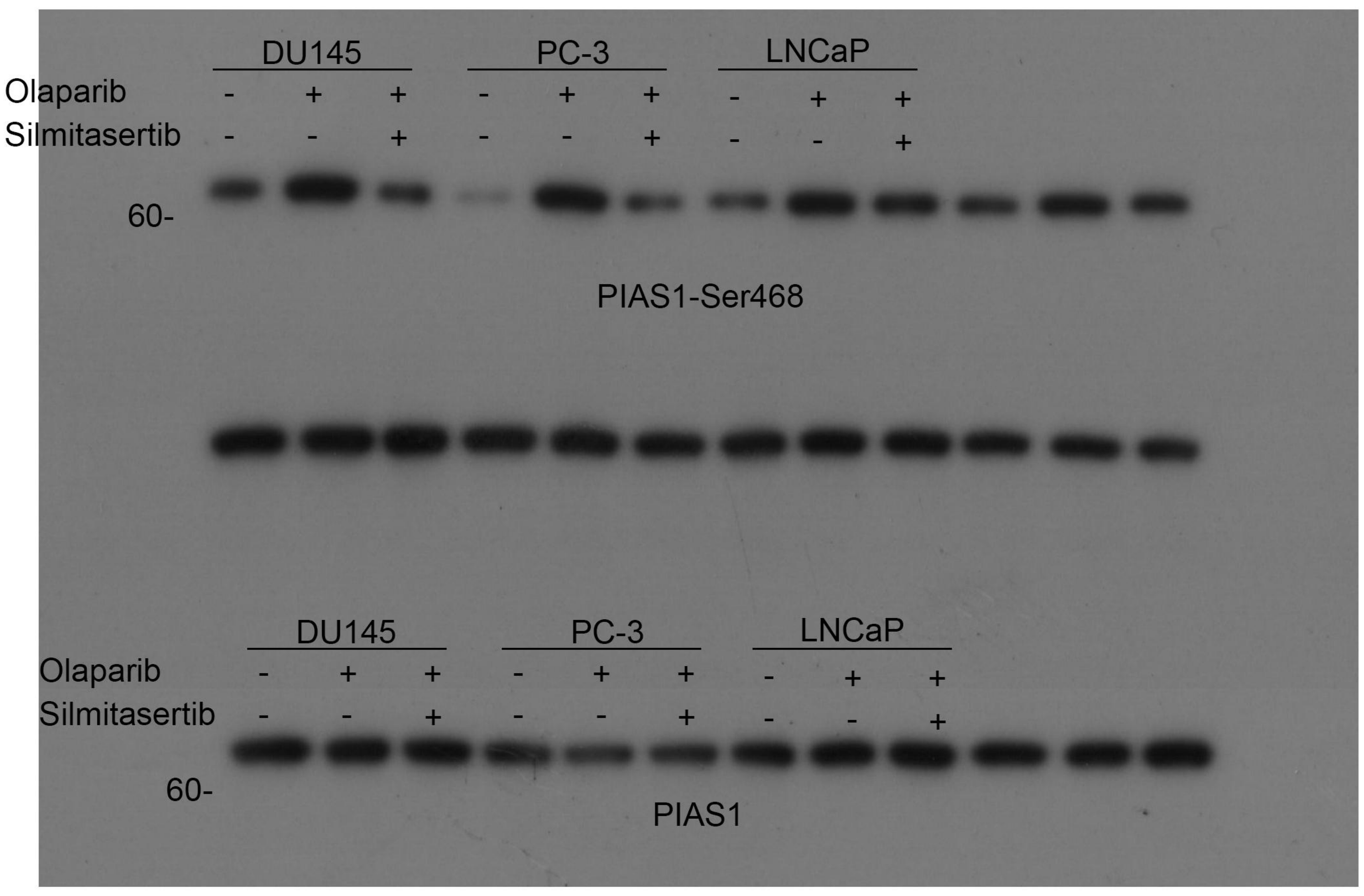

Supplementary Figure 4G and 4H

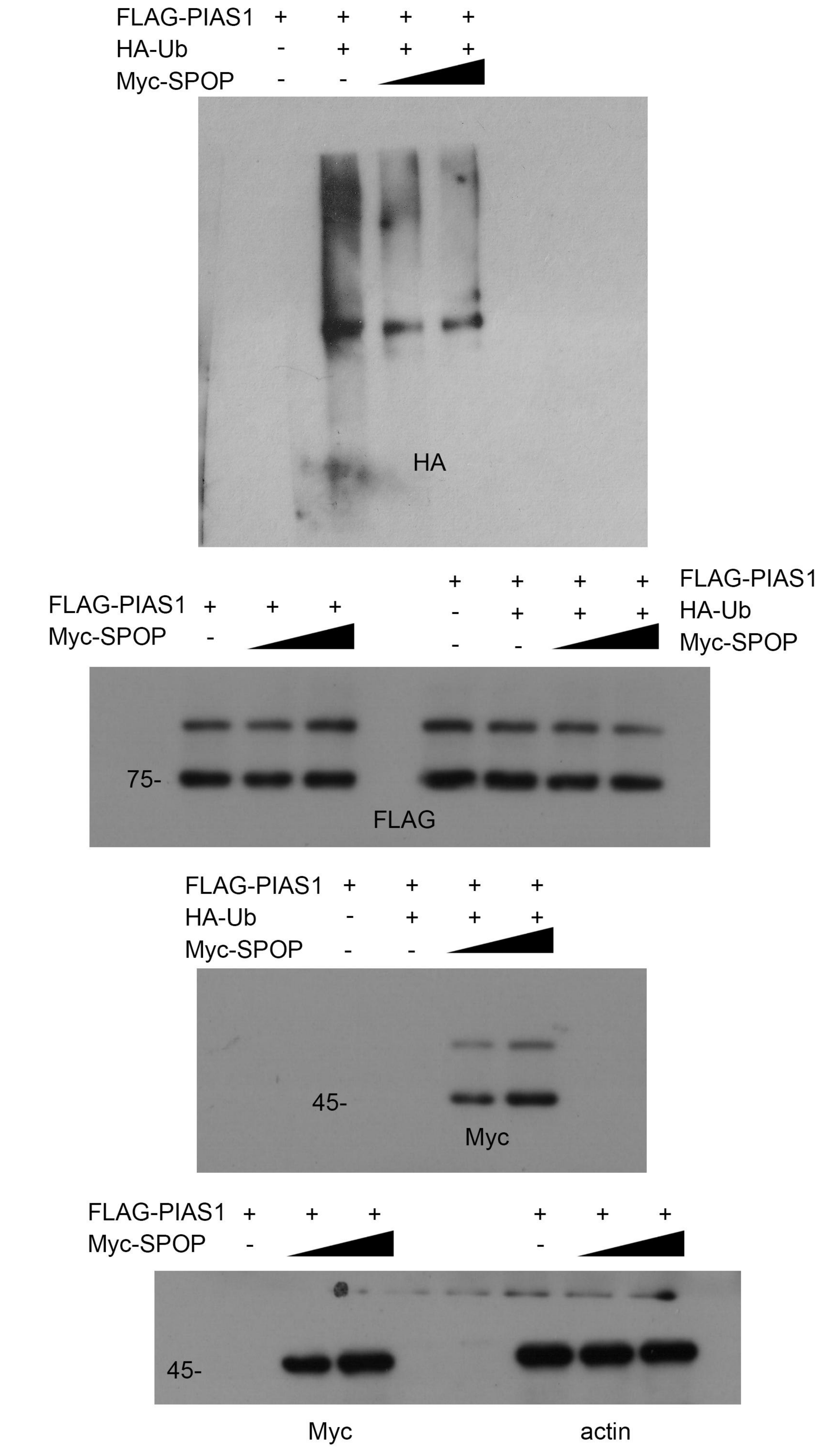

Supplementary Figure 4I

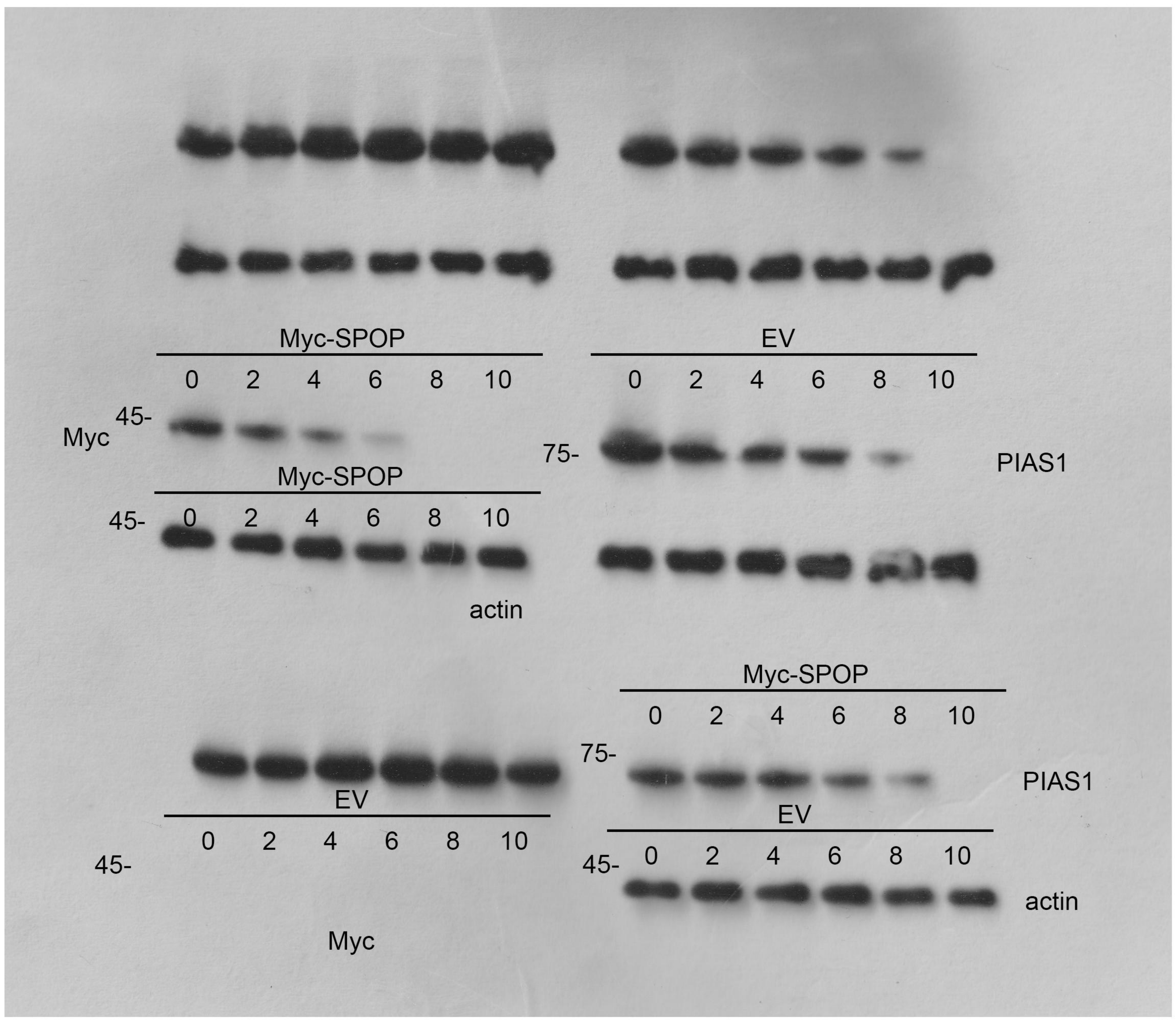

Supplementary Figure 5C

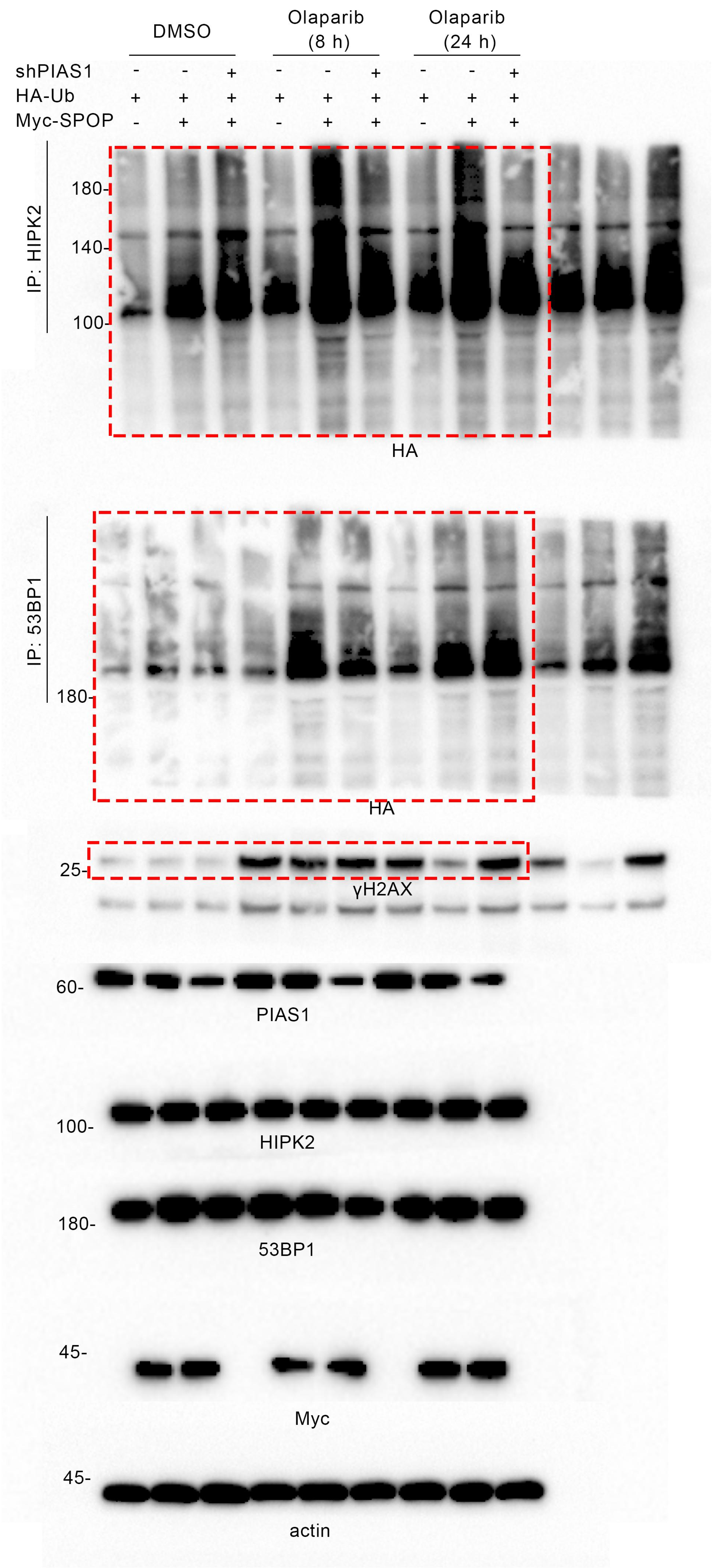

Supplementary Figure 5E

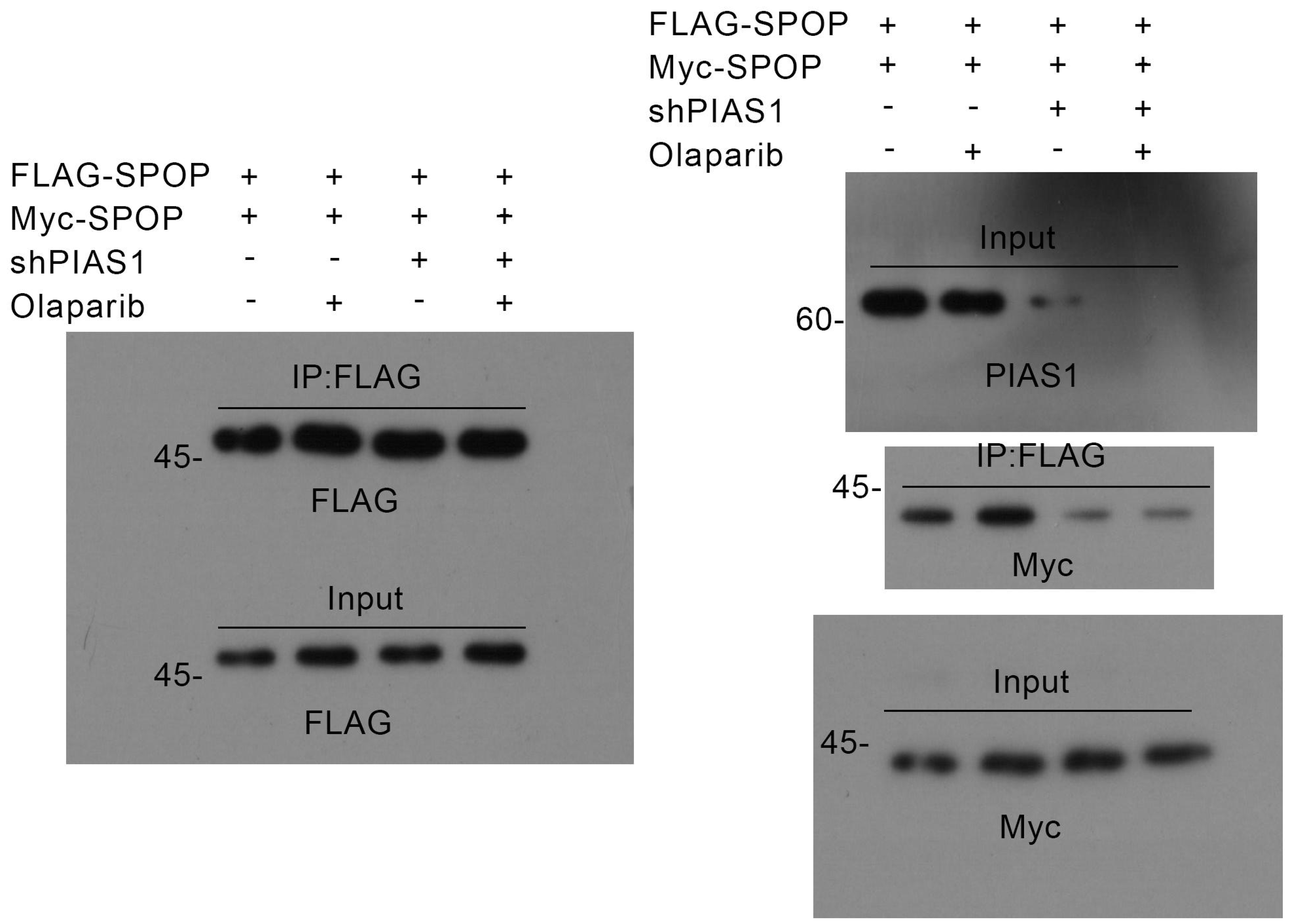

Supplementary Figure 5F

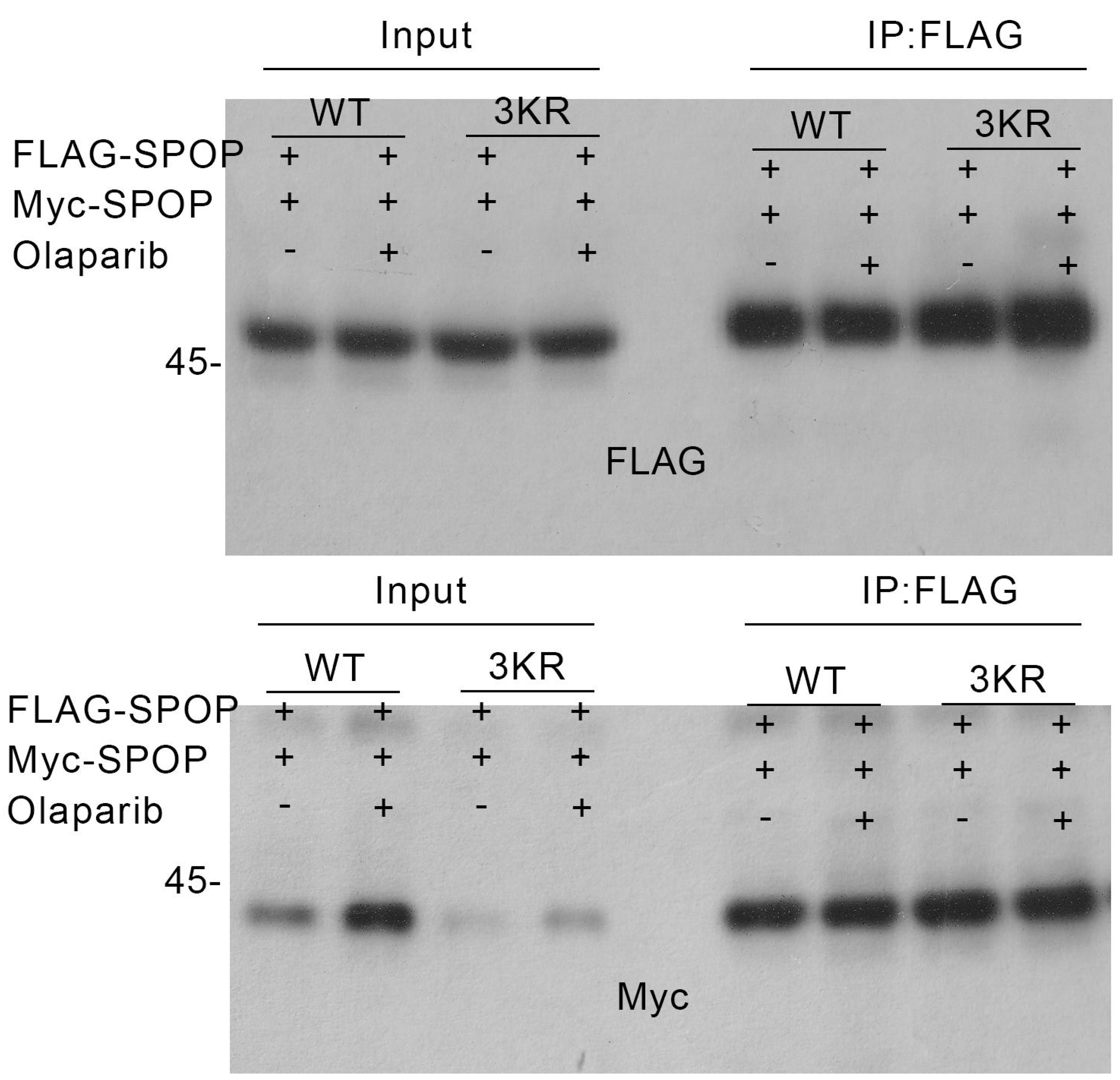

Supplementary Figure 6F

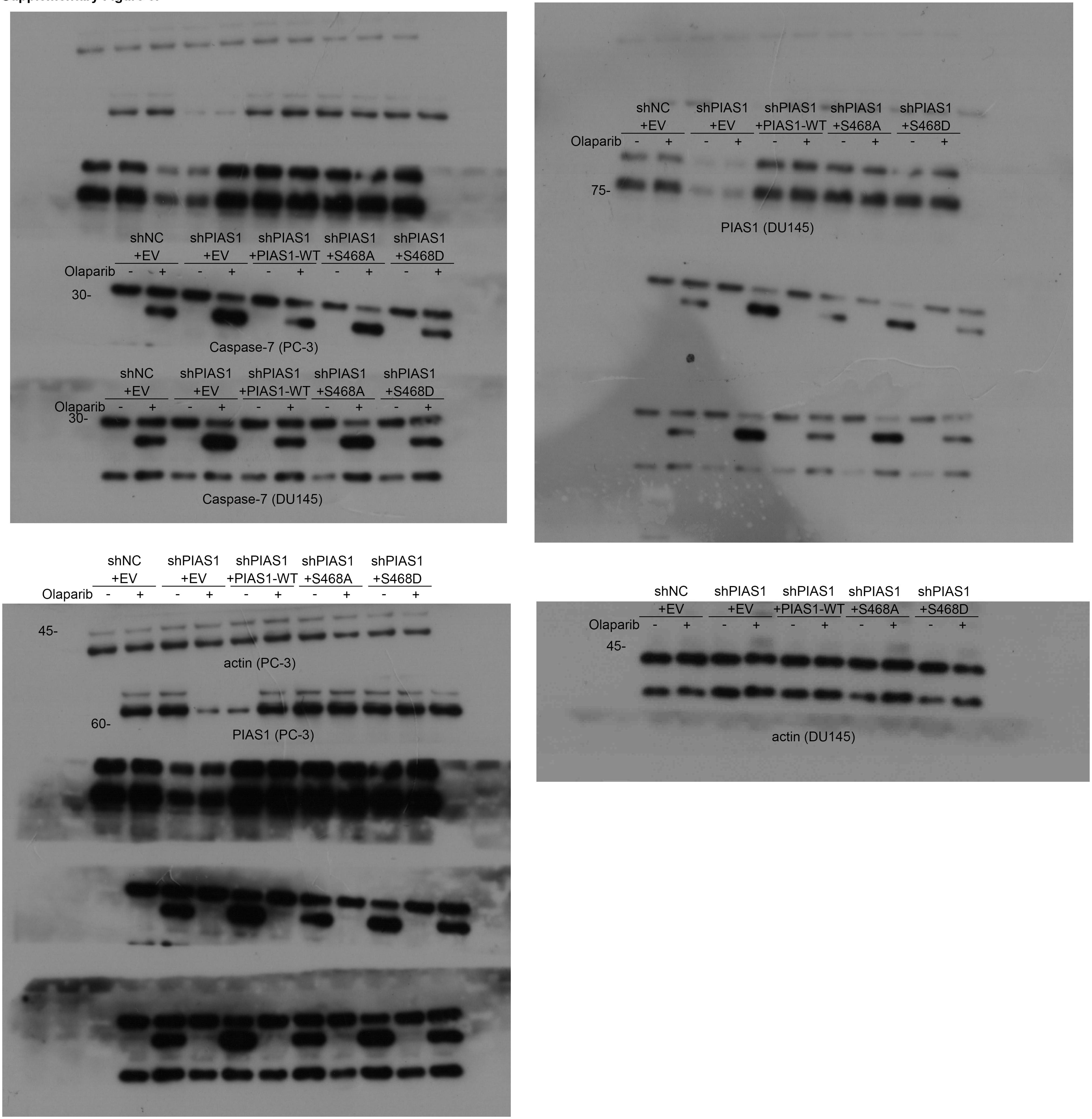

Supplement: Unedited blot and gel images [file jciinsight-10-186871-s014.pdf]
